# Temporal effectiveness of interventions to improve medication adherence: A network meta-analysis

**DOI:** 10.1371/journal.pone.0213432

**Published:** 2019-03-12

**Authors:** Elyssa Wiecek, Fernanda S. Tonin, Andrea Torres-Robles, Shalom I. Benrimoj, Fernando Fernandez-Llimos, Victoria Garcia-Cardenas

**Affiliations:** 1 Graduate School of Health, University of Technology Sydney, Sydney, New South Wales, Australia; 2 Pharmaceutical Sciences Postgraduate Programme, Universidade Federal do Paraná, Curitiba, Brazil; 3 Institute for Medicines Research (iMed.UL), Department of Social Pharmacy, Faculty of Pharmacy, University of Lisbon, Lisbon, Portugal; Universitat Witten/Herdecke, GERMANY

## Abstract

**Introduction:**

Adherence-enhancing interventions have been assessed in the literature, however heterogeneity and conflicting findings have prohibited a consensus on the most effective approach to maintain adherence over time. With the ageing population and growth of chronic conditions, evaluation of sustainable strategies to improve and maintain medication adherence long term is paramount. We aimed to determine the comparative effectiveness of interventions for improving medication adherence over time among adults with any clinical condition.

**Materials and methods:**

Meta-analyses evaluating interventions to improve medication adherence were searched in PubMed in January 2019 and reviewed for primary studies. Experimental studies with a comparison group assessing an intervention to enhance medication adherence in adult patients with reported adherence outcomes were included. Two authors extracted data for study characteristics, interventions and adherence outcomes. Interventions were categorized into four groups or combinations: educational, attitudinal, technical and rewards. Four network meta-analyses were performed to compare interventions based on patient follow-up time. Medication adherence effect sizes were reported as odds ratios (OR) with a 95% credibility interval (CrI) and surface under the cumulative ranking curve (SUCRA) to allow ranking probabilities. Risk of bias was assessed as per Cochrane guidelines.

**Results:**

Data was obtained from 69 meta-analyses with 468 primary studies being included in qualitative synthesis. The four networks compromised of 249 studies in total (0–3 month follow-up: 99 studies, 4–6 months: 104, 7–9 months: 18, ≥10 months: 94). Interventions showing success in follow-ups of less than 10 months varied across time. Significant effects compared to standard of care (SOC) were found in technical (4–6 months: OR 0.34, 95% CrI 0.25–0.45) and attitudinal interventions (7–9 months: 0.37, 0.17–0.84). Multicomponent interventions demonstrated effectiveness compared to standard of care with an additive effect displayed, particularly in longer follow-ups (educational + attitudinal + technical interventions ≥10 months: OR 0.49, 95% CrI 0.27–0.88).

**Discussion:**

All interventions reviewed improved medication adherence compared to standard of care. Multicomponent interventions displayed the most promising results in maintenance of long-term medication adherence. Technical and reward components enhanced adherence on a short-term basis, while educational and attitudinal interventions evolved over time to be more effective in follow-ups greater than 7 months. Sustainability of adherence to medications over time is dependent upon multicomponent interventions including educational, attitudinal and technical aspects to modify and enhance patient medication-taking behavior. Future research should focus on the most cost-effective approaches able to be integrated into routine practice.

## Introduction

A significant proportion of health care system funding is spent on medications, with 10% of U.S. national health expenditure attributed to prescription medications in 2016 [1]. As only 50% of patients reportedly take their medications as prescribed, medication non-adherence is a major challenge for the health care system [2]. Suboptimal adherence to prescribed medications not only increases health care costs but also increases the possibility of poor health outcomes, adverse events and hospitalizations [3, 4]. It is estimated that failure to adhere to medications results in $290 billion per year in unnecessary expenditure in the U.S. [5].

Adherence is not simply a matter of patient choice or will [6–10], but is affected by the interplay of multiple determinants of adherence that the World Health Organization (WHO) has classified into five different dimensions–condition-related factors, therapy-related factors, patient-related factors, socio-economic factors and healthcare team and system-related factors. While patient population characteristics may have an effect on adherence, determinants and barriers for non-adherence are often comparable across different medications and clinical conditions. [11–13].

Medication adherence can be conceptualized as having three major components: (1) initiation–when the patient takes the first dose of a prescribed medication; (2) implementation–the extent to which a patient’s actual dosing corresponds to the prescribed dosing regimen from initiation until the last dose is taken, and (3) persistence–the time from initiation to discontinuation [14]. Non-adherence can occur in any of these phases, and may change over time in patients. There is substantive evidence of a declining trend in adherence over time [15–18], and many determinants of non-adherence are found to be affected by time [6]. Time-related factors are particularly important for people with chronic diseases, where lifetime adherence to therapy may be required.

Numerous multifaceted adherence-enhancing interventions, ranging from simple educational material to multicomponent approaches integrating advanced behavioral and educational techniques, have been proposed and tested in a wide variety of settings, populations and clinical conditions, using a wide range of measures of adherence [11, 19, 20]. Recent meta-analyses have not reached a decisive conclusion, with some suggesting cognitive-educational interventions are effective [21] and others promoting habit-based strategies [19]. A Cochrane systematic review also concluded interventions’ effects were inconsistent across studies, however, they found the most effective interventions to be complex with frequent patient interaction [11]. Unfortunately there is additionally a lack of direct, head-to-head evidence of intervention strategies and combined with the complexity of the literature, makes it a challenge to select evidence-based interventions for implementation in routine clinical practice.

Network meta-analysis is a technique recommended by the International Society for Pharmacoeconomics and Outcome Research to compare efficacy among different interventions [22]. Compared with pairwise meta-analyses, it provides robust comparative evidence, allowing for estimates of relative treatment effects on both direct and indirect evidence [23]. This approach to evaluate all treatment options to each other simultaneously allows more optimal guidance on comparing interventions to other interventions rather than the common comparator of standard of care alone.

The aim of this systematic review and network meta-analysis was to analyze the comparative effectiveness of interventions for improving medication adherence over time among adults with any clinical condition.

## Materials and methods

The PRISMA extension to network meta-analysis and Cochrane Collaboration recommendations to design and report were used for this systematic review and network meta-analysis [24–26]. The review is registered on PROSPERO at CRD42018054598.

### Data sources

A systematic search of the medical literature was conducted for relevant meta-analyses comparing patient-targeted interventions to improve medication adherence in adult populations reporting adherence outcomes. The search was conducted on PubMed in January 2019 without any restriction based on publication date or language. The complete search strategy is available in S1 Table. Two investigators (EW, ATR) independently reviewed all abstracts and full-text articles and discrepancies were solved by a third reviewer. The primary studies included in the meta-analyses were then fully reviewed.

### Study selection

Primary experimental design studies with a comparison group that assessed an intervention with the objective of improving medication adherence in adult patients and which reported implementation adherence as an outcome using any measure (e.g. self-report, pill count, electronic monitoring) were included. Other active interventions or standard of care were considered as comparators. Unpublished studies, articles written in non-Roman characters, with pediatric populations (<18 years), assessing interventions targeted at healthcare professionals or studies using other types of treatment (over-the-counter medications, depot medications, vaccines) were excluded. Studies were not restricted by country, clinical condition or trial follow-up. Eligible primary studies with categorical medication adherence outcomes (i.e. adherent vs non-adherent) were included in the network meta-analyses while those with continuous outcomes were only included in the qualitative analysis.

### Data extraction and quality assessment

The following data from primary articles was extracted by two investigators (EW, ATR) using a standard data sheet piloted with 28 studies: study baseline characteristics (authors, year, title, sample size, clinical condition, demographics, duration of study, evaluated interventions), study design, measure of adherence used, variable type (continuous versus categorical) and corresponding adherence rates before and after the intervention.

To standardize the results obtained from different measures of adherence, an overall composite adherence outcome was used for categorical variables that represented the rate of adherent patients obtained from any of the measures in each study. The overall composite score was validated by Tonin et al, 2018 [27]. If a study included more than one measure, a mean rate from the different measures of adherence was calculated.

According to the patient follow-up period of each included study, results were grouped based on patient follow-up and results of adherence reported into standardized periods of time: 0–3 months, 4–6 months, 7–9 months, and ≥ 10 months.

To improve interpretability, interventions were grouped into four categories: attitudinal components aiming to modify beliefs, reward components creating incentives, educational components to inform on the medication, disease state, or importance of adherence, and technical components intended to simplify the medication taking process. The development and categorization process was discussed in Tonin et al, 2018 [27], and full category definitions can be found in S2 Table. Multicomponent interventions included more than one single category (e.g. rewards + technical). Standard of care was considered as the usual care defined in the primary study.

Two reviewers (EW, ATR) assessed all articles using the Cochrane Risk of Bias tool [28]. Given the complexity of interventions and to avoid a floor effect, adjusted criteria for judgement of risk of bias were used. The adjusted criteria allowed for low risk of bias indicated if outcomes were not blinded but were measured with validated instruments (i.e. previously validated medication adherence questionnaires).

### Data analysis

Network meta-analysis was performed using Bayesian framework to analyze the comparative adherence of all the interventions for the overall composite measure of categorical measures in each time period. Interventions were modelled as they were described in the original studies, that is, as different combinations of components. Only implementation adherence outcomes could be used for comparison purposes. For all comparisons, a common heterogeneity parameter was assumed, and a conservative analysis of non-informative priors was chosen [29, 30]. Effect sizes measures were expressed as odds ratio (OR) with a 95% credibility interval (CrI). Heterogeneity between trial comparisons was estimated by using the I^2^ statistic. Both random and fixed effect models were tested. The goodness of fit of the model was assessed using residual deviances (DIC). Models with lowest DIC were used. Convergence was attained based on visual inspection of Brooks-Gelman-Rubin plots and potential scale reduction factor—PSRF (1<PSRF≤1.05) [30, 31]. To increase the estimate precision of the relative effect sizes of comparisons and to account properly for correlations between multi-arm trials, rank probabilities involving all the interventions were built for each outcome. The surface under the cumulative ranking curve (SUCRA) analysis was performed to present results of ranking order. SUCRA values can range from 0% (i.e. the intervention always ranks last) to 100% (i.e. the intervention always ranks first) [32]. Node-splitting analyses were used to assess inconsistency in the networks (p-values<0.05 reveal significant inconsistencies in the network) [33]. All analyses were performed using software Addis version 1.17.6 [34]. Other sensitivity analyses with the hypothetical removal or inclusion of the studies were conducted based on article’s year of publication (before or after 2007) and sample size (total number of included patients over 30).

## Results

**Fig 1:**
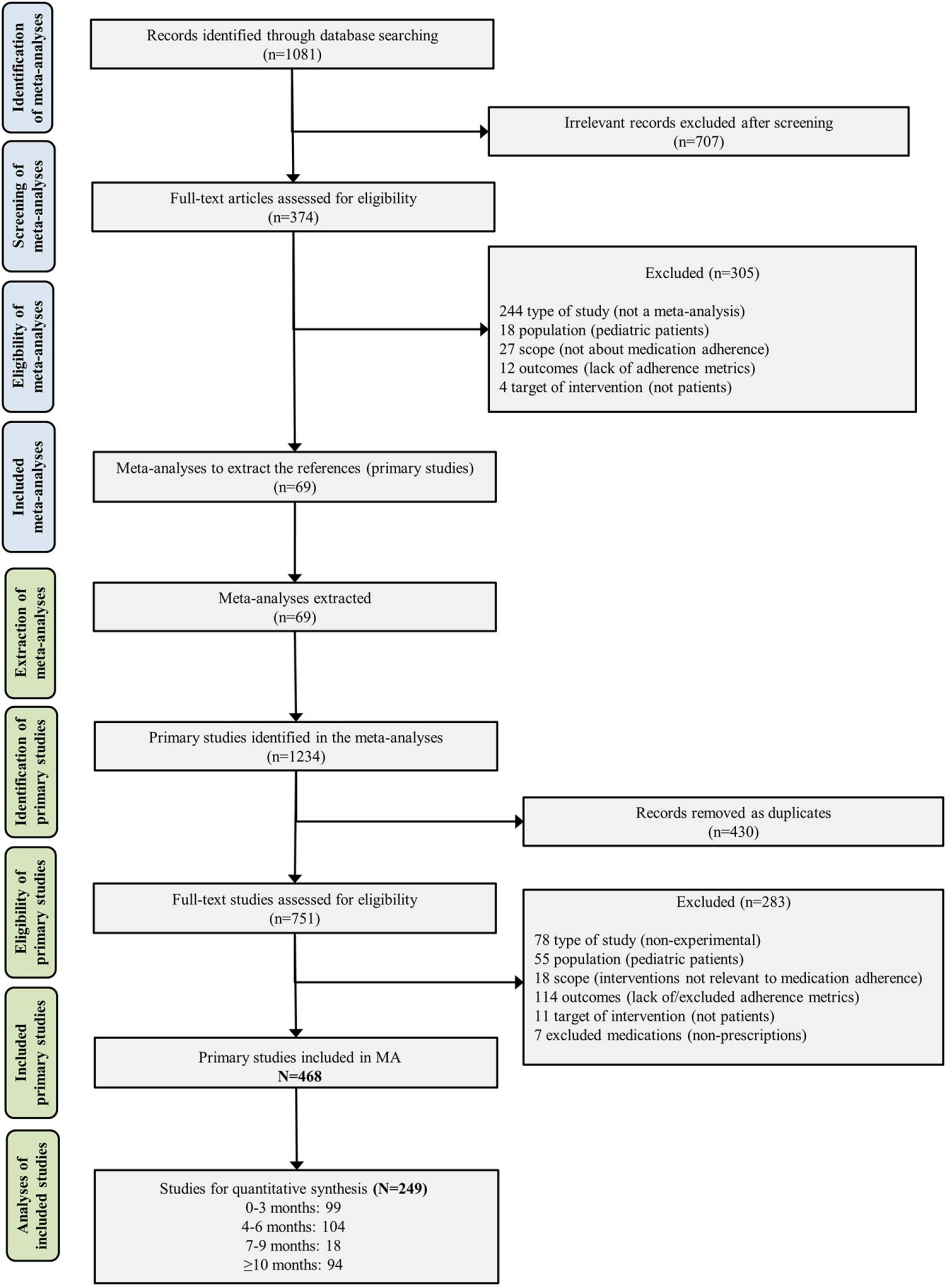
Flowchart of the systematic review process and included studies.

Database searching identified 1081 records. Sixty-nine meta-analyses were included for primary study extraction. From these, 1234 primary studies were identified and 468 studies were included for qualitative synthesis (see Fig 1) (S1 Appendix). The most common single interventions were educational (n = 172 studies), followed by technical (n = 118), attitudinal (n = 57) and rewards (n = 2). Combinations of two or more categories of interventions were found in 191 trials (41.0%) with most of them reporting educational + technical (n = 94), educational + attitudinal (n = 62) and educational + attitudinal + technical (n = 25). Standard of care was the common comparator in 88.0% of studies. The earliest studies were published in 1971 and the most recent in 2017 (median = 2008; IQR 2002–2012). Twelve clinical conditions were included with the most common being cardiovascular (n = 206 studies) and HIV (n = 96).

Risk of bias assessment revealed most studies having an unclear risk of bias. The domains with higher risk of bias were attrition bias (around 25% of studies) and performance bias (30% of studies) as studies lacked complete outcome data or were unable to blind participants due to the nature of the interventions. More than 90% of studies were free of selective reporting. Fewer than 10% of trials were sponsored by industries or presented conflict of interest (S1 Fig and S3 Table).

**Fig 2:**
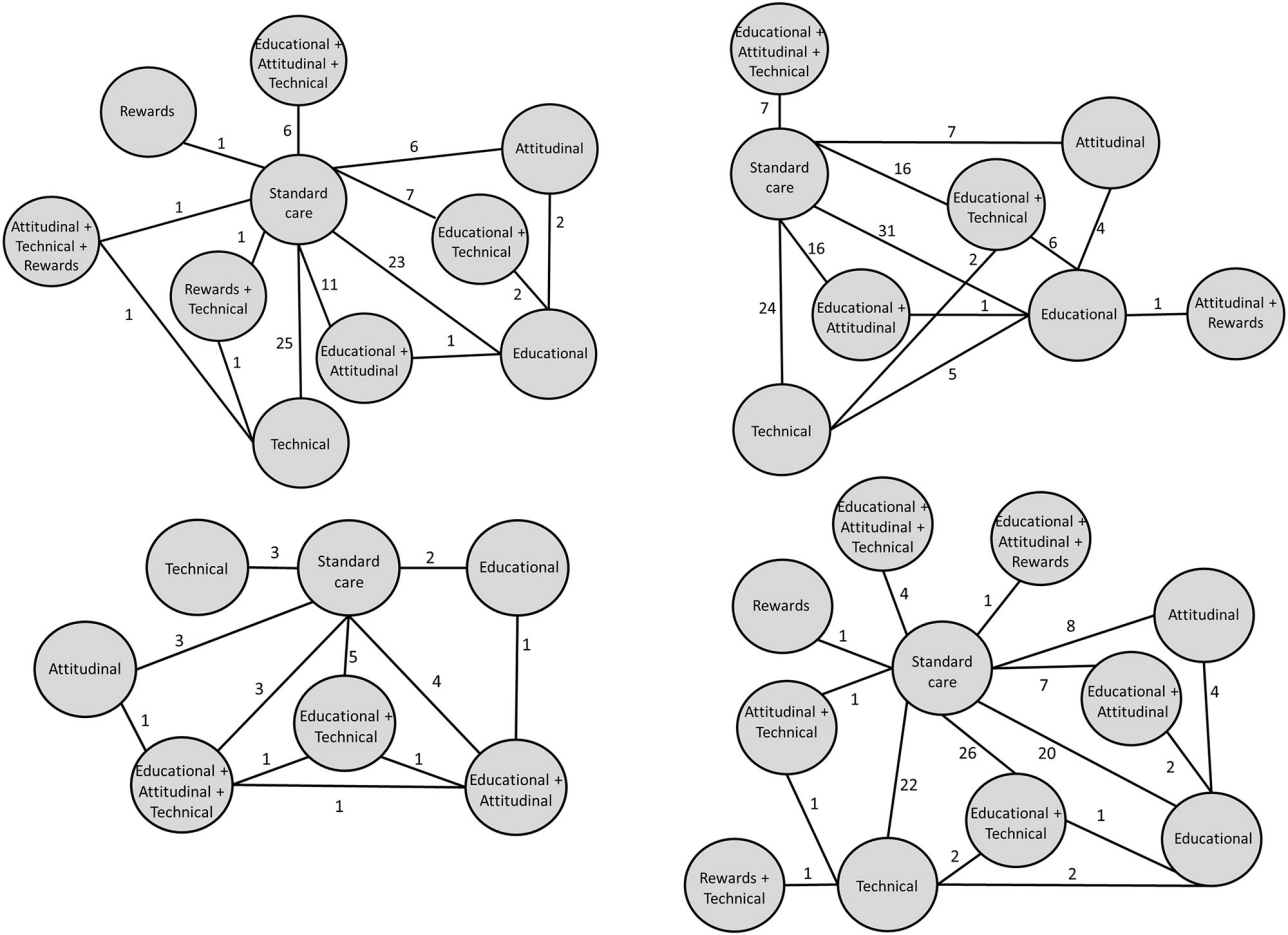
Networks of the comparisons between interventions for each time period (0–3 months, 4–6 months, 7–9 months, ≥10 months) considering the overall composite measure of adherence. Each node represents an intervention. Directly comparable interventions are linked with a line, the number of trials for each comparison are shown in each line.

For the quantitative network analyses, 219 studies were excluded due to the absence of categorical data on patient’s adherence. Another 11 trials were excluded as intervention arms were grouped in the same category and were unable to be compared in the network (e.g. technical vs. technical). Finally, 249 studies were included in the network meta-analyses of overall composite measure for the four periods of time with studies able to be included in more than one time period: 99 studies reporting results in the 0–3 month follow-ups, 104 in the 4–6 months, 18 in 7–9 months, 94 in ≥10 months. Seventy-one studies reported in more than one time period. Six interventions, in addition to standard of care, were evaluated in all the four time periods: attitudinal, educational, educational + attitudinal, educational + attitudinal + technical, educational + technical, and technical. The network plots of each time period with nodes representing the interventions are presented in Fig 2. Heterogeneity between trials for the composite measure analysis was moderate for the majority of the comparisons (81.3% I^2^ < 70%) (S4 Table).

**Fig 3:**
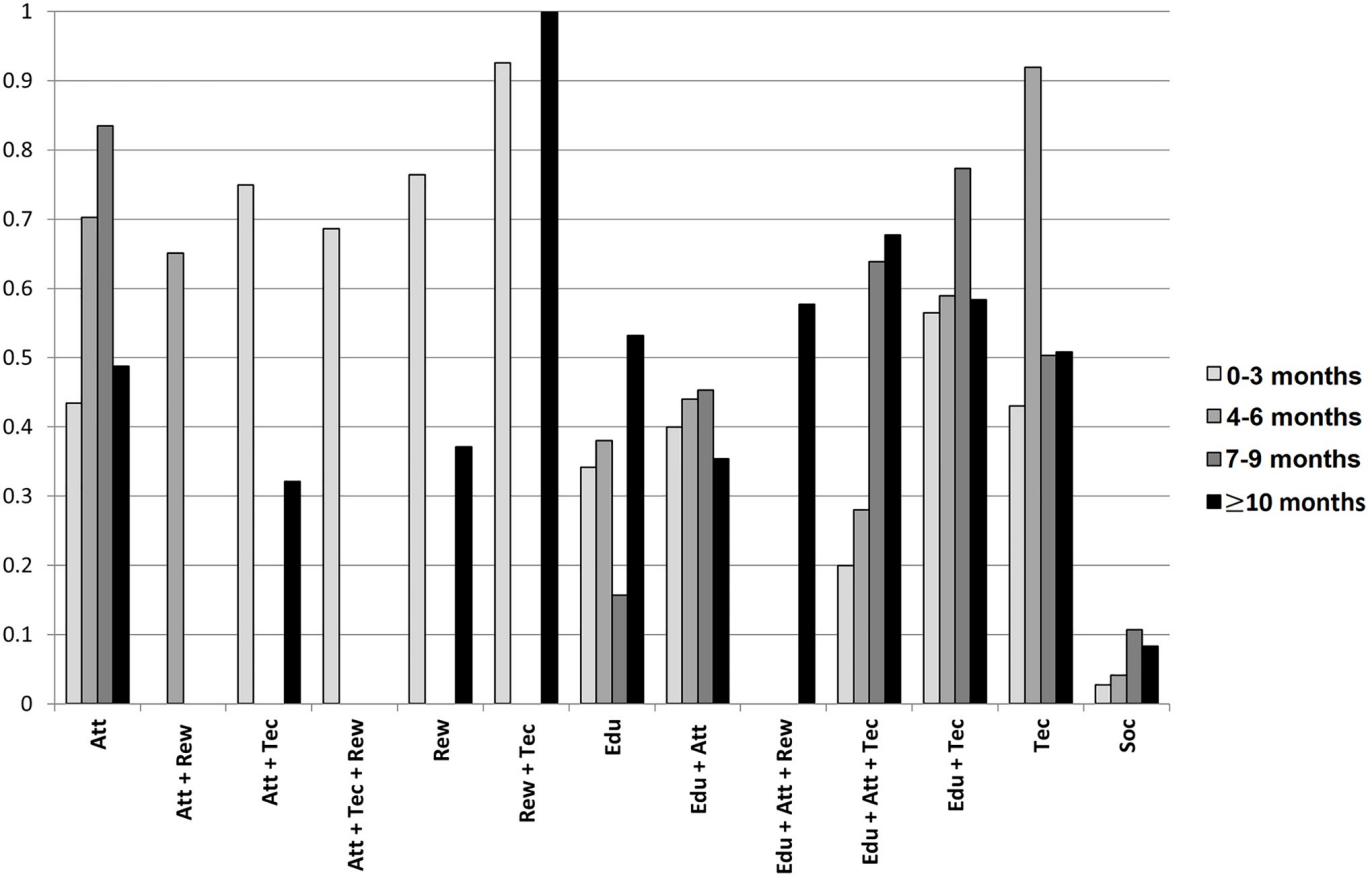
Summary of the effectiveness of the interventions over time considering the SUCRA analysis. SUCRA values can range from 0% (i.e. the intervention always ranks last) to 100% (i.e. the intervention always ranks first).

From the 16 built networks, accounting for both original and sensitivity analyses, 107 nodes were split during the evaluation of inconsistency (node-splitting analyses). Overall, results of direct and indirect evidence were consistent for all these networks (p-values > 0.050 in all cases), suggesting that conditions required for the analyses were met. Only 7 comparisons presented p-values close to the limit of significance (between 0.050 and 0.070). For complete results of node-splitting analyses are presented in S5 Table. The effect size of all the comparisons between interventions in each time period is presented in Table 1. The ranking probabilities of each intervention to be the best, second best and so on is expressed as SUCRA analysis (Fig 3 and S2 Fig).

**Table 1:** Consistency analyses of multiple comparison analyses for the overall composite measure in part A: 0–3 months (top right) and 4–6 months (top left) and part B: 7–9 months (bottom right) and ≥10 months (bottom left).

A											
Att + Rew	--	--	--	--	--	--	--	--	--	--	--
--	Att + Tec + Rew	1.28 (0.11, 13.70)	0.48 (0.07. 2.64)	2.94 (0.29, 26.39)	1.67 (0.08, 108.62)	0.38 (0.05, 2.26)	0.48 (0.07, 2.60)	0.57 (0.08, 3.24)	0.42 (0.06, 2.19)	0.25 (0.04, 1.31)	0.46 (0.07, 2.48)
--	--	Att + Tec	0.37 (0.08, 1.80)	2.27 (0.27, 19.61)	1.31 (0.07, 84.69)	0.28 (0.05, 1.62)	0.37 (0.07, 1.94)	0.44 (0.08, 2.48)	0.32 (0.06, 1.67)	0.19 (0.04, 1.01)	0.35 (0.06, 1.91)
0.90 (0.19, 4.46)	--	--	Att	**6.40 (1.60, 26.69)**	3.60 (0.30, 103.50)	0.72 (0.36, 1.43)	0.93 (0.53, 1.66)	1.23 (0.62, 2.34)	0.91 (0.56, 1.47)	**0.54 (0.35, 0.85)**	0.99 (0.60, 1.65)
--	--	--	--	Rew + Tec	0.57 (0.03, 20.93)	**0.11 (0.03, 0.47)**	**0.15 (0.04, 0.58)**	**0.19 (0.05, 0.79)**	**0.14 (0.04, 0.55)**	**0.09 (0.02, 0.32)**	**0.16 (0.04, 0.58)**
--	--	--	--	--	Rew	0.20 (0.01, 2.51)	0.26 (0.01, 3.18)	0.34 (0.01, 4.00)	0.26 (0.01, 3.00)	0.15 (0.01, 1.76)	0.28 (0.01, 3.28)
0.55 (0.11, 2.76)	--	--	0.61 (0.30, 1.25)	--	--	Edu + Att + Tec	1.30 (0.67, 2.57)	1.70 (0.81, 3.58)	1.27 (0.69, 2.33)	0.75 (0.43, 1.32)	1.38 (0.74, 2.55)
0.68 (0.14, 3.27)	--	--	0.75 (0.40, 1.38)	--	--	1.23 (0.65, 2.28)	Edu + Att	1.31 (0.71, 2.38)	0.97 (0.64, 1.48)	**0.58 (0.40, 0.83)**	1.06 (0.68, 1.64)
0.78 (0.17, 3.72)	--	--	0.85 (0.47, 1.57)	--	--	1.41 (0.77, 2.64)	1.14 (0.70, 1.88)	Edu + Tec	0.75 (0.43, 1.27)	**0.45 (0.27, 0.73)**	0.81 (0.45, 1.43)
0.65 (0.15, 2.94)	--	--	0.72 (0.42, 1.20)	--	--	1.18 (0.67, 2.08)	0.96 (0.62, 1.49)	0.84 (0.57, 1.22)	Edu	**0.60 (0.47, 0.76)**	1.09 (0.75, 1.55)
0.42 (0.09, 1.94)	--	--	**0.46 (0.28, 0.77)**	--	--	0.76 (0.46, 1.27)	**0.62 (0.43, 0.89)**	**0.54 (0.38, 0.76)**	**0.65 (0.51, 0.83)**	SOC	**1.82 (1.39, 2.39)**
1.24 (0.26, 5.81)	--	--	1.38 (0.77, 2.40)	--	--	**2.27 (1.25, 4.04)**	**1.83 (1.16, 2.92)**	**1.61 (1.03, 2.45)**	**1.92 (1.33, 2.75)**	**2.96 (2.22, 3.94)**	Tec
B											
Att + Tec	--	--	--	--	--	--	--	--	--	--	
1.29 (0.43, 3.74)	Att	--	--	--	0.74 (0.27, 1.96)	0.59 (0.22, 1.48)	0.89 (0.32, 2.31)	**0.40 (0.13, 0.98)**	**0.37 (0.17, 0.84)**	0.63 (0.22, 1.90)	
**24.10 (4.45, 135.58)**	**18.71 (4.48, 86.42)**	Rew + Tec	--	--	--	--	--	--	--	--	
1.07 (0.24, 4.54)	0.84 (0.26, 2.57)	**0.04 (0.01, 0.26)**	Rew	--	--	--	--	--	--	--	
1.62 (0.29, 8.94)	1.27 (0.30, 5.32)	**0.07 (0.01, 0.47)**	1.51 (0.26, 8.54)	Edu + Att + Rew	--	--	--	--	--	--	
1.60 (0.49, 5.09)	1.24 (0.61, 2.49)	**0.07 (0.01, 0.29)**	1.48 (0.44, 5.17)	0.97 (0.21, 4.39)	Edu + Att + Tec	0.80 (0.33, 1.80)	1.22 (0.49, 2.89)	0.55 (0.19, 1.33)	0.54 (0.24, 1.07)	0.86 (0.31, 2.54)	
1.12 (0.36, 3.42)	0.87 (0.49, 1.58)	**0.05 (0.01, 0.20)**	1.04 (0.33, 3.39)	0.69 (0.16, 3.05)	0.71 (0.33, 1.49)	Edu + Att	1.50 (0.71, 3.32)	0.70 (0.29, 1.43)	0.69 (0.38, 1.16)	1.09 (0.46, 2.95)	
1.39 (0.48, 3.93)	1.08 (0.69, 1.67)	**0.06 (0.01, 0.23)**	1.28 (0.44, 3.90)	0.85 (0.21, 3.56)	0.87 (0.46, 1.67)	1.23 (0.73, 2.10)	Edu + Tec	0.46 (0.17, 1.03)	**0.45 (0.23, 0.80)**	0.72 (0.28, 1.98)	
1.34 (0.46, 3.75)	1.04 (0.70, 1.54)	**0.06 (0.01, 0.22)**	1.24 (0.42, 3.73)	0.81 (0.20, 3.40)	0.84 (0.44, 1.60)	1.19 (0.72, 1.96)	0.97 (0.68, 1.35)	Edu	0.97 (0.54, 2.01)	1.56 (0.66, 4.90)	
0.78 (0.28, 2.14)	**0.61 (0.42, 0.89)**	**0.03 (0.01, 0.13)**	0.73 (0.26, 2.13)	0.48 (0.12, 1.96)	**0.49 (0.27, 0.88)**	0.70 (0.44, 1.11)	**0.56 (0.44, 0.72)**	**0.59 (0.45, 0.76)**	SOC	1.61 (0.82, 3.64)	
1.30 (0.47, 3.58)	1.02 (0.65, 1.61)	**0.05 (0.01, 0.21)**	1.22 (0.41, 3.69)	0.80 (0.19, 3.33)	0.82 (0.44, 1.56)	1.17 (0.68, 1.98)	0.94 (0.67, 1.32)	0.98 (0.69, 1.41)	**1.67 (1.31, 2.16)**	Tec	

Effect sizes are reported as OR (with 95% CrI). Comparisons are read from left to right (row to column above, column to row below) (e.g. the effect of Edu to SOC is 0.60 in 0–3 months). An OR <1 indicates a more effective intervention. Bold data comparisons are statistically significant. Edu: educational, Att: attitudinal, Tec: technical, Rew: rewards, SOC: standard of care.

### 0–3 month follow-ups

The 0–3 month network included data from 99 studies (n = 35,714 patients) and comprised 11 different nodes. Follow-up time varied from 0–3 months, with the most common period being 10–12 weeks (n = 25 studies), followed by 4–6 weeks (n = 22 studies).

### 4–6 month follow-ups

The 4–6 month network included data from 104 studies (n = 31,736 patients), compromising eight arms. Follow-up time varied from 4–6 months, with the majority studies using 24–26 weeks (n = 54).

### 7–9 month follow-ups

Data from 18 studies (n = 7,586 patients) were included in the 7–9 month network with standard of care and six interventions (attitudinal, educational, educational + attitudinal, educational + attitudinal + technical, educational + technical, technical). No studies reporting data on reward components were reported in the literature for this time period. Follow-up time varied from 7–9 months.

### ≥10 month follow-ups

Ninety-four studies were included in the ≥10 month network (n = 152,372 patients) comprising 11 arms. Follow-up time varied from 10–40 months, with 12 months (n = 60 studies) as the most common.

### Components of interventions

Across all time periods, multiple interventions were effective and standard of care ranked last in all SUCRA analyses (mean SUCRA value 6%). Single component interventions were found to be the most effective in follow-ups of 4–6 months and 7–9 months, with technical (SUCRA value 92%) (OR 0.33, [95% CrI 0.25–0.45] vs SOC) and attitudinal (SUCRA value 84%) (OR 0.37, [95% CrI 0.17–0.84] vs. SOC) ranking first in each time period respectively. The combination of educational + technical components consistently performed well, with an average SUCRA value around 63%, and was always more effective than educational components alone (highest SUCRA value 53% in ≥10 month follow-ups). The addition of an attitudinal component to educational + technical components past 10 months increased effectiveness (attitudinal + educational + technical OR 0.49, [95% CrI 0.27–0.88] vs SOC; educational + technical OR 0.56 [95% CrI 0.44–0.72] vs SOC). Rewards + technical was considered an effective intervention in the shortest time period (0–3 months; n = 1 study) and longest (≥10 months; n = 1) (92% and 100% in the SUCRA analysis respectively) and presented significant statistical differences compared to almost all interventions and standard of care (OR 0.03, [95% CrI 0.01–0.13] vs. SOC). However, conclusions cannot be determined on this combination based on the limited amount of evidence. Other combinations including reward components were also limited, appearing in only seven studies across all time periods with no statistically significant comparisons.

### Changes over time

To facilitate data interpretation, Fig 3 shows a summary of the changes in the position of rank order for each intervention over time (for final rank orders see S6 Table). Considering only the interventions reporting data on all four time periods, educational + attitudinal + technical presented increasing comparative effectiveness over time, with a final SUCRA value reaching around 68% (median 46%, interquartile range [IQR] 26.0%-64.8%). Attitudinal interventions also presented increasing values through time up until the 7–9 month follow-ups, though dropped in effectiveness ≥10 months without the addition of other components. Technical interventions presented consistent values during the time periods (around 50% probability), except for the 4–6 month time period. In follow-ups less than 10 months, educational had a mean 29% chance of being the best option, but this value increased to 53% past 10 months (median 36%, IQR 30.0%-42.0%). The effectiveness of the interventions educational + attitudinal and educational + technical were relatively stable during all time periods at around 40% (IQR 39.0%-44.3%) and 63% (IQR 58.0%-64.0%), respectively. For attitudinal + rewards, attitudinal + technical, attitudinal + technical + rewards, rewards and educational + attitudinal + rewards further extrapolation was not possible due to the lack of studies reporting data for all follow-up periods.

### Sensitivity analyses

Overall, studies’ sample sizes were found to have low influence on the comparative effectiveness of interventions. Analyses that included only studies with more than 30 patients presented equivalent results compared to the original analyses for all four time periods. Results from sensitivity analyses of articles published before 2007 or after 2007 showed that for follow-ups ≥10 months, differences in the position of the interventions in the rank order were observed compared to the original analyses. These, however, were similar to those obtained in the original analyses for shorter time periods. When evaluating studies published before 2007, the comparisons attitudinal vs. standard of care and educational + attitudinal + technical vs. standard of care lose their statistical significance with the enlargement of the 95% CrI (OR 0.81 [95% CrI 0.38–1.74] and OR 0.89 [95% CrI 0.19–4.13], respectively). By removing these studies from the original analyses and accounting for studies published after 2007, both interventions became statistically superior to standard of care (0.56 [0.35–0.89] and 0.44 [0.22–0.83], respectively) with final SUCRA values of 67% and 83%, respectively. No other significant differences were observed (S1 File).

## Discussion

By using NMAs to synthesise evidence from more than 200 studies on medication adherence, we found that time significantly influenced some interventions, while having no influence on others. We found a trend towards any intervention, either singly or in combination, being more effective than standard of care, although in many cases the trend did not reach statistical significance. This review demonstrated that multicomponent interventions including educational, attitudinal and technical aspects are more effective than single component interventions. This supports other research in adherence [11], pharmacology [35] and health care more broadly [36], and is logically reinforced by the idea that adherence is a multifaceted and complex issue [6, 14]. While other adherence research has shown this by indirect comparison to standard of care [20], our research has shown this by direct comparison, albeit through an estimate.

The comparative effectiveness of complex, multicomponent interventions is not surprising. But it raises the question of how to focus our efforts on the best combination of interventions. We found that an adherence intervention that included a technical component, either singly or in combination, showed benefits that were consistent across time, which builds on other research about effectiveness [19, 21]. A technical component, such as reminders and feedback from healthcare professionals, can be an effective and inexpensive opportunity to add to standard practice to improve adherence [37]. Reward components were found effective when present in the networks, especially when combined with technical components. However, due to the lack of studies and evidence of interventions including reward components, it is not reasonable to draw conclusions or recommendations.

This review also revealed the effectiveness of interventions with an attitudinal or educational component increased with time, but declined after 10 months when used alone. However, they continued to become more effective past 10 months when in combination with other components. A possible explanation is that while attitudinal change is important, its effect is difficult to sustain without other elements. Similar themes have been found in other areas of public health and psychology [36, 38]. Brehm’s motivational intensity theory states importance and difficulty of a goal determines motivation [39]. Educational intervention components may be necessary for patients to understand the importance of adherence while technical components can simplify the medication taking process. Thus, this allows motivation from attitudinal components to fully develop and be sustained.

Many studies have shown that adherence declines over time [16, 17, 21]. This research shows for the first time that the approach needed to support adherence may change over time. Adherence to medication should not be considered a fixed concept, as it is multi-dimensional in nature. The complexity of medication adherence behaviors are reflected in the adherence taxonomy proposed by the ABC (Ascertaining Barriers for Compliance) Project Team [14] and the five dimensions of medication adherence classified by WHO [40–42]. We have demonstrated in this review that interventions to improve adherence can have an impact that varies depending on the time at which they are used. With more than half of American adults having at least one chronic condition [43], and with many of these conditions requiring long-term management [44, 45], future efforts must be focused on interventions inducing adherence change that is sustained for long periods.

Our strengths of this study are found in our statistical approach used. Network meta-analysis creates more powerful and robust evidence compared to standard meta-analysis by using both direct and indirect evidence [23]. All the built networks demonstrated robustness with no significant inconsistency between direct and indirect comparisons found. This is also the first network of its kind to evaluate adherence across all clinical conditions in addition to looking at variations in effectiveness of interventions across time. By acknowledging that adherence is a multi-dimensional topic affected by multiple factors including therapy-related, condition-related, health system-related, socio-economic-related, and patient-related factors [7], we aimed to create a broader picture of the landscape of medication adherence. This was achieved by evaluating the patterns and changes of the effect of intervention components comprehensively across all clinical conditions over time, by not limiting our research to a clinical condition, a setting or a specific intervention type. Previous research demonstrated that the effectiveness of interventions may be related to the clinical condition [13]. Although including different clinical conditions in the network meta-analysis may be considered as a drawback due the potential heterogeneity induced, in our research, the four periods of evaluation contain an almost identical mix of medical conditions. Additionally, the heterogeneity between trials was below 70% in the vast majority of the comparisons, which is not unexpected when gathering evidence about complex interventions. Moreover, we evaluated intervention effects on all clinical conditions over time to account for the fact that determinants and issues of non-adherence are often comparable across medications and disease states. [11, 12]. While our networks were large and with many direct comparisons and a concern for heterogeneity, we can be confident in our networks due to no evidence of inconsistency being found in node-splitting analysis of direct and indirect evidence. Future research efforts should continue to expand on this landscape, including the effects of time on adherence as well as aiming to achieve the goal of long-term sustainability of improved adherence. Furthermore, sustainability of improved adherence first requires implementation of adherence enhancing interventions [46], a difficult process into the already overextended and resource-deficient practice of routine health care [45]. While multiple intervention components may be necessary for maintenance of adherence, too many components may overwhelm and produce a negative effect [47]. To support the thinking of policy-makers and healthcare professionals, we must determine where the best compromise in complex interventions lie for cost-effective and resource-limited approaches.

Our review has limitations. One is a lack of data across all combinations, with few trials available for some interventions and not all possible combinations of components being evaluated. Moreover, not all interventions or combinations were presented across all time periods, preventing a full narrative of temporal trends. The methodological quality of the included trials was mostly unclear, with a lack of complete outcome data or poor description of the study methodology definition of the evaluated interventions, and how they were delivered. Thus, only 53.2% of the studies could be part of the quantitative synthesis as they properly reported categorical results on patient’s adherence.

Additionally, only studies measuring implementation adherence were included, as initial review of the literature did not reveal enough studies reporting initiation and persistence adherence. To assist interpretability, the adherence-enhancing interventions were grouped into categories based on previous literature, but we acknowledge that a different approach of categorization may alter some results. Finally, while we decided to categorize a trial arm as standard of care if it was so determined as such by the individual study, we understand the definition of standard of care may vary by country or healthcare system.

## Conclusions

In conclusion, the results from this systematic review and network meta-analysis demonstrate several interventions including educational, attitudinal, technical and multicomponent strategies are effective in enhancing medication adherence. Multicomponent interventions incorporating educational, attitudinal, and technical aspects demonstrated greater sustainability of adherence over time. Technical interventions remained consistent in effectiveness across follow-up periods, while educational and attitudinal interventions were more effective with longer follow-up times, suggesting they may take more time to reach their potential in improving medication adherence. This research can be used to guide policy-makers and healthcare professionals in selecting effective multicomponent interventions, while future research should evaluate cost-effectiveness of these interventions.

## Supporting information

S1 Table Complete search strategy. (DOCX)

S2 Table Category definitions. (DOCX)

S3 Table Risk of bias summary. (PDF)

S4 Table Heterogeneity between trials comparisons for the composite measure. (DOCX)

S5 Table Node-splitting analyses. (DOCX)

S6 Table Final rank orders from SUCRA analyses. (DOCX)

S1 Appendix Complete references. a. Included meta-analysesb. Included primary studiesc. Excluded primary studiesd. Studies included in the network meta-analysis
(DOCX)

S1 Fig Risk of bias graph. (PDF)

S2 Fig SUCRA analyses. (DOCX)

S1 File Sensitivity and sub-group analyses. (DOCX)

S2 File PRISMA checklist. (DOC)

S3 File PRISMA NMA checklist. (DOCX)
