# Supplementary material for: Temporal effectiveness of interventions to improve medication adherence: A network meta-analysis
Source: PLoS One. 2019 Mar 12;14(3):e0213432. doi: 10.1371/journal.pone.0213432 (PMC6413898; doi:10.1371/journal.pone.0213432)
Supplement: S1 Table — (DOCX) [file pone.0213432.s001.docx]

**S1 Table. Complete search strategy and category definitions**

**Search strategy**

| **PubMed** | **#1** (“drug therapy”[Mesh Terms] OR “medication[Title/Abstract]) AND (“patient compliance”[Mesh Terms] OR “medication adherence”[Mesh Terms] OR “medication adherence”[Title/Abstract])  **#2** “systematic review”[Title/Abstract] OR “meta-analysis”[Publication type] OR “meta-analysis”[Title/Abstract]  **#1 AND #2** |
| --- | --- |
