# Supplementary material for: Temporal effectiveness of interventions to improve medication adherence: A network meta-analysis
Source: PLoS One. 2019 Mar 12;14(3):e0213432. doi: 10.1371/journal.pone.0213432 (PMC6413898; doi:10.1371/journal.pone.0213432)
Supplement: S2 Table — (DOCX) [file pone.0213432.s002.docx]

**S2 Table. Category definitions**

| **Category** | **Definition** |
| --- | --- |
| Educational | Every intervention where a professional provided any kind of knowledge (e.g. medication information, disease state information, importance of adherence information), in any form (e.g. written, oral, in group, by telephone), to a patient with the aim of modifying patient’s beliefs, attitudes or skills that facilitate adherence. |
| Attitudinal | Interventions aiming to modify behavioral intention (theory of planned behavior) based on modifying patient’s attitudes or subjective norm, delivered in any form (e.g. written, oral, in group, by telephone). |
| Technical | Interventions providing any gadget, instrument, or system that facilitate the medication intake, through reminders, regime simplifications, follow-ups, direction observation therapy, self-monitoring, cue-dose training, feedback etc. |
| Rewards | Interventions that produce awards (or penalties) associated to a better (or worst) medication adherence. |
