# Supplementary material for: Temporal effectiveness of interventions to improve medication adherence: A network meta-analysis
Source: PLoS One. 2019 Mar 12;14(3):e0213432. doi: 10.1371/journal.pone.0213432 (PMC6413898; doi:10.1371/journal.pone.0213432)
Supplement: S3 Table — (PDF) [file pone.0213432.s003.pdf]

	Random sequence generation (selection bias)	Allocation concealment (selection bias)	Blinding of participants and personnel (performance bias)	Blinding of outcome assessment (detection bias)	Incomplete outcome data (attrition bias)	Selective reporting (reporting bias)	Other bias
Adeyemo, 2013	?	?	–	+	–	+	+
Aguwa, 2008	–	?	?	–	–	+	?
Alhalaiqa, 2012	+	+	?	+	?	+	+
Aljumah, 2015	+	?	–	+	+	+	+
Al-Saffar, 2005	?	?	+	+	+	+	?
Altice, 2007	–	?	?	?	+	+	+
Amado, 2011	?	?	?	+	+	+	+
Anderson, 2010	?	?	?	+	+	+	?
Andrade, 2005	?	?	?	?	+	+	+
Andrejak, 2000	?	?	–	+	?	+	?
Antoni, 2006	+	?	?	+	–	+	?
Antonicelli, 2010	?	?	?	–	?	+	+
Apter, 2011	+	?	?	+	+	+	+
Armour, 2007	+	?	?	?	+	+	+
Arora, 2014	+	?	?	+	–	+	–
Asplund, 1984	?	?	?	+	?	+	?
Azrin, 1998	?	?	?	+	?	+	?
Babamoto, 2009	+	?	?	+	–	+	?
Bailey, 1990	+	+	–	+	+	+	?
Bailey, 1999	?	?	?	+	+	+	?
Baird, 1984	?	?	?	+	?	?	?
Ball, 2006	+	?	?	?	–	?	?

Basso, 2013	+	?	?	+	+	+	?
Begley, 1997	?	?	?	+	?	+	?
Bender, 2010	+	?	?	+	?	+	+
Berg, 1997	?	?	?	+	?	+	?
Berg, 2011	−	?	?	+	+	+	?
Berger, 2008	−	+	?	+	−	+	?
Bessa, 2016	+	+	?	+	+	+	+
Beune, 2014	+	−	−	+	+	+	+
Bisharat, 2012	?	?	?	+	+	+	+
Blenkinsopp, 2000	?	?	?	−	−	+	?
Bobrow, 2016	+	+	−	+	+	+	+
Bogner, 2008	?	?	?	+	?	+	+
Bogner, 2010	?	?	?	+	+	+	?
Bogner, 2012	+	?	−	+	+	+	+
Boissel, 1996	+	?	−	−	?	+	?
Bond, 2007	+	?	−	+	−	+	+
Boswort, 2008	?	?	?	+	+	+	?
Bouvy, 2003	+	?	?	+	−	+	?
Bove, 2013	+	?	?	−	?	+	?
Boyle, 2008	?	?	?	+	−	+	?
Brankin, 2006	−	?	?	+	?	+	?
Broekhuizen, 2012	+	?	−	+	?	+	+
Brook, 2005	?	?	−	+	−	+	?
Brown, 1997	?	?	−	+	+	+	?
Brown, 2009	+	?	?	+	?	+	?
Burrelle, 1987	?	?	?	+	+	?	?
Calvert, 2012	+	+	?	+	?	+	+
Capoccia, 2004	?	?	?	+	+	+	?
Carrico, 2006	?	?	+	+	+	+	?
Carter, 2010	?	?	?	+	−	+	+
Castellano, 2014	+	+	?	+	+	+	+
Castle, 2007	+	?	?	+	+	+	?
Chaisson, 2001	+	?	−	+	−	+	?
Chan, 2010	?	?	−	?	−	+	?

[illegible]	?	?	−	?	−	+	?
Chan, 2011	+	+	−	?	+	+	+
Chang, 2010	+	−	−	+	+	+	+
Charles, 2007	+	+	+	+	?	+	?
Chatkin, 2006	?	?	?	+	+	+	?
Cheung, 1988	?	?	?	+	?	+	?
Chien, 2015	?	?	−	+	+	+	+
Chisholm, 2001	?	?	?	+	+	+	?
Choudhry, 2011	+	?	?	+	+	+	+
Cizmic, 2015	+	?	?	+	+	+	+
Claborn, 2014	+	?	?	+	−	+	?
Clowes, 2004	+	+	?	+	+	+	?
Cochran, 2984	?	?	−	+	+	+	?
Cole, 1971	?	?	?	+	?	+	?
Collier, 2005	?	?	−	+	+	+	+
Cordasco, 2009	?	?	?	+	−	+	+
Cossette, 2012	+	+	−	+	+	+	+
Costa, 2008	+	?	−	−	?	+	?
Cramer, 1995	?	?	−	+	?	+	+
Cramer, 1999	?	?	?	+	−	+	?
Cramer, 2005	−	?	?	+	?	+	−
Cramer, 2006	−	?	?	+	?	+	?
Crockett, 2006	?	?	?	?	?	+	?
Crome, 1982	?	?	?	+	+	+	?
D'Souza, 2010	?	?	?	+	−	+	+
Da Costa, 2012	+	+	?	?	−	+	+
de Bruin, 2010	+	?	?	+	+	+	?
Delmas, 2007	?	?	?	+	+	+	−
Derose, 2013	+	+	?	−	+	+	−
Detry, 1995	−	?	−	+	−	+	?
Dilorio, 2003	+	?	?	+	+	+	?
Dilorio, 2008	+	?	?	+	+	+	?
Dilorio, 2009	?	?	?	+	+	+	?
Dogan, 2003	?	?	?	?	?	?	?

Du, 2016	+	?	?	?	+	+	+
Dusing, 2009	?	?	−	+	+	+	+
Edworthy, 1999	?	?	?	−	+	+	?
Edworthy, 2007	+	?	?	−	+	+	?
Eker, 2012	?	?	?	+	+	+	+
Elixhauser, 1990	?	?	?	+	−	+	?
Elkjaer, 2010	+	+	?	+	+	+	−
Erickson, 2003	?	?	?	+	?	+	+
Eron, 2000	?	?	−	?	+	+	?
Eron, 2004	?	?	?	+	+	+	−
Eshelman, 1976	?	?	?	+	−	+	?
Eussen, 2010	+	?	−	+	+	+	+
Evans, 2010	+	+	−	+	+	+	?
Evans-Hudnall, 2014	+	?	?	−	+	+	?
Falces, 2008	+	?	?	+	?	+	?
Farmer, 1994	?	?	?	+	?	+	?
Farmer, 2012	+	?	?	+	?	+	+
Faulkner, 2000	+	?	?	+	+	+	?
Fernandez, 2008	?	?	?	+	?	+	+
Finley, 2002	−	?	?	+	−	+	?
Finley, 2003	?	?	−	+	?	+	?
Fisher, 2011	+	?	−	+	−	?	?
Foster, 2014	+	?	−	+	+	+	−
Friedberg, 2015	+	+	−	+	+	+	+
Friedman, 1996	+	?	?	+	?	+	?
Fulmer, 1999	+	?	?	+	?	+	?
Fyllingen, 1991	?	+	−	+	+	+	?
Gabriel, 1977	?	?	?	+	?	+	?
Gallant, 2006	+	?	?	+	+	+	+
Gamble, 2011	?	?	?	+	+	+	+
Garcia, 2015	+	+	?	+	+	+	+
Garcia-Cardenas, 2013	+	?	−	?	+	+	+
Garnett, 1981	+	?	?	+	?	+	?
[illegible]	[illegible]	[illegible]	[illegible]	[illegible]	[illegible]	[illegible]	[illegible]

Gatwood, 2016	+	?	?	+	−	+	?
Gazmararian, 2010	?	?	?	+	?	+	+
Geiter, 1987	?	?	−	+	?	+	?
Girvin, 1999	?	?	−	+	?	+	?
Glanz, 2012	+	?	−	+	?	+	?
Goggin, 2013	+	?	?	+	+	?	+
Golin, 2006	?	?	?	+	−	+	?
Gonzalez-Fernandez, 1990	?	?	?	+	?	+	?
Goodyer, 1995	?	?	?	+	?	+	?
Goswami, 2013	+	?	−	+	−	+	−
Goujard, 2003	?	?	?	+	?	+	?
Granger, 2015	?	?	?	+	+	+	?
Grant, 2003	?	?	?	−	−	+	?
Gray, 2006	+	?	−	+	+	+	+
Green, 2014	+	?	?	+	?	+	?
Greer, 2014	−	?	?	+	?	+	+
Gross, 2009	−	+	−	+	+	+	+
Gross, 2013	+	+	−	+	−	+	+
Gross, 2015	−	+	−	+	+	?	+
Grymonpre, 2001	+	?	?	+	+	+	?
Guirado, 2011	?	?	?	+	−	+	+
Gujral, 2014	+	?	?	+	−	+	+
Guthrie, 2007	?	?	−	−	−	+	?
Gwady-Sridhar, 2005	+	?	−	+	+	+	?
Hacihasanoglu, 2011	+	−	?	+	?	+	+
Hadji, 2013	?	?	−	?	−	+	−
Hardy, 2011	+	?	?	+	+	+	?
Hawkins, 1979	?	?	?	+	−	+	?
Haynes, 1976	+	?	?	+	+	+	?
Heisler, 2010	+	+	−	−	?	+	+
Henry, 1999	?	?	−	+	?	+	?
Hersch, 2013	−	?	?	+	+	+	?
Hilleman, 1993	−	?	−	+	?	?	?
Hirsch, 2009	−	?	?	+	?	+	+

[illegible]	−	?	?	+	?	+	+
Hirsch, 2011	−	−	?	+	?	+	+
Ho, 2008	?	?	?	+	?	+	+
Ho, 2014	+	+	?	+	+	+	+
Holstad, 2011	?	?	?	+	+	+	?
Holstad, 2012	+	?	?	+	−	+	?
Holzemer, 2006	?	?	?	+	−	+	?
Homer, 2009	+	+	?	+	+	+	?
Hornnes, 2011	+	?	−	?	?	+	?
Horvath, 2013	+	?	?	+	−	+	?
Hosseininasab, 2014	+	?	?	+	+	+	+
Hunt, 2008	+	?	−	+	−	+	+
Ingersoll, 2011	?	?	−	+	−	+	?
Interian, 2013	+	?	?	+	+	?	?
Jalal, 2016	?	+	−	+	+	+	?
Janson, 2003	?	?	?	+	+	+	?
Jarab, 2012	+	?	?	+	+	+	+
Jerant, 2003	+	+	?	?	+	+	?
Jiang, 2007	+	?	?	−	+	+	?
Johnson, 1978	?	?	?	+	+	+	?
Johnson, 2007	+	?	?	+	−	?	?
Johnson, 2011	+	?	?	+	+	+	+
Jones, 2003	?	?	−	+	?	+	?
Joost, 2014	?	?	?	+	+	+	+
Kalichman, 2011	+	?	?	+	+	+	?
Kamal, 2015	?	+	−	+	+	+	+
Kardas, 2004	?	?	−	+	+	+	?
Kardas, 2007	?	?	?	+	+	+	+
Kardas, 2012	?	?	−	+	?	+	?
Katon, 1996	+	?	?	+	−	+	?
Kauric-Klein, 2012	+	?	?	+	?	+	+
Kellaway, 1979	−	?	?	−	?	+	?
Kelly, 1990	?	?	?	−	−	+	?
Kertes, 2008	−	?	?	+	?	+	?

Khonsari, 2015	?	?	−	+	+	+	+
Kim, 2008	−	?	?	+	?	+	?
Kim, 2013	+	?	−	−	+	+	?
Kim, 2014	?	?	?	+	?	+	+
Kiweewa, 2013	+	+	−	+	+	+	+
Klang, 2015	−	?	−	?	?	−	−
Klein, 2006	?	?	?	+	?	+	+
Klein, 2009	?	?	−	+	+	+	+
Koenig, 2008	+	−	?	+	+	+	?
Kogos, 2004	−	?	?	+	+	+	?
Konkle-Parker, 2012	?	?	?	+	−	+	?
Konkle-Parker, 2014	+	?	?	+	−	+	+
Kooy, 2013	+	?	?	+	+	+	?
Kopelowicz, 2003	?	?	?	+	?	+	?
Kotowycz, 2010	+	+	−	+	−	+	?
Kozuki, 2006	?	?	?	+	+	+	?
Krier, 1999	?	?	−	?	−	+	?
Kripalani, 2012	+	+	?	+	+	+	+
Kronish, 2012	?	?	?	+	?	+	?
Kronish, 2014	+	+	?	+	+	+	+
Kruse, 1991	?	?	?	+	?	+	?
Kubota, 2006	?	?	+	+	+	+	?
Kurth, 2014	?	?	?	?	+	+	−
Lee, 1996	?	?	+	+	−	+	?
Lee, 2006	+	+	−	+	+	+	−
Leenen, 1997	+	?	−	+	?	+	?
Lester, 2010	+	+	?	+	+	+	+
Levin, 2006	+	?	?	−	+	+	+
Levine, 1979	−	?	?	−	?	+	?
Lipton, 1994	+	?	?	−	?	+	?
Logan, 1982	?	?	?	+	?	+	?
Lourens, 1994	+	?	?	+	−	+	?
Lua and Neni, 2013	+	?	?	−	?	+	?
[illegible]	[illegible]	[illegible]	[illegible]	[illegible]	[illegible]	[illegible]	[illegible]

Lucas, 2013	+	+	?	+	+	+	?
Lv, 2012	?	?	?	−	−	?	+
Ma, 2014	+	?	−	+	+	+	?
Maduka and Tobin-West, 2013	+	+	?	−	+	+	+
Magid, 2011	+	?	?	+	+	+	+
Margolin, 2003	?	?	?	?	−	+	?
Markopoulous, 2015	+	?	?	?	−	+	−
Marquez Contreras, 2004	+	?	−	+	−	+	?
Marquez-Contreras, 2005	?	?	−	+	?	+	+
Matsumura, 2012	−	?	?	+	+	+	?
Mbuagbaw, 2012	+	+	?	+	+	+	?
McGillicuddy, 2013	?	?	?	+	+	+	+
McKenney, 1973	−	?	?	+	?	+	?
McKenney, 1978	+	?	?	+	?	+	?
McKenney, 1992	?	?	−	+	?	+	?
McKinstry, 2013	+	+	−	+	+	+	+
Mehos, 2000	+	?	?	+	?	+	?
Mehuys, 2008	+	+	?	+	−	+	?
Miller, 1990	?	?	?	−	?	+	?
Mols, 2015	?	?	?	−	+	+	+
Montes, 2011	+	+	−	+	+	+	−
Montori, 2011	+	?	?	+	−	+	+
Mooney, 2005	?	?	−	?	−	+	?
Mooney, 2007	?	?	−	+	−	+	?
Moore, 2013	−	?	?	+	?	+	+
Morgado, 2011	+	+	−	+	+	+	+
Morisky, 1985	+	?	?	+	?	+	?
Moss, 2010	+	?	?	+	?	+	+
Mugusi, 2009	−	?	?	−	+	+	?
Muir, 2012	?	?	?	+	+	+	+
Munoz, 2009	−	?	−	+	−	+	?
Murray, 1993	?	?	−	+	?	+	?
Nachega, 2010	?	+	?	+	−	?	?
Najafi, 2016	+	?	−	?	+	+	+

[illegible]	+	?	−	?	+	+	+
Nance, 2017	+	?	?	?	+	+	?
Nessman, 1980	?	?	?	+	?	+	?
Nielson, 2010	?	?	?	?	?	+	?
Nieuwkerk, 2012	+	?	−	+	+	+	?
Nollen, 2011	+	+	?	+	?	+	−
O'Connor, 2014	?	?	?	+	+	+	+
Ogedegbe, 2012	?	+	−	+	?	+	+
Ogedegbe, 2014	?	?	?	+	−	+	+
Oliver, 2011	?	?	?	+	−	+	?
Ollivier, 2009	+	?	?	+	?	+	+
Onyirimba, 2003	?	?	−	+	−	+	?
Pagoto, 2013	?	?	?	+	+	+	?
Park, 1996	?	?	−	+	?	+	?
Park, 2013	−	?	?	+	+	+	+
Park, 2014	+	?	−	+	−	+	+
Pearson, 2007	+	+	−	+	−	+	?
Peltzer, 2012	+	?	?	−	+	+	?
Piette, 2000	+	?	−	−	+	+	?
Piette, 2001	+	+	?	−	+	+	?
Pladevall, 2010	+	+	?	+	+	+	+
Pladevall, 2015	+	?	−	+	−	+	+
Planas, 2003	+	?	?	+	−	+	−
Polsook, 2008	+	?	?	?	+	+	?
Pop-Eleches, 2011	+	?	?	+	+	+	?
Powell, 1995	−	?	?	+	?	+	?
Pullar, 1988	?	?	?	+	+	+	?
Purcell, 2007	?	?	?	?	+	+	+
Pyne, 2011	+	?	−	+	−	+	+
Qureshi, 2007	+	?	+	+	+	+	+
Rabenda, 2008a	−	?	?	+	?	+	?
Rabenda, 2008b	−	?	?	+	?	+	+
Rathbun, 2005	+	?	?	+	−	+	?
Rawlings, 2003	?	?	−	+	−	+	?

Raynor, 1993	?	?	?	+	+	+	?
Rehder, 1980	?	?	?	+	−	+	?
Reynolds, 2008	?	?	?	+	−	+	−
Rich, 1995	+	?	?	+	?	+	?
Rickles, 2005	+	?	−	+	+	+	+
Rinfret, 2009	+	?	−	+	−	+	+
Robbins, 2013	+	?	?	+	+	+	+
Robinson, 2010	−	?	?	+	−	+	?
Roden, 1985	?	?	?	+	−	+	?
Rotheram-Borus, 2004	?	?	?	+	+	+	?
Rozenfeld, 1999	?	?	−	+	+	+	?
Rubio-Valera, 2013	+	?	−	+	+	+	+
Rudd, 2004	+	?	?	+	+	+	?
Ruiz, 2010	?	+	?	+	+	+	?
Sabin, 2010	+	?	?	+	?	+	?
Sackett, 1975	?	?	?	+	?	+	?
Safren, 2009	?	?	?	+	?	+	?
Safren, 2012	−	+	?	+	?	+	+
Saini, 2008	−	?	?	?	+	+	?
Saleem, 2013	+	?	−	−	+	+	+
Sampaio, 2008	?	?	?	+	−	+	?
Saunders, 1991	?	?	?	+	?	+	?
Schaffer and Tian, 2004	+	?	?	+	+	+	?
Schectman, 1994	?	?	−	+	+	+	?
Schmitz, 2005	?	?	+	+	−	+	?
Schneider, 2008	+	?	?	+	?	+	+
Schroeder, 2005	+	?	−	+	+	+	+
Sclar, 1991	?	?	?	+	?	+	?
Selke, 2010	−	+	?	?	+	+	?
Sewerynek, 2013	−	?	?	+	?	+	?
Sherrard, 2009	?	+	?	?	−	+	?
Shet, 2014	+	+	−	+	+	+	+
Shu, 2009	?	?	?	+	?	+	−
[illegible]	[illegible]	[illegible]	[illegible]	[illegible]	[illegible]	[illegible]	[illegible]

Silveira, 2014	+	?	−	?	?	+	?
Simkins, 1986	+	?	?	+	?	+	?
Simoni, 2007	+	+	−	+	+	+	?
Simoni, 2013	+	+	?	+	?	+	+
Sit, 2007	−	?	?	+	−	+	+
Skaer, 1993	?	?	?	+	?	+	?
Smith, 2007	?	?	?	?	?	+	?
Smith, 2008	+	?	?	+	+	+	+
Solomon, 1998	+	?	−	+	?	+	?
Sookaneknun, 2004	+	?	?	+	+	+	?
Stacy, 2009	+	?	?	+	?	+	+
Stewart, 2014	?	+	−	+	−	+	?
Stewart, 2014a	?	?	?	+	?	+	+
Strandbygaard, 2010	+	?	?	+	?	+	+
Su and Pergn, 2002	?	?	?	?	−	+	?
Svarstad, 2013	+	?	?	+	+	+	+
Taggart, 1981	?	?	?	+	−	+	?
Taiwo, 2010	+	?	?	+	?	+	?
Tan, 2010	−	?	?	+	?	+	−
Thom, 2013	+	?	−	+	+	+	−
Tinsel, 2014	?	?	−	+	?	+	?
Uysal, 2015	?	?	?	?	+	+	?
Valencia, 2008	?	?	−	+	?	+	+
van Onzenoort, 2010	?	?	+	+	?	+	+
van Servellen, 2003	+	?	?	−	+	+	?
Velligan, 2008	?	?	−	+	+	+	?
Vervloet, 2012	?	?	?	+	+	?	+
Vollmer, 2011	?	?	?	+	+	+	+
Vollmer, 2014	+	?	−	+	+	+	+
Vrijens, 2006	?	?	−	+	?	+	+
Wagner, 2013	?	?	?	+	+	+	?
Wakefield, 2012	?	?	?	+	?	+	+
Wald, 2014	+	?	?	−	?	+	?
Walker, 2000	?	?	?	+	?	?	?

[illegible]	[illegible]	[illegible]	[illegible]	[illegible]	[illegible]	[illegible]	[illegible]
Wan, 2016	+	+	+	+	?	+	?
Wang, 2010	+	?	?	?	+	+	+
Wang, 2011	+	?	?	+	+	+	+
Wang, 2014	+	?	?	–	–	+	+
Webb, 1980	?	?	?	+	?	+	?
Weinberger, 1991	?	?	?	+	?	+	?
Williams, 2014	–	?	?	+	?	+	+
Windsor, 1990	?	?	?	+	?	+	?
Winland-Brown, 2000	?	?	?	–	?	+	?
Wohl, 2006	+	+	?	+	–	+	–
Wong, 2013	+	?	?	+	+	+	+
Wong, 2017	+	?	?	?	+	+	?
Wyatt, 2004	?	?	?	–	–	+	?
Xavier, 2016	+	?	–	?	+	+	+
Young, 2012	+	?	?	+	?	+	+
Zang, 2010	?	?	?	+	+	+	+
Zarnke, 1997	+	?	–	–	+	+	?
Ziller, 2013	?	?	–	+	+	+	–
