# Supplementary material for: Temporal effectiveness of interventions to improve medication adherence: A network meta-analysis
Source: PLoS One. 2019 Mar 12;14(3):e0213432. doi: 10.1371/journal.pone.0213432 (PMC6413898; doi:10.1371/journal.pone.0213432)
Supplement: S4 Table — (DOCX) [file pone.0213432.s004.docx]

**S4 Table. Heterogenity between trials comparisons for the composite measure.**

| **1st period – Composite measure** | | |
| --- | --- | --- |
| **Direct comparison** | **Studies** | **I^2^** |
| Attitudinal + Technical x Attitudinal | 1 | - |
| Attitudinal x Educational | 3 | 0% |
| Attitudinal x Educational + Attitudinal + Technical | 1 | - |
| Attitudinal x Standard care | 7 | 0% |
| Educational x Educational + Technical | 2 | 33.0% |
| Educational x Educational + Attitudinal | 2 | 11.2% |
| Educational x Technical | 1 | - |
| Educational x Standard care | 30 | 54.5% |
| Educational + Attitudinal x Standard care | 13 | 69.1% |
| Technical x Rewards + Technical | 1 | - |
| Technical x Attitudinal + Technical + Rewards | 1 | - |
| Technical x Standard care | 31 | 37.1% |
| Rewards + Technical x Standard care | 1 | - |
| Rewards x Standard care | 1 | - |
| Educational + Attitudinal + Technical x Standard care | 6 | 4.4% |
| Educational + Technical x Standard care | 7 | 60.3% |
| **2nd period – Composite measure** | | |
| **Direct comparison** | **Studies** | **I^2^** |
| Attitudinal x Educational | 4 | 74.5% |
| Attitudinal x Standard care | 7 | 2.2% |
| Educational x Attitudinal + Rewards | 1 | - |
| Educational x Educational + Technical | 6 | 45.3% |
| Educational x Technical | 5 | 17.0% |
| Educational x Educational + Attitudinal | 1 | - |
| Educational x Standard care | 31 | 67.1% |
| Educational + Technical x Standard care | 16 | 54.2% |
| Educational + Technical x Technical | 1 | - |
| Educational + Attitudinal x Standard care | 16 | 61.3% |
| Technical x Standard care | 24 | 81.6% |
| Educational + Attitudinal + Technical x Standard care | 7 | 39.0% |
| **3rd period – Composite measure** | | |
| **Direct comparison** | **Studies** | **I^2^** |
| Attitudinal x Educational + Attitudinal + Technical | 1 | - |
| Attitudinal x Standard care | 3 | 42.8% |
| Educational + Attitudinal + Technical x Educational + Technical | 1 | - |
| Educational + Attitudinal + Technical x Educational + Attitudinal | 1 | - |
| Educational + Attitudinal + Technical x Standard care | 3 | 13.0% |
| Educational + Technical x Standard care | 5 | 64.5% |
| Educational + Technical x Educational + Attitudinal | 1 | - |
| Educational + Attitudinal x Educational | 1 | - |
| Educational + Attitudinal x Standard care | 4 | 65.9% |
| Educational x Standard care | 2 | 0% |
| Technical x Standard care | 3 | 0% |
| **4th period – Composite measure** | | |
| **Direct comparison** | **Studies** | **I^2^** |
| Rewards x Standard care | 1 | - |
| Attitudinal + Technical x Technical | 1 | - |
| Attitudinal + Technical x Standard care | 1 | - |
| Educational + Attitudinal + Technical x Standard care | 4 | 58.1% |
| Educational + Attitudinal + Rewards x Standard care | 1 | - |
| Attitudinal x Educational | 4 | 10.3% |
| Attitudinal x Standard care | 8 | 67.1% |
| Educational + Attitudinal x Educational | 2 | - |
| Educational + Attitudinal x Standard care | 7 | 72.4% |
| Educational x Educational + Technical | 1 | - |
| Educational x Technical | 2 | 0% |
| Educational x Standard care | 20 | 89.7% |
| Educational + Technical x Technical | 2 | - |
| Educational + Technical x Standard care | 26 | 87.9% |
| Technical x Standard care | 22 | 91.8% |
| Rewards + Technical x Technical | 1 | - |
