# Supplementary material for: Temporal effectiveness of interventions to improve medication adherence: A network meta-analysis
Source: PLoS One. 2019 Mar 12;14(3):e0213432. doi: 10.1371/journal.pone.0213432 (PMC6413898; doi:10.1371/journal.pone.0213432)
Supplement: S5 Table — (DOCX) [file pone.0213432.s005.docx]

**S5 Table. Node-splitting analyses**

**0-3 months**

| **Name** | **Direct Effect** | **Indirect Effect** | **Overall** | **P-Value** |
| --- | --- | --- | --- | --- |
| Attitudinal 1st , Educational 1st | -0,33 (-1,25, 0,56) | 0,00 (-0,57, 0,58) | -0,09 (-0,57, 0,38) | 0.53 |
| Attitudinal 1st , Educational + Attitudinal + Technical 1st | -0,46 (-2,04, 1,10) | -0,32 (-1,07, 0,42) | -0,33 (-1,03, 0,36) | 0.87 |
| Attitudinal 1st , Standard care 1st | -0,53 (-1,03, -0,02) | -0,82 (-1,66, -0,02) | -0,61 (-1,05, -0,16) | 0.53 |
| Educational 1st, Educational + Attitudinal 1st | 0,35 (-0,77, 1,51) | -0,03 (-0,48, 0,45) | 0,03 (-0,39, 0,44) | 0.55 |
| Educational 1st, Educational + Technical 1st | 0,96 (-0,03, 1,94) | 0,00 (-0,64, 0,63) | 0,29 (-0,24, 0,84) | 0.1 |
| Educational 1st, Standard care 1st | -0,58 (-0,87, -0,29) | 0,04 (-0,59, 0,66) | -0,48 (-0,75, -0,20) | 0.06 |
| Educational 1st, Technical 1st | 0,96 (-0,49, 2,48) | 0,05 (-0,33, 0,42) | 0,08 (-0,28, 0,44) | 0.23 |
| Educational + Attitudinal 1st, Standard care 1st | -0,50 (-0,89, -0,13) | -0,88 (-2,08, 0,32) | -0,54 (-0,91, -0,19) | 0.56 |
| Educational + Technical 1st, Standard care 1st | -0,66 (-1,23, -0,13) | -1,37 (-2,35, -0,39) | -0,81 (-1,32, -0,32) | 0.21 |

**4-6 months**

| **Name** | **Direct Effect** | **Indirect Effect** | **Overall** | **P-Value** |
| --- | --- | --- | --- | --- |
| Attitudinal 2nd, Educational 2nd | -0,63 (-1,47, 0,20) | -0,14 (-0,82, 0,53) | -0,33 (-0,86, 0,19) | 0.37 |
| Attitudinal 2nd, Standard care 2nd | -0,60 (-1,23, 0,02) | -1,11 (-1,97, -0,26) | -0,77 (-1,28, -0,27) | 0.35 |
| Educational 2nd, Educational + Attitudinal 2nd | 0,87 (-0,99, 2,94) | 0,06 (-0,39, 0,51) | 0,09 (-0,34, 0,53) | 0.59 |
| Educational 2nd, Educational + Technical 2nd | 0,61 (0,01, 1,24) | -0,10 (-0,59, 0,39) | 0,18 (-0,20, 0,56) | 0.07 |
| Educational 2nd, Standard care 2nd | -0,54 (-0,81, -0,28) | 0,00 (-0,47, 0,49) | -0,43 (-0,68, -0,18) | 0.06 |
| Educational 2nd, Technical 2nd | 0,88 (0,20, 1,57) | 0,59 (0,18, 1,00) | 0,65 (0,28, 1,01) | 0.45 |
| Educational + Attitudinal 2nd, Standard care 2nd | -0,47 (-0,86, -0,09) | -1,26 (-3,33, 0,61) | -0,50 (-0,87, -0,11) | 0.5 |
| Educational + Technical 2nd, Standard care 2nd | -0,54 (-0,94, -0,15) | -0,92 (-1,55, -0,28) | -0,61 (-0,96, -0,27) | 0.3 |
| Educational + Technical 2nd, Technical 2nd | 0,48 (-0,71, 1,73) | 0,44 (-0,03, 0,90) | 0,47 (0,03, 0,90) | 0.94 |

**7-9 months**

| **Name** | **Direct Effect** | **Indirect Effect** | **Overall** | **P-Value** |
| --- | --- | --- | --- | --- |
| Attitudinal 3rd, Educational + Attitudinal + Technical 3rd | -0,17 (-1,68, 1,31) | -0,47 (-1,72, 0,69) | -0,31 (-1,31, 0,67) | 0.73 |
| Educational 3rd, Educational + Attitudinal 3rd | 1,02 (-0,39, 2,45) | 0,17 (-0,83, 1,07) | 0,35 (-0,36, 1,24) | 0.26 |
| Educational 3rd, Standard care 3rd | -0,14 (-0,89, 0,62) | 0,75 (-0,75, 2,26) | -0,03 (-0,62, 0,70) | 0.25 |
| Educational + Attitudinal 3rd, Educational + Attitudinal + Technical 3rd | 0,17 (-1,04, 1,51) | 0,48 (-0,68, 1,73) | 0,23 (-0,59, 1,11) | 0.68 |
| Educational + Attitudinal 3rd, Educational + Technical 3rd | 0,56 (-0,70, 1,87) | 0,34 (-0,54, 1,37) | 0,41 (-0,34, 1,20) | 0.74 |
| Educational + Attitudinal 3rd, Standard care 3rd | -0,27 (-0,88, 0,38) | -0,70 (-1,73, 0,29) | -0,37 (-0,96, 0,14) | 0.42 |
| Educational + Attitudinal + Technical 3rd, Educational + Technical 3rd | 0,30 (-0,99, 1,68) | 0,10 (-1,02, 1,25) | 0,20 (-0,72, 1,06) | 0.78 |

**≥10 months**

| **Name** | **Direct Effect** | **Indirect Effect** | **Overall** | **P-Value** |
| --- | --- | --- | --- | --- |
| Attitudinal 4th, Educational 4th | -0,29 (-0,86, 0,28) | 0,34 (-0,20, 0,87) | 0,04 (-0,36, 0,43) | 0.12 |
| Attitudinal 4th, Standard care 4th | -0,26 (-0,75, 0,19) | -0,89 (-1,52, -0,26) | -0,50 (-0,88, -0,12) | 0.12 |
| Educational 4th, Educational + Attitudinal 4th | 0,28 (-0,74, 1,28) | -0,32 (-0,91, 0,25) | -0,18 (-0,67, 0,33) | 0.32 |
| Educational 4th, Educational + Technical 4th | -0,43 (-1,44, 0,57) | 0,09 (-0,27, 0,45) | 0,03 (-0,30, 0,39) | 0.33 |
| Educational 4th, Standard care 4th | -0,62 (-0,90, -0,33) | -0,26 (-0,79, 0,28) | -0,53 (-0,79, -0,27) | 0.23 |
| Educational + Attitudinal 4th, Standard care 4th | -0,24 (-0,76, 0,27) | -0,86 (-1,91, 0,18) | -0,36 (-0,82, 0,10) | 0.28 |
| Educational + Technical 4th, Standard care 4th | -0,60 (-0,86, -0,36) | -0,19 (-0,88, 0,51) | -0,57 (-0,82, -0,33) | 0.27 |
| Educational + Technical 4th, Technical 4th | 0,21 (-0,70, 1,12) | -0,07 (-0,46, 0,31) | -0,06 (-0,42, 0,30) | 0.56 |
