# Supplementary material for: Temporal effectiveness of interventions to improve medication adherence: A network meta-analysis
Source: PLoS One. 2019 Mar 12;14(3):e0213432. doi: 10.1371/journal.pone.0213432 (PMC6413898; doi:10.1371/journal.pone.0213432)
Supplement: S6 Table — (DOCX) [file pone.0213432.s006.docx]

**S6 Table. Final** **rank orders from SUCRA analyses**
