# Supplementary material for: Temporal effectiveness of interventions to improve medication adherence: A network meta-analysis
Source: PLoS One. 2019 Mar 12;14(3):e0213432. doi: 10.1371/journal.pone.0213432 (PMC6413898; doi:10.1371/journal.pone.0213432)
Supplement: S1 Appendix — a. Included meta-analysesb. Included primary studiesc. Excluded primary studiesd. Studies included in the network meta-analysis (DOCX) [file pone.0213432.s007.docx]

**S1 Appendix. Complete references**

- 1. **Included meta-analyses**

1. Bangalore S, Kamalakkannan G, Parkar S, Messerli FH. Fixed-dose combinations improve medication compliance: a meta-analysis. Am J Med. 2007;120(8):713-719.
2. Burudpakdee C, Khan ZM, Gala S, Nanavaty M, Kaura S. Impact of patient programs on adherence and persistence in inflammatory and immunologic diseases: a meta-analysis. Patient Prefer Adherence. 2015;9:435-448.
3. Caldeira D, Vaz-Carneiro A, Costa J. The impact of dosing frequency on medication adherence in chronic cardiovascular disease: systematic review and meta-analysis. Rev Port Cardiol. 2014;33(7-8):431-437.
4. Chase JA, Bogener JL, Ruppar TM, Conn VS. The Effectiveness of Medication Adherence Interventions Among Patients With Coronary Artery Disease: A Meta-analysis. J Cardiovasc Nurs. 2016;31(4):357-366.
5. Conn VS, Enriquez M, Ruppar TM, Chan KC. Cultural relevance in medication adherence interventions with underrepresented adults: systematic review and meta-analysis of outcomes. Prev Med. 2014;69:239-247.
6. Conn VS, Hafdahl AR, Cooper PS, Ruppar TM, Mehr DR, Russell CL. Interventions to improve medication adherence among older adults: meta-analysis of adherence outcomes among randomized controlled trials. Gerontologist. 2009;49(4):447-462.
7. Conn VS, Ruppar TM, Chan KC, Dunbar-Jacob J, Pepper GA, De Geest S. Packaging interventions to increase medication adherence: systematic review and meta-analysis. Curr Med Res Opin. 2015;31(1):145-160.
8. Conn VS, Ruppar TM, Chase JA, Enriquez M, Cooper PS. Interventions to Improve Medication Adherence in Hypertensive Patients: Systematic Review and Meta-analysis. Curr Hypertens Rep. 2015;17(12):94.
9. Conn VS, Ruppar TM, Enriquez M, Cooper P. Medication adherence interventions that target subjects with adherence problems: Systematic review and meta-analysis. Res Social Adm Pharm. 2016;12(2):218-246.
10. Crawshaw J, Auyeung V, Ashworth L, Norton S, Weinman J. Healthcare provider-led interventions to support medication adherence following ACS: a meta-analysis. Open heart. 2017;4(2):e000685.
11. de Bruin M, Viechtbauer W, Schaalma HP, Kok G, Abraham C, Hospers HJ. Standard care impact on effects of highly active antiretroviral therapy adherence interventions: A meta-analysis of randomized controlled trials. Arch Intern Med. 2010;170(3):240-250.
12. De Simoni A, Hardeman W, Mant J, Farmer AJ, Kinmonth AL. Trials to improve blood pressure through adherence to antihypertensives in stroke/TIA: systematic review and meta-analysis. J Am Heart Assoc. 2013;2(4):e000251.
13. Demonceau J, Ruppar T, Kristanto P, et al. Identification and assessment of adherence-enhancing interventions in studies assessing medication adherence through electronically compiled drug dosing histories: a systematic literature review and meta-analysis. Drugs. 2013;73(6):545-562.
14. Dokbua S, Dilokthornsakul P, Chaiyakunapruk N, Saini B, Krass I, Dhippayom T. Effects of an Asthma Self-Management Support Service Provided by Community Pharmacists: A Systematic Review and Meta-Analysis. Journal of managed care & specialty pharmacy. 2018;24(11):1184-96.
15. Easthall C, Song F, Bhattacharya D. A meta-analysis of cognitive-based behaviour change techniques as interventions to improve medication adherence. BMJ Open. 2013;3(8).
16. Falagas ME, Karagiannis AK, Nakouti T, Tansarli GS. Compliance with once-daily versus twice or thrice-daily administration of antibiotic regimens: a meta-analysis of randomized controlled trials. PLoS One. 2015;10(1):e0116207.
17. Fenerty SD, West C, Davis SA, Kaplan SG, Feldman SR. The effect of reminder systems on patients' adherence to treatment. Patient Prefer Adherence. 2012;6:127-135.
18. Finitsis DJ, Pellowski JA, Johnson BT. Text message intervention designs to promote adherence to antiretroviral therapy (ART): a meta-analysis of randomized controlled trials. PLoS One. 2014;9(2):e88166.
19. Finitsis DJ, Vose BA, Mahalak JG, Salner AL. Interventions to promote adherence to endocrine therapy among breast cancer survivors: A meta-analysis. Psycho-oncology. 2018.
20. Fletcher BR, Hartmann-Boyce J, Hinton L, McManus RJ. The Effect of Self-Monitoring of Blood Pressure on Medication Adherence and Lifestyle Factors: A Systematic Review and Meta-Analysis. Am J Hypertens. 2015;28(10):1209-1221.
21. Gray R, Bressington D, Ivanecka A, et al. Is adherence therapy an effective adjunct treatment for patients with schizophrenia spectrum disorders? A systematic review and meta-analysis. BMC Psychiatry. 2016;16:90.
22. Hart JE, Jeon CY, Ivers LC, et al. Effect of directly observed therapy for highly active antiretroviral therapy on virologic, immunologic, and adherence outcomes: a meta-analysis and systematic review. J Acquir Immune Defic Syndr. 2010;54(2):167-179.
23. Heneghan CJ, Glasziou P, Perera R. Reminder packaging for improving adherence to self-administered long-term medications. Cochrane Database Syst Rev. 2006(1):CD005025.
24. Hollands GJ, McDermott MS, Lindson-Hawley N, Vogt F, Farley A, Aveyard P. Interventions to increase adherence to medications for tobacco dependence. Cochrane Database Syst Rev. 2015(2):CD009164.
25. Horvath T, Azman H, Kennedy GE, Rutherford GW. Mobile phone text messaging for promoting adherence to antiretroviral therapy in patients with HIV infection. Cochrane Database Syst Rev. 2012(3):CD009756.
26. Iglay K, Cao X, Mavros P, Joshi K, Yu S, Tunceli K. Systematic Literature Review and Meta-analysis of Medication Adherence With Once-weekly Versus Once-daily Therapy. Clin Ther. 2015;37(8):1813-1821 e1811.
27. Iskedjian M, Einarson TR, MacKeigan LD, et al. Relationship between daily dose frequency and adherence to antihypertensive pharmacotherapy: evidence from a meta-analysis. Clin Ther. 2002;24(2):302-316.
28. Kanters S, Park JJ, Chan K, et al. Use of peers to improve adherence to antiretroviral therapy: a global network meta-analysis. J Int AIDS Soc. 2016;19(1):21141.
29. Kanters S, Park JJ, Chan K, et al. Interventions to improve adherence to antiretroviral therapy: a systematic review and network meta-analysis. Lancet HIV. 2017;4(1):e31-e40.
30. Kassavou A, Sutton S. Automated telecommunication interventions to promote adherence to cardio-metabolic medications: meta-analysis of effectiveness and meta-regression of behaviour change techniques. Health Psychol Rev. 2018;12(1):25-42.
31. Kauppi K, Valimaki M, Hatonen HM, Kuosmanen LM, Warwick-Smith K, Adams CE. Information and communication technology based prompting for treatment compliance for people with serious mental illness. Cochrane Database Syst Rev. 2014(6):CD009960.
32. Langebeek N, Nieuwkerk P. Electronic medication monitoring-informed counseling to improve adherence to combination anti-retroviral therapy and virologic treatment outcomes: a meta-analysis. Front Public Health. 2015;3:139.
33. MacDonald L, Chapman S, Syrett M, Bowskill R, Horne R. Improving medication adherence in bipolar disorder: A systematic review and meta-analysis of 30 years of intervention trials. J Affect Disord. 2016;194:202-221.
34. Mahtani KR, Heneghan CJ, Glasziou PP, Perera R. Reminder packaging for improving adherence to self-administered long-term medications. Cochrane Database Syst Rev. 2011(9):CD005025.
35. Manias E, Williams A. Medication adherence in people of culturally and linguistically diverse backgrounds: a meta-analysis. Ann Pharmacother. 2010;44(6):964-982.
36. Mes MA, Katzer CB, Chan AHY, Wileman V, Taylor SJC, Horne R. Pharmacists and medication adherence in asthma: a systematic review and meta-analysis. The European respiratory journal. 2018;52(2).
37. Miller L, Schuz B, Walters J, Walters EH. Mobile Technology Interventions for Asthma Self-Management: Systematic Review and Meta-Analysis. JMIR Mhealth Uhealth. 2017;5(5):e57.
38. Morrissey EC, Durand H, Nieuwlaat R, et al. Effectiveness and content analysis of interventions to enhance medication adherence and blood pressure control in hypertension: A systematic review and meta-analysis. Psychol Health. 2017;32(10):1195-1232.
39. Mutasa-Apollo T, Ford N, Wiens M, et al. Effect of frequency of clinic visits and medication pick-up on antiretroviral treatment outcomes: a systematic literature review and meta-analysis. J Int AIDS Soc. 2017;20(Suppl 4):21647.
40. Normansell R, Kew KM, Stovold E. Interventions to improve adherence to inhaled steroids for asthma. Cochrane Database Syst Rev. 2017;4:CD012226.
41. Omonaiye O, Nicholson P, Kusljic S, Manias E. A meta-analysis of effectiveness of interventions to improve adherence in pregnant women receiving antiretroviral therapy in sub-Saharan Africa. International journal of infectious diseases : IJID : official publication of the International Society for Infectious Diseases. 2018;74:71-82.
42. Palacio A, Garay D, Langer B, Taylor J, Wood BA, Tamariz L. Motivational Interviewing Improves Medication Adherence: a Systematic Review and Meta-analysis. J Gen Intern Med. 2016;31(8):929-940.
43. Parappilly BP, Field TS, Mortenson WB, Sakakibara BM, Eng JJ. Effectiveness of interventions involving nurses in secondary stroke prevention: A systematic review and meta-analysis. European journal of cardiovascular nursing : journal of the Working Group on Cardiovascular Nursing of the European Society of Cardiology. 2018;17(8):728-36.
44. Parienti JJ, Bangsberg DR, Verdon R, Gardner EM. Better adherence with once-daily antiretroviral regimens: a meta-analysis. Clin Infect Dis. 2009;48(4):484-488.
45. Peterson AM, Takiya L, Finley R. Meta-analysis of trials of interventions to improve medication adherence. Am J Health Syst Pharm. 2003;60(7):657-665.
46. Petry NM, Rash CJ, Byrne S, Ashraf S, White WB. Financial reinforcers for improving medication adherence: findings from a meta-analysis. Am J Med. 2012;125(9):888-896.
47. Rash JA, Campbell DJ, Tonelli M, Campbell TS. A systematic review of interventions to improve adherence to statin medication: What do we know about what works? Prev Med. 2016;90:155-169.
48. Readdean KC, Heuer AJ, Scott Parrott J. Effect of pharmacist intervention on improving antidepressant medication adherence and depression symptomology: A systematic review and meta-analysis. Res Social Adm Pharm. 2017.
49. Rocha BS, Silveira MP, Moraes CG, Kuchenbecker RS, Dal-Pizzol TS. Pharmaceutical interventions in antiretroviral therapy: systematic review and meta-analysis of randomized clinical trials. J Clin Pharm Ther. 2015;40(3):251-258.
50. Rubio-Valera M, Serrano-Blanco A, Magdalena-Belio J, et al. Effectiveness of pharmacist care in the improvement of adherence to antidepressants: a systematic review and meta-analysis. Ann Pharmacother. 2011;45(1):39-48.
51. Ruppar TM, Delgado JM, Temple J. Medication adherence interventions for heart failure patients: A meta-analysis. Eur J Cardiovasc Nurs. 2015;14(5):395-404.
52. Ruppar TM, Dunbar-Jacob JM, Mehr DR, Lewis L, Conn VS. Medication adherence interventions among hypertensive black adults: a systematic review and meta-analysis. J Hypertens. 2017;35(6):1145-1154.
53. Sakakibara BM, Kim AJ, Eng JJ. A Systematic Review and Meta-Analysis on Self-Management for Improving Risk Factor Control in Stroke Patients. Int J Behav Med. 2017;24(1):42-53.
54. Santo K, Kirkendall S, Laba TL, et al. Interventions to improve medication adherence in coronary disease patients: A systematic review and meta-analysis of randomised controlled trials. Eur J Prev Cardiol. 2016;23(10):1065-1076.
55. Simoni JM, Pearson CR, Pantalone DW, Marks G, Crepaz N. Efficacy of interventions in improving highly active antiretroviral therapy adherence and HIV-1 RNA viral load. A meta-analytic review of randomized controlled trials. J Acquir Immune Defic Syndr. 2006;43 Suppl 1:S23-35.
56. Sin NL, DiMatteo MR. Depression treatment enhances adherence to antiretroviral therapy: a meta-analysis. Ann Behav Med. 2014;47(3):259-269.
57. Spaan P, van Luenen S, Garnefski N, Kraaij V. Psychosocial interventions enhance HIV medication adherence: A systematic review and meta-analysis. Journal of health psychology. 2018:1359105318755545.
58. Takiya LN, Peterson AM, Finley RS. Meta-analysis of interventions for medication adherence to antihypertensives. Ann Pharmacother. 2004;38(10):1617-1624.
59. Tao D, Xie L, Wang T, Wang T. A meta-analysis of the use of electronic reminders for patient adherence to medication in chronic disease care. J Telemed Telecare. 2015;21(1):3-13.
60. Thakkar J, Kurup R, Laba TL, et al. Mobile Telephone Text Messaging for Medication Adherence in Chronic Disease: A Meta-analysis. JAMA Intern Med. 2016;176(3):340-349.
61. Tsoli S, Sutton S, Kassavou A. Interactive voice response interventions targeting behaviour change: a systematic literature review with meta-analysis and meta-regression. BMJ open. 2018;8(2):e018974.
62. Van Camp YP, Van Rompaey B, Elseviers MM. Nurse-led interventions to enhance adherence to chronic medication: systematic review and meta-analysis of randomised controlled trials. Eur J Clin Pharmacol. 2013;69(4):761-770.
63. van Driel ML, Morledge MD, Ulep R, Shaffer JP, Davies P, Deichmann R. Interventions to improve adherence to lipid-lowering medication. Cochrane Database Syst Rev. 2016;12:CD004371.
64. van Galen KA, Nellen JF, Nieuwkerk PT. The Effect on Treatment Adherence of Administering Drugs as Fixed-Dose Combinations versus as Separate Pills: Systematic Review and Meta-Analysis. AIDS Res Treat. 2014;2014:967073.
65. Vignon Zomahoun HT, de Bruin M, Guillaumie L, et al. Effectiveness and Content Analysis of Interventions to Enhance Oral Antidiabetic Drug Adherence in Adults with Type 2 Diabetes: Systematic Review and Meta-Analysis. Value Health. 2015;18(4):530-540.
66. Wald DS, Butt S, Bestwick JP. One-way versus two-way text messaging on improving medication adherence: meta-analysis of randomized trials. Am J Med. 2015;128(10):1139 e1131-1135.
67. Zhu Y, Zhou Y, Zhang L, Zhang J, Lin J. Efficacy of interventions for adherence to the immunosuppressive therapy in kidney transplant recipients: a meta-analysis and systematic review. J Investig Med. 2017;65(7):1049-1056.
68. Zomahoun HTV, Guenette L, Gregoire JP, et al. Effectiveness of motivational interviewing interventions on medication adherence in adults with chronic diseases: a systematic review and meta-analysis. Int J Epidemiol. 2017;46(2):589-602.
69. Zou H, Li Z, Nolan MT, Arthur D, Wang H, Hu L. Self-management education interventions for persons with schizophrenia: a meta-analysis. Int J Ment Health Nurs. 2013;22(3):256-271.
    1. **References from all included original studies in qualitative review**
70. Adeyemo A, Tayo BO, Luke A, Ogedegbe O, Durazo-Arvizu R, Cooper RS. The Nigerian antihypertensive adherence trial: a community-based randomized trial. J Hypertens. 2013;31(1):201-207.
71. Aguwa CN, Ukwe CV, Ekwunife OI. Effect of pharmaceutical care programme on blood pressure and quality of life in a Nigerian pharmacy. Pharm World Sci. 2008;30(1):107-110.
72. Alhalaiqa F, Deane KH, Nawafleh AH, Clark A, Gray R. Adherence therapy for medication non-compliant patients with hypertension: a randomised controlled trial. J Hum Hypertens. 2012;26(2):117-126.
73. Aljumah K, Hassali MA. Impact of pharmacist intervention on adherence and measurable patient outcomes among depressed patients: a randomised controlled study. BMC Psychiatry. 2015;15:219.
74. Al-Saffar N, Deshmukh AA, Carter P, Adib SM. Effect of information leaflets and counselling on antidepressant adherence: open randomised controlled trial in a psychiatric hospital in Kuwait. International Journal of Pharmacy Practice. 2005;13(2):123-131.
75. Altice FL, Maru DS, Bruce RD, Springer SA, Friedland GH. Superiority of directly administered antiretroviral therapy over self-administered therapy among HIV-infected drug users: a prospective, randomized, controlled trial. Clin Infect Dis. 2007;45(6):770-778.
76. Amado Guirado E, Pujol Ribera E, Pacheco Huergo V, Borras JM. Knowledge and adherence to antihypertensive therapy in primary care: results of a randomized trial. Gac Sanit. 2011;25(1):62-67.
77. Amado Guirado E, Pujol Ribera E, Pacheco Huergo V, Borras JM. Knowledge and adherence to antihypertensive therapy in primary care: results of a randomized trial. Gac Sanit. 2011;25(1):62-67.
78. Anderson KH, Ford S, Robson D, Cassis J, Rodrigues C, Gray R. An exploratory, randomized controlled trial of adherence therapy for people with schizophrenia. Int J Ment Health Nurs. 2010;19(5):340-349.
79. Andrade AS, McGruder HF, Wu AW, et al. A programmable prompting device improves adherence to highly active antiretroviral therapy in HIV-infected subjects with memory impairment. Clin Infect Dis. 2005;41(6):875-882.
80. Andrejak M, Genes N, Vaur L, Poncelet P, Clerson P, Carre A. Electronic pill-boxes in the evaluation of antihypertensive treatment compliance: comparison of once daily versus twice daily regimen. Am J Hypertens. 2000;13(2):184-190.
81. Antoni MH, Carrico AW, Duran RE, et al. Randomized clinical trial of cognitive behavioral stress management on human immunodeficiency virus viral load in gay men treated with highly active antiretroviral therapy. Psychosom Med. 2006;68(1):143-151.
82. Antonicelli R, Mazzanti I, Abbatecola AM, Parati G. Impact of home patient telemonitoring on use of beta-blockers in congestive heart failure. Drugs Aging. 2010;27(10):801-805.
83. Apter AJ, Wang X, Bogen DK, et al. Problem solving to improve adherence and asthma outcomes in urban adults with moderate or severe asthma: a randomized controlled trial. J Allergy Clin Immunol. 2011;128(3):516-523 e511-515.
84. Armour C, Bosnic-Anticevich S, Brillant M, Burton D, Emmerton L, Krass I, et al. Pharmacy Asthma Care Program (PACP) improves outcomes for patients in the community. Thorax. 2007;62(6):496-502.
85. Arora S, Peters AL, Burner E, Lam CN, Menchine M. Trial to examine text message-based mHealth in emergency department patients with diabetes (TExT-MED): a randomized controlled trial. Ann Emerg Med. 2014;63(6):745-754 e746.
86. Asplund J, Danielson M, Ohman P. Patients compliance in hypertension--the importance of number of tablets. Br J Clin Pharmacol. 1984;17(5):547-552.
87. Azrin NH, Teichner G. Evaluation of an instructional program for improving medication compliance for chronically mentally ill outpatients. Behav Res Ther. 1998;36(9):849-861.
88. Babamoto KS, Sey KA, Camilleri AJ, Karlan VJ, Catalasan J, Morisky DE. Improving diabetes care and health measures among hispanics using community health workers: results from a randomized controlled trial. Health Educ Behav. 2009;36(1):113-126.
89. Bailey B, Carney SL, Gillies AA, Smith AJ. Antihypertensive drug treatment: a comparison of usual care with self blood pressure measurement. J Hum Hypertens. 1999;13(2):147-150.
90. Bailey WC, Richards JM, Jr., Brooks CM, Soong SJ, Windsor RA, Manzella BA. A randomized trial to improve self-management practices of adults with asthma. Arch Intern Med. 1990;150(8):1664-1668.
91. Baird MG, Bentley-Taylor MM, Carruthers SG, et al. A study of efficacy, tolerance and compliance of once-daily versus twice-daily metoprolol (Betaloc) in hypertension. Betaloc Compliance Canadian Cooperative Study Group. Clin Invest Med. 1984;7(2):95-102.
92. Ball JR, Mitchell PB, Corry JC, Skillecorn A, Smith M, Malhi GS. A randomized controlled trial of cognitive therapy for bipolar disorder: focus on long-term change. J Clin Psychiatry. 2006;67(2):277-286.
93. Basso CR, Helena ET, Caraciolo JM, Paiva V, Nemes MI. Exploring ART intake scenes in a human rights-based intervention to improve adherence: a randomized controlled trial. AIDS and behavior. 2013;17(1):181-92.
94. Begley S, Livingstone C, Hodges N, Williamson V. Impact of domiciliary pharmacy visits on medication management in an elderly population. International Journal of Pharmacy Practice. 1997;5(3):111-121.
95. Bender BG, Apter A, Bogen DK, et al. Test of an interactive voice response intervention to improve adherence to controller medications in adults with asthma. J Am Board Fam Med. 2010;23(2):159-165.
96. Berg J, Dunbar-Jacob J, Sereika SM. An evaluation of a self-management program for adults with asthma. Clin Nurs Res. 1997;6(3):225-238.
97. Berg KM, Litwin A, Li X, Heo M, Arnsten JH. Directly observed antiretroviral therapy improves adherence and viral load in drug users attending methadone maintenance clinics: a randomized controlled trial. Drug Alcohol Depend. 2011;113(2-3):192-199.
98. Berger S, Schad T, von Wyl V, et al. Effects of cognitive behavioral stress management on HIV-1 RNA, CD4 cell counts and psychosocial parameters of HIV-infected persons. AIDS. 2008;22(6):767-775.
99. Bessa AB, Felipe CR, Hannun P, et al. Prospective Randomized Trial Investigating the Influence of Pharmaceutical Care on the Intra-Individual Variability of Tacrolimus Concentrations Early After Kidney Transplant. Ther Drug Monit. 2016;38(4):447-455.
100. Beune EJ, Moll van Charante EP, Beem L, et al. Culturally adapted hypertension education (CAHE) to improve blood pressure control and treatment adherence in patients of African origin with uncontrolled hypertension: cluster-randomized trial. PLoS One. 2014;9(3):e90103.
101. Bisharat B, Hafi L, Baron-Epel O, Armaly Z, Bowirrat A. Pharmacist counseling to cardiac patients in Israel prior to discharge from hospital contribute to increasing patient's medication adherence closing gaps and improving outcomes. J Transl Med. 2012;10:34.
102. Blenkinsopp A, Phelan M, Bourne J, Dakhil N. Extended adherence support by community pharmacists for patients with hypertension: a randomised controlled trial. International Journal of Pharmacy Practice. 2000;8(3):165-175.
103. Bobrow K, Farmer AJ, Springer D, et al. Mobile Phone Text Messages to Support Treatment Adherence in Adults With High Blood Pressure (SMS-Text Adherence Support [StAR]): A Single-Blind, Randomized Trial. Circulation. 2016;133(6):592-600.
104. Bogner HR, de Vries HF. Integration of depression and hypertension treatment: a pilot, randomized controlled trial. Ann Fam Med. 2008;6(4):295-301.
105. Bogner HR, de Vries HF. Integrating type 2 diabetes mellitus and depression treatment among African Americans: a randomized controlled pilot trial. Diabetes Educ. 2010;36(2):284-292.
106. Bogner HR, Morales KH, de Vries HF, Cappola AR. Integrated management of type 2 diabetes mellitus and depression treatment to improve medication adherence: a randomized controlled trial. Ann Fam Med. 2012;10(1):15-22.
107. Boissel JP, Meillard O, Perrin-Fayolle E, et al. Comparison between a bid and a tid regimen: improved compliance with no improved antihypertensive effect. The EOL Research Group. Eur J Clin Pharmacol. 1996;50(1-2):63-67.
108. Bosworth HB, Olsen MK, Neary A, et al. Take Control of Your Blood Pressure (TCYB) study: a multifactorial tailored behavioral and educational intervention for achieving blood pressure control. Patient Educ Couns. 2008;70(3):338-347.
109. Bouvy ML, Heerdink ER, Urquhart J, Grobbee DE, Hoes AW, Leufkens HG. Effect of a pharmacist-led intervention on diuretic compliance in heart failure patients: a randomized controlled study. J Card Fail. 2003;9(5):404-411.
110. Bove AA, Homko CJ, Santamore WP, Kashem M, Kerper M, Elliott DJ. Managing hypertension in urban underserved subjects using telemedicine--a clinical trial. Am Heart J. 2013;165(4):615-621.
111. Boyle BA, Jayaweera D, Witt MD, Grimm K, Maa JF, Seekins DW. Randomization to once-daily stavudine extended release/lamivudine/efavirenz versus a more frequent regimen improves adherence while maintaining viral suppression. HIV Clin Trials. 2008;9(3):164-176.
112. Brankin E, Walker M, Lynch N, Aspray T, Lis Y, Cowell W. The impact of dosing frequency on compliance and persistence with bisphosphonates among postmenopausal women in the UK: evidence from three databases. Curr Med Res Opin. 2006;22(7):1249-1256.
113. Broekhuizen K, van Poppel MN, Koppes LL, Kindt I, Brug J, van Mechelen W. Can multiple lifestyle behaviours be improved in people with familial hypercholesterolemia? Results of a parallel randomised controlled trial. PLoS One. 2012;7(12):e50032.
114. Brook OH, van Hout H, Stalman W, et al. A pharmacy-based coaching program to improve adherence to antidepressant treatment among primary care patients. Psychiatr Serv. 2005;56(4):487-489.
115. Brown BG, Bardsley J, Poulin D, et al. Moderate Dose, Three-Drug Therapy With Niacin, Lovastatin, and Colestipol to Reduce Low-Density Lipoprotein Cholesterol <100 mg/dl in Patients With Hyperlipidemia and Coronary Artery Disease. The American Journal of Cardiology. 1997;80(2):111-115.
116. Brown I, Sheeran P, Reuber M. Enhancing antiepileptic drug adherence: a randomized controlled trial. Epilepsy Behav. 2009;16(4):634-639.
117. Burrelle TN. Evaluation of an interdisciplinary compliance service for elderly hypertensives. Journal of Geriatric Drug Therapy. 1986;1(2):23-51.
118. Calvert SB, Kramer JM, Anstrom KJ, Kaltenbach LA, Stafford JA, Allen LaPointe NM. Patient-focused intervention to improve long-term adherence to evidence-based medications: a randomized trial. Am Heart J. 2012;163(4):657-665 e651.
119. Capoccia KL, Boudreau DM, Blough DK, et al. Randomized trial of pharmacist interventions to improve depression care and outcomes in primary care. Am J Health Syst Pharm. 2004;61(4):364-372.
120. Carrico AW, Antoni MH, Duran RE, Ironson G, Penedo F, Fletcher MA, et al. Reductions in depressed mood and denial coping during cognitive behavioral stress management with HIV-Positive gay men treated with HAART. Annals of behavioral medicine : a publication of the Society of Behavioral Medicine. 2006;31(2):155-64.
121. Carter BL, Doucette WR, Franciscus CL, Ardery G, Kluesner KM, Chrischilles EA. Deterioration in BP Control Following Discontinuation of a Physician/Pharmacist Collaborative Intervention. Pharmacotherapy. 2010;30(3):228-235.
122. Castellano JM, Sanz G, Penalvo JL, et al. A polypill strategy to improve adherence: results from the FOCUS project. J Am Coll Cardiol. 2014;64(20):2071-2082.
123. Castle D, Berk M, Berk L, Lauder S, Chamberlain J, Gilbert M. Pilot of group intervention for bipolar disorder. Int J Psychiatry Clin Pract. 2007;11(4):279-284.
124. Chaisson RE, Barnes GL, Hackman J, et al. A randomized, controlled trial of interventions to improve adherence to isoniazid therapy to prevent tuberculosis in injection drug users. Am J Med. 2001;110(8):610-615.
125. Chan SS, Leung DY, Abdullah AS, et al. Smoking-cessation and adherence intervention among Chinese patients with erectile dysfunction. Am J Prev Med. 2010;39(3):251-258.
126. Chan SS, Leung DY, Abdullah AS, Wong VT, Hedley AJ, Lam TH. A randomized controlled trial of a smoking reduction plus nicotine replacement therapy intervention for smokers not willing to quit smoking. Addiction. 2011;106(6):1155-1163.
127. Chang LW, Kagaayi J, Nakigozi G, et al. Effect of peer health workers on AIDS care in Rakai, Uganda: a cluster-randomized trial. PLoS One. 2010;5(6):e10923.
128. Charles T, Quinn D, Weatherall M, Aldington S, Beasley R, Holt S. An audiovisual reminder function improves adherence with inhaled corticosteroid therapy in asthma. J Allergy Clin Immunol. 2007;119(4):811-816.
129. Chatkin JM, Blanco DC, Scaglia N, Wagner MB, Fritscher CC. Impact of a low-cost and simple intervention in enhancing treatment adherence in a Brazilian asthma sample. J Asthma. 2006;43(4):263-266.
130. Cheung R, Sullens CM, Seal D, et al. The paradox of using a 7 day antibacterial course to treat urinary tract infections in the community. Br J Clin Pharmacol. 1988;26(4):391-398.
131. Chien WT, Mui JH, Cheung EF, Gray R. Effects of motivational interviewing-based adherence therapy for schizophrenia spectrum disorders: a randomized controlled trial. Trials. 2015;16:270.
132. Chisholm MA, Mulloy LL, Jagadeesan M, DiPiro JT. Impact of clinical pharmacy services on renal transplant patients' compliance with immunosuppressive medications. Clin Transplant. 2001;15(5):330-336.
133. Choudhry NK, Avorn J, Glynn RJ, et al. Full coverage for preventive medications after myocardial infarction. N Engl J Med. 2011;365(22):2088-2097.
134. Cizmic AD, Heilmann RM, Milchak JL, Riggs CS, Billups SJ. Impact of interactive voice response technology on primary adherence to bisphosphonate therapy: a randomized controlled trial. Osteoporosis international : a journal established as result of cooperation between the European Foundation for Osteoporosis and the National Osteoporosis Foundation of the USA. 2015;26(8):2131-6.
135. Claborn KR, Leffingwell TR, Miller MB, Meier E, Stephens JR. Pilot study examining the efficacy of an electronic intervention to promote HIV medication adherence. AIDS care. 2014;26(3):404-9.
136. Clowes JA, Peel NF, Eastell R. The impact of monitoring on adherence and persistence with antiresorptive treatment for postmenopausal osteoporosis: a randomized controlled trial. J Clin Endocrinol Metab. 2004;89(3):1117-1123.
137. Cochran SD. Preventing medical noncompliance in the outpatient treatment of bipolar affective disorders. J Consult Clin Psychol. 1984;52(5):873-878.
138. Cohen CJ, Kubota M, Brachman PS, et al. Short-term safety and tolerability of a once-daily fixed-dose abacavir-lamivudine combination versus twice-daily dosing of abacavir and lamivudine as separate components: findings from the ALOHA study. Pharmacotherapy. 2008;28(3):314-322.
139. Cole P, Emmanuel. Drug consultation: its significance to the discharged hospital patient and its relevance as a role for the pharmacist. Am J Hosp Pharm. 1971;28(12):954-960.
140. Collier AC, Ribaudo H, Mukherjee AL, Feinberg J, Fischl MA, Chesney M. A randomized study of serial telephone call support to increase adherence and thereby improve virologic outcome in persons initiating antiretroviral therapy. J Infect Dis. 2005;192(8):1398-1406.
141. Cordasco KM, Asch SM, Bell DS, et al. A low-literacy medication education tool for safety-net hospital patients. Am J Prev Med. 2009;37(6 Suppl 1):S209-216.
142. Cossette S, Frasure-Smith N, Dupuis J, Juneau M, Guertin MC. Randomized controlled trial of tailored nursing interventions to improve cardiac rehabilitation enrollment. Nursing research. 2012;61(2):111-20.
143. Cramer J, Vachon L, Desforges C, Sussman NM. Dose frequency and dose interval compliance with multiple antiepileptic medications during a controlled clinical trial. Epilepsia. 1995;36(11):1111-1117.
144. Cramer JA, Amonkar MM, Hebborn A, Altman R. Compliance and persistence with bisphosphonate dosing regimens among women with postmenopausal osteoporosis. Curr Med Res Opin. 2005;21(9):1453-1460.
145. Cramer JA, Lynch NO, Gaudin AF, Walker M, Cowell W. The effect of dosing frequency on compliance and persistence with bisphosphonate therapy in postmenopausal women: a comparison of studies in the United States, the United Kingdom, and France. Clin Ther. 2006;28(10):1686-1694.
146. Cramer JA, Rosenheck R. Enhancing medication compliance for people with serious mental illness. J Nerv Ment Dis. 1999;187(1):53-55.
147. Crockett J, Taylor S, Grabham A, Stanford P. Patient outcomes following an intervention involving community pharmacists in the management of depression. Aust J Rural Health. 2006;14(6):263-269.
148. Crome P, Curl B, Boswell M, Corless D, Lewis RR. Assessment of a new calendar pack--the 'C-Pak'. Age Ageing. 1982;11(4):275-279.
149. da Costa TM, Barbosa BJ, Gomes e Costa DA, et al. Results of a randomized controlled trial to assess the effects of a mobile SMS-based intervention on treatment adherence in HIV/AIDS-infected Brazilian women and impressions and satisfaction with respect to incoming messages. Int J Med Inform. 2012;81(4):257-269.
150. de Bruin M, Hospers HJ, van Breukelen GJ, Kok G, Koevoets WM, Prins JM. Electronic monitoring-based counseling to enhance adherence among HIV-infected patients: a randomized controlled trial. Health Psychol. 2010;29(4):421-428.
151. Delmas PD, Vrijens B, Eastell R, et al. Effect of monitoring bone turnover markers on persistence with risedronate treatment of postmenopausal osteoporosis. J Clin Endocrinol Metab. 2007;92(4):1296-1304.
152. Derose SF, Green K, Marrett E, et al. Automated outreach to increase primary adherence to cholesterol-lowering medications. JAMA Intern Med. 2013;173(1):38-43.
153. Detry JM, Block P, De Backer G, Degaute JP. Patient compliance and therapeutic coverage: comparison of amlodipine and slow release nifedipine in the treatment of hypertension. The Belgian Collaborative Study Group. Eur J Clin Pharmacol. 1995;47(6):477-481.
154. DiIorio C, McCarty F, Resnicow K, et al. Using motivational interviewing to promote adherence to antiretroviral medications: a randomized controlled study. AIDS Care. 2008;20(3):273-283.
155. DiIorio C, Reisinger EL, Yeager KA, McCarty F. A telephone-based self-management program for people with epilepsy. Epilepsy Behav. 2009;14(1):232-236.
156. DiIorio C, Resnicow K, McDonnell M, Soet J, McCarty F, Yeager K. Using motivational interviewing to promote adherence to antiretroviral medications: a pilot study. J Assoc Nurses AIDS Care. 2003;14(2):52-62.
157. Dogan S, Sabanciogullari S. The effects of patient education in lithium therapy on quality of life and compliance. Arch Psychiatr Nurs. 2003;17(6):270-275.
158. D'Souza R, Piskulic D, Sundram S. A brief dyadic group based psychoeducation program improves relapse rates in recently remitted bipolar disorder: a pilot randomised controlled trial. J Affect Disord. 2010;120(1-3):272-276.
159. Du L, Dong P, Jia J, Li Z, Lai L, Yang X, et al. Impacts of intensive follow-up on the long-term prognosis of percutaneous coronary intervention in acute coronary syndrome patients - a single center prospective randomized controlled study in a Chinese population. European journal of preventive cardiology. 2016;23(10):1077-85.
160. Dusing R, Handrock R, Klebs S, Tousset E, Vrijens B. Impact of supportive measures on drug adherence in patients with essential hypertension treated with valsartan: the randomized, open-label, parallel group study VALIDATE. J Hypertens. 2009;27(4):894-901.
161. Edworthy SM, Baptie B, Galvin D, et al. Effects of an enhanced secondary prevention program for patients with heart disease: a prospective randomized trial. Can J Cardiol. 2007;23(13):1066-1072.
162. Edworthy SM, Devins GM. Improving medication adherence through patient education distinguishing between appropriate and inappropriate utilization. Patient Education Study Group. J Rheumatol. 1999;26(8):1793-1801.
163. Eker F, Harkin S. Effectiveness of six-week psychoeducation program on adherence of patients with bipolar affective disorder. J Affect Disord. 2012;138(3):409-416.
164. Elixhauser A, Eisen SA, Romeis JC, Homan SM. The effects of monitoring and feedback on compliance. Med Care. 1990;28(10):882-893.
165. Elkjaer M, Shuhaibar M, Burisch J, et al. E-health empowers patients with ulcerative colitis: a randomised controlled trial of the web-guided 'Constant-care' approach. Gut. 2010;59(12):1652-1661.
166. Erickson SR, Ascione FJ, Kirking DM. Utility of electronic device for medication management in patients with hypertension: a pilot study. J Am Pharm Assoc (2003). 2005;45(1):88-91.
167. Eron JJ, Feinberg J, Kessler HA, et al. Once-daily versus twice-daily lopinavir/ritonavir in antiretroviral-naive HIV-positive patients: a 48-week randomized clinical trial. J Infect Dis. 2004;189(2):265-272.
168. Eron JJ, Yetzer ES, Ruane PJ, et al. Efficacy, safety, and adherence with a twice-daily combination lamivudine/zidovudine tablet formulation, plus a protease inhibitor, in HIV infection. AIDS. 2000;14(6):671-681.
169. Eshelman FN, Fitzloff J. Effect of packaging on patient compliance with an antihypertensive medication. Curr Ther Res Clin Exp. 1976;20(2):215-219.
170. Eussen SR, van der Elst ME, Klungel OH, et al. A pharmaceutical care program to improve adherence to statin therapy: a randomized controlled trial. Ann Pharmacother. 2010;44(12):1905-1913.
171. Evans CD, Eurich DT, Taylor JG, Blackburn DF. The Collaborative Cardiovascular Risk Reduction in Primary Care (CCARP) study. Pharmacotherapy. 2010;30(8):766-775.
172. Evans-Hudnall GL, Stanley MA, Clark AN, et al. Improving secondary stroke self-care among underserved ethnic minority individuals: a randomized clinical trial of a pilot intervention. J Behav Med. 2014;37(2):196-204.
173. Falces C, Lopez-Cabezas C, Andrea R, Arnau A, Ylla M, Sadurni J. [An educative intervention to improve treatment compliance and to prevent readmissions of elderly patients with heart failure]. Med Clin (Barc). 2008;131(12):452-456.
174. Farmer A, Hardeman W, Hughes D, et al. An explanatory randomised controlled trial of a nurse-led, consultation-based intervention to support patients with adherence to taking glucose lowering medication for type 2 diabetes. BMC Fam Pract. 2012;13:30.
175. Farmer KC, Jacobs EW, Phillips CR. Long-term patient compliance with prescribed regimens of calcium channel blockers. Clin Ther. 1994;16(2):316-326; discussion 271-312.
176. Faulkner MA, Wadibia EC, Lucas BD, Hilleman DE. Impact of pharmacy counseling on compliance and effectiveness of combination lipid-lowering therapy in patients undergoing coronary artery revascularization: a randomized, controlled trial. Pharmacotherapy. 2000;20(4):410-416.
177. Fernandez S, Scales KL, Pineiro JM, Schoenthaler AM, Ogedegbe G. A senior center-based pilot trial of the effect of lifestyle intervention on blood pressure in minority elderly people with hypertension. J Am Geriatr Soc. 2008;56(10):1860-1866.
178. Finley PR, Rens HR, Pont JT, et al. Impact of a collaborative pharmacy practice model on the treatment of depression in primary care. Am J Health Syst Pharm. 2002;59(16):1518-1526.
179. Finley PR, Rens HR, Pont JT, et al. Impact of a collaborative care model on depression in a primary care setting: a randomized controlled trial. Pharmacotherapy. 2003;23(9):1175-1185.
180. Fisher JD, Amico KR, Fisher WA, et al. Computer-based intervention in HIV clinical care setting improves antiretroviral adherence: the LifeWindows Project. AIDS Behav. 2011;15(8):1635-1646.
181. Foster JM, Usherwood T, Smith L, et al. Inhaler reminders improve adherence with controller treatment in primary care patients with asthma. J Allergy Clin Immunol. 2014;134(6):1260-1268 e1263.
182. Friedberg JP, Rodriguez MA, Watsula ME, et al. Effectiveness of a tailored behavioral intervention to improve hypertension control: primary outcomes of a randomized controlled trial. Hypertension. 2015;65(2):440-446.
183. Friedman RH, Kazis LE, Jette A, et al. A telecommunications system for monitoring and counseling patients with hypertension. Impact on medication adherence and blood pressure control. Am J Hypertens. 1996;9(4 Pt 1):285-292.
184. Fulmer TT, Feldman PH, Kim TS, et al. An intervention study to enhance medication compliance in community-dwelling elderly individuals. J Gerontol Nurs. 1999;25(8):6-14.
185. Fyllingen G, Arnesen AR, Ronnevig J. Phenoxymethylpenicillin two or three times daily in bacterial upper respiratory tract infections: a blinded, randomized and controlled clinical study. Scand J Infect Dis. 1991;23(6):755-761.
186. Gabriel M, Gagnon JP, Bryan CK. Improved patients compliance through use of a daily drug reminder chart. Am J Public Health. 1977;67(10):968-969.
187. Gallant JE, DeJesus E, Arribas JR, et al. Tenofovir DF, emtricitabine, and efavirenz vs. zidovudine, lamivudine, and efavirenz for HIV. N Engl J Med. 2006;354(3):251-260.
188. Gamble J, Stevenson M, Heaney LG. A study of a multi-level intervention to improve non-adherence in difficult to control asthma. Respir Med. 2011;105(9):1308-1315.
189. Garcia MF, Bravin AM, Garcia PD, et al. Behavioral measures to reduce non-adherence in renal transplant recipients: a prospective randomized controlled trial. Int Urol Nephrol. 2015;47(11):1899-1905.
190. Garcia-Cardenas V, Sabater-Hernandez D, Kenny P, Martinez-Martinez F, Faus MJ, Benrimoj SI. Effect of a pharmacist intervention on asthma control. A cluster randomised trial. Respiratory medicine. 2013;107(9):1346-55.
191. Garnett WR, Davis LJ, McKenney JM, Steiner KC. Effect of telephone follow-up on medication compliance. Am J Hosp Pharm. 1981;38(5):676-679.
192. Gatwood J, Balkrishnan R, Erickson SR, An LC, Piette JD, Farris KB. The impact of tailored text messages on health beliefs and medication adherence in adults with diabetes: A randomized pilot study. Res Social Adm Pharm. 2016;12(1):130-140.
193. Gazmararian J, Jacobson KL, Pan Y, Schmotzer B, Kripalani S. Effect of a pharmacy-based health literacy intervention and patient characteristics on medication refill adherence in an urban health system. Ann Pharmacother. 2010;44(1):80-87.
194. Geiter LJ, O'Brien RJ, Combs DL, Snider DE, Jr. United States Public Health Service Tuberculosis Therapy Trial 21: preliminary results of an evaluation of a combination tablet of isoniazid, rifampin and pyrazinamide. Tubercle. 1987;68(2 Suppl):41-46.
195. Girvin B, McDermott BJ, Johnston GD. A comparison of enalapril 20 mg once daily versus 10 mg twice daily in terms of blood pressure lowering and patient compliance. J Hypertens. 1999;17(11):1627-1631.
196. Glanz K, Beck AD, Bundy L, et al. Impact of a health communication intervention to improve glaucoma treatment adherence. Results of the interactive study to increase glaucoma adherence to treatment trial. Arch Ophthalmol. 2012;130(10):1252-1258.
197. Goggin K, Gerkovich MM, Williams KB, et al. A randomized controlled trial examining the efficacy of motivational counseling with observed therapy for antiretroviral therapy adherence. AIDS Behav. 2013;17(6):1992-2001.
198. Golin CE, Earp J, Tien HC, Stewart P, Porter C, Howie L. A 2-arm, randomized, controlled trial of a motivational interviewing-based intervention to improve adherence to antiretroviral therapy (ART) among patients failing or initiating ART. J Acquir Immune Defic Syndr. 2006;42(1):42-51.
199. Gonzalez-Fernandez RA, Rivera M, Torres D, Quiles J, Jackson A. Usefulness of a systemic hypertension in-hospital educational program. Am J Cardiol. 1990;65(20):1384-1386.
200. Goodyer LI, Miskelly F, Milligan P. Does encouraging good compliance improve patients' clinical condition in heart failure? Br J Clin Pract. 1995;49(4):173-176.
201. Goswami NJ, Dekoven M, Kuznik A, et al. Impact of an integrated intervention program on atorvastatin adherence: a randomized controlled trial. Int J Gen Med. 2013;6:647-655.
202. Goujard C, Bernard N, Sohier N, et al. Impact of a patient education program on adherence to HIV medication: a randomized clinical trial. J Acquir Immune Defic Syndr. 2003;34(2):191-194.
203. Granger BB, Ekman I, Hernandez AF, et al. Results of the Chronic Heart Failure Intervention to Improve MEdication Adherence study: A randomized intervention in high-risk patients. Am Heart J. 2015;169(4):539-548.
204. Grant RW, Devita NG, Singer DE, Meigs JB. Improving adherence and reducing medication discrepancies in patients with diabetes. Ann Pharmacother. 2003;37(7-8):962-969.
205. Gray R, Leese M, Bindman J, et al. Adherence therapy for people with schizophrenia. European multicentre randomised controlled trial. Br J Psychiatry. 2006;189:508-514.
206. Green BB, Anderson ML, Cook AJ, et al. e-Care for heart wellness: a feasibility trial to decrease blood pressure and cardiovascular risk. Am J Prev Med. 2014;46(4):368-377.
207. Greer DB, Ostwald SK. Improving adherence in African American women with uncontrolled hypertension. J Cardiovasc Nurs. 2015;30(4):311-318.
208. Gross R, Bellamy SL, Chapman J, et al. Managed problem solving for antiretroviral therapy adherence: a randomized trial. JAMA Intern Med. 2013;173(4):300-306.
209. Gross R, Tierney C, Andrade A, et al. Modified directly observed antiretroviral therapy compared with self-administered therapy in treatment-naive HIV-1-infected patients: a randomized trial. Arch Intern Med. 2009;169(13):1224-1232.
210. Gross R, Zheng L, La Rosa A, et al. Partner-Focused Adherence Intervention for Second-line Antiretroviral Therapy: A Multinational Randomized Trial (ACTG A5234). Lancet HIV. 2015;2(1):e12-e19.
211. Grymonpre RE, Williamson DA, Montgomery PR. Impact of a pharmaceutical care model for non-institutionalised elderly: results of a randomised, controlled trial. International Journal of Pharmacy Practice. 2001;9(4):235-241.
212. Gujral G, Winckel K, Nissen LM, Cottrell WN. Impact of community pharmacist intervention discussing patients' beliefs to improve medication adherence. Int J Clin Pharm. 2014;36(5):1048-1058.
213. Guthrie RM. The effects of postal and telephone reminders on compliance with pravastatin therapy in a national registry: results of the first myocardial infarction risk reduction program. Clin Ther. 2001;23(6):970-980.
214. Gwadry-Sridhar FH, Arnold JM, Zhang Y, Brown JE, Marchiori G, Guyatt G. Pilot study to determine the impact of a multidisciplinary educational intervention in patients hospitalized with heart failure. Am Heart J. 2005;150(5):982.
215. Hacihasanoglu R, Gozum S. The effect of patient education and home monitoring on medication compliance, hypertension management, healthy lifestyle behaviours and BMI in a primary health care setting. J Clin Nurs. 2011;20(5-6):692-705.
216. Hadji P, Blettner M, Harbeck N, Jackisch C, Luck HJ, Windemuth-Kieselbach C, et al. The Patient's Anastrozole Compliance to Therapy (PACT) Program: a randomized, in-practice study on the impact of a standardized information program on persistence and compliance to adjuvant endocrine therapy in postmenopausal women with early breast cancer. Annals of oncology : official journal of the European Society for Medical Oncology. 2013;24(6):1505-12.
217. Hardy H, Kumar V, Doros G, et al. Randomized controlled trial of a personalized cellular phone reminder system to enhance adherence to antiretroviral therapy. AIDS Patient Care STDS. 2011;25(3):153-161.
218. Hawkins DW, Fiedler FP, Douglas HL, Eschbach RC. Evaluation of a clinical pharmacist in caring for hypertensive and diabetic patients. Am J Hosp Pharm. 1979;36(10):1321-1325.
219. Haynes RB, Sackett DL, Gibson ES, et al. Improvement of medication compliance in uncontrolled hypertension. Lancet. 1976;1(7972):1265-1268.
220. Heisler M, Vijan S, Makki F, Piette JD. Diabetes control with reciprocal peer support versus nurse care management: a randomized trial. Ann Intern Med. 2010;153(8):507-515.
221. Henry A, Batey RG. Enhancing compliance not a prerequisite for effective eradication of Helicobacter pylori: the HelP Study. Am J Gastroenterol. 1999;94(3):811-815.
222. Hersch RK, Cook RF, Billings DW, et al. Test of a web-based program to improve adherence to HIV medications. AIDS Behav. 2013;17(9):2963-2976.
223. Hilleman DE, Mohiuddin SM, Lucas BD, Jr., Shinn B, Elsasser GN. Conversion from sustained-release to immediate-release calcium entry blockers: outcome in patients with mild-to-moderate hypertension. Clin Ther. 1993;15(6):1002-1010.
224. Hirsch JD, Gonzales M, Rosenquist A, Miller TA, Gilmer TP, Best BM. Antiretroviral therapy adherence, medication use, and health care costs during 3 years of a community pharmacy medication therapy management program for Medi-Cal beneficiaries with HIV/AIDS. J Manag Care Pharm. 2011;17(3):213-223.
225. Hirsch JD, Rosenquist A, Best BM, Miller TA, Gilmer TP. Evaluation of the first year of a pilot program in community pharmacy: HIV/AIDS medication therapy management for Medi-Cal beneficiaries. J Manag Care Pharm. 2009;15(1):32-41.
226. Ho LY, Camejo L, Kahook MY, Noecker R. Effect of audible and visual reminders on adherence in glaucoma patients using a commercially available dosing aid. Clin Ophthalmol. 2008;2(4):769-772.
227. Ho PM, Lambert-Kerzner A, Carey EP, et al. Multifaceted intervention to improve medication adherence and secondary prevention measures after acute coronary syndrome hospital discharge: a randomized clinical trial. JAMA Intern Med. 2014;174(2):186-193.
228. Holstad MM, DiIorio C, Kelley ME, Resnicow K, Sharma S. Group motivational interviewing to promote adherence to antiretroviral medications and risk reduction behaviors in HIV infected women. AIDS Behav. 2011;15(5):885-896.
229. Holstad MM, Essien JE, Ekong E, Higgins M, Teplinskiy I, Adewuyi MF. Motivational groups support adherence to antiretroviral therapy and use of risk reduction behaviors in HIV positive Nigerian women: a pilot study. Afr J Reprod Health. 2012;16(3):14-27.
230. Holzemer WL, Bakken S, Portillo CJ, et al. Testing a nurse-tailored HIV medication adherence intervention. Nurs Res. 2006;55(3):189-197.
231. Homer D, Nightingale P, Jobanputra P. Providing patients with information about disease-modifying anti-rheumatic drugs: Individually or in groups? A pilot randomized controlled trial comparing adherence and satisfaction. Musculoskeletal Care. 2009;7(2):78-92.
232. Hornnes N, Larsen K, Boysen G. Blood pressure 1 year after stroke: the need to optimize secondary prevention. J Stroke Cerebrovasc Dis. 2011;20(1):16-23.
233. Horvath KJ, Oakes JM, Rosser BR, Danilenko G, Vezina H, Amico KR, et al. Feasibility, acceptability and preliminary efficacy of an online peer-to-peer social support ART adherence intervention. AIDS and behavior. 2013;17(6):2031-44.
234. Hosseininasab M, Jahangard-Rafsanjani Z, Mohagheghi A, et al. Self-monitoring of blood pressure for improving adherence to antihypertensive medicines and blood pressure control: a randomized controlled trial. Am J Hypertens. 2014;27(11):1339-1345.
235. Hunt JS, Siemienczuk J, Pape G, et al. A randomized controlled trial of team-based care: impact of physician-pharmacist collaboration on uncontrolled hypertension. J Gen Intern Med. 2008;23(12):1966-1972.
236. Ingersoll KS, Farrell-Carnahan L, Cohen-Filipic J, et al. A pilot randomized clinical trial of two medication adherence and drug use interventions for HIV+ crack cocaine users. Drug Alcohol Depend. 2011;116(1-3):177-187.
237. Interian A, Lewis-Fernandez R, Gara MA, Escobar JI. A randomized-controlled trial of an intervention to improve antidepressant adherence among Latinos with depression. Depress Anxiety. 2013;30(7):688-696.
238. Janson SL, Fahy JV, Covington JK, Paul SM, Gold WM, Boushey HA. Effects of individual self-management education on clinical, biological, and adherence outcomes in asthma. Am J Med. 2003;115(8):620-626.
239. Jarab AS, Alqudah SG, Mukattash TL, Shattat G, Al-Qirim T. Randomized controlled trial of clinical pharmacy management of patients with type 2 diabetes in an outpatient diabetes clinic in Jordan. J Manag Care Pharm. 2012;18(7):516-526.
240. Jerant AF, Azari R, Martinez C, Nesbitt TS. A randomized trial of telenursing to reduce hospitalization for heart failure: patient-centered outcomes and nursing indicators. Home Health Care Serv Q. 2003;22(1):1-20.
241. Jiang X, Sit JW, Wong TK. A nurse-led cardiac rehabilitation programme improves health behaviours and cardiac physiological risk parameters: evidence from Chengdu, China. J Clin Nurs. 2007;16(10):1886-1897.
242. Johnson AL, Taylor DW, Sackett DL, Dunnett CW, Shimizu AG. Self-recording of blood pressure in the management of hypertension. Can Med Assoc J. 1978;119(9):1034-1039.
243. Johnson MO, Charlebois E, Morin SF, Remien RH, Chesney MA. Effects of a behavioral intervention on antiretroviral medication adherence among people living with HIV: the healthy living project randomized controlled study. J Acquir Immune Defic Syndr. 2007;46(5):574-580.
244. Johnson MO, Dilworth SE, Taylor JM, Neilands TB. Improving coping skills for self-management of treatment side effects can reduce antiretroviral medication nonadherence among people living with HIV. Ann Behav Med. 2011;41(1):83-91.
245. Jones DL, Ishii M, LaPerriere A, et al. Influencing medication adherence among women with AIDS. AIDS Care. 2003;15(4):463-474.
246. Joost R, Dorje F, Schwitulla J, Eckardt KU, Hugo C. Intensified pharmaceutical care is improving immunosuppressive medication adherence in kidney transplant recipients during the first post-transplant year: a quasi-experimental study. Nephrol Dial Transplant. 2014;29(8):1597-1607.
247. Kalichman SC, Kalichman MO, Cherry C, et al. Brief behavioral self-regulation counseling for HIV treatment adherence delivered by cell phone: an initial test of concept trial. AIDS Patient Care STDS. 2011;25(5):303-310.
248. Kamal AK, Shaikh Q, Pasha O, et al. A randomized controlled behavioral intervention trial to improve medication adherence in adult stroke patients with prescription tailored Short Messaging Service (SMS)-SMS4Stroke study. BMC Neurol. 2015;15:212.
249. Kardas P. Comparison of once daily versus twice daily oral nitrates in stable angina pectoris. Am J Cardiol. 2004;94(2):213-216.
250. Kardas P. Comparison of patient compliance with once-daily and twice-daily antibiotic regimens in respiratory tract infections: results of a randomized trial. J Antimicrob Chemother. 2007;59(3):531-536.
251. Kardas P. An education-behavioural intervention improves adherence to statins. In. Open Medicine. Vol 82013:580.
252. Katon W, Robinson P, Von Korff M, et al. A multifaceted intervention to improve treatment of depression in primary care. Arch Gen Psychiatry. 1996;53(10):924-932.
253. Kauric-Klein Z. Improving blood pressure control in end stage renal disease through a supportive educative nursing intervention. Nephrol Nurs J. 2012;39(3):217-228.
254. Kellaway GS, McCrae E. The effect of counselling on compliance-failure in patient drug therapy. N Z Med J. 1979;89(631):161-165.
255. Kelly GR, Scott JE, Mamon J. Medication compliance and health education among outpatients with chronic mental disorders. Med Care. 1990;28(12):1181-1197.
256. Kertes J, Dushenat M, Vesterman JL, Lemberger J, Bregman J, Friedman N. Factors contributing to compliance with osteoporosis medication. Isr Med Assoc J. 2008;10(3):207-213.
257. Khonsari S, Subramanian P, Chinna K, Latif LA, Ling LW, Gholami O. Effect of a reminder system using an automated short message service on medication adherence following acute coronary syndrome. Eur J Cardiovasc Nurs. 2015;14(2):170-179.
258. Kim JI, Lee S, Kim JH. Effects of a web-based stroke education program on recurrence prevention behaviors among stroke patients: a pilot study. Health Educ Res. 2013;28(3):488-501.
259. Kim KB, Han HR, Huh B, Nguyen T, Lee H, Kim MT. The effect of a community-based self-help multimodal behavioral intervention in Korean American seniors with high blood pressure. Am J Hypertens. 2014;27(9):1199-1208.
260. Kim MT, Kim EY, Han HR, et al. Mail education is as effective as in-class education in hypertensive Korean patients. J Clin Hypertens (Greenwich). 2008;10(3):176-184.
261. Kiweewa FM, Wabwire D, Nakibuuka J, et al. Noninferiority of a task-shifting HIV care and treatment model using peer counselors and nurses among Ugandan women initiated on ART: evidence from a randomized trial. J Acquir Immune Defic Syndr. 2013;63(4):e125-132.
262. Klang SH, Ben-Amnon Y, Cohen Y, Barak Y. Community pharmacists' support improves antidepressant adherence in the community. Int Clin Psychopharmacol. 2015;30(6):316-319.
263. Klein A, Otto G, Kramer I. Impact of a pharmaceutical care program on liver transplant patients' compliance with immunosuppressive medication: a prospective, randomized, controlled trial using electronic monitoring. Transplantation. 2009;87(6):839-847.
264. Klein CE, Kastrissios H, Miller AA, et al. Pharmacokinetics, pharmacodynamics and adherence to oral topotecan in myelodysplastic syndromes: a Cancer and Leukemia Group B study. Cancer Chemother Pharmacol. 2006;57(2):199-206.
265. Koenig LJ, Pals SL, Bush T, Pratt Palmore M, Stratford D, Ellerbrock TV. Randomized controlled trial of an intervention to prevent adherence failure among HIV-infected patients initiating antiretroviral therapy. Health Psychol. 2008;27(2):159-169.
266. Kogos SC. Support Groups and Treatment Adherence in a Geriatric Outpatient Clinic. Journal of Clinical Psychology in Medical Settings. 2004;11(4):275-282.
267. Konkle-Parker DJ, Erlen JA, Dubbert PM, May W. Pilot testing of an HIV medication adherence intervention in a public clinic in the Deep South. J Am Acad Nurse Pract. 2012;24(8):488-498.
268. Konkle-Parker DJ, Amico KR, McKinney VE. Effects of an intervention addressing information, motivation, and behavioral skills on HIV care adherence in a southern clinic cohort. AIDS care. 2014;26(6):674-83.
269. Kooy MJ, van Wijk BLG, Heerdink ER, de Boer A, Bouvy ML. Does the use of an electronic reminder device with or without counseling improve adherence to lipid-lowering treatment? The results of a randomized controlled trial. Frontiers in Pharmacology. 2013;4:69.
270. Kopelowicz A, Zarate R, Gonzalez Smith V, Mintz J, Liberman RP. Disease management in Latinos with schizophrenia: a family-assisted, skills training approach. Schizophr Bull. 2003;29(2):211-227.
271. Kotowycz MA, Cosman TL, Tartaglia C, Afzal R, Syal RP, Natarajan MK. Safety and feasibility of early hospital discharge in ST-segment elevation myocardial infarction--a prospective and randomized trial in low-risk primary percutaneous coronary intervention patients (the Safe-Depart Trial). Am Heart J. 2010;159(1):117 e111-116.
272. Kozuki Y, Schepp KG. Visual-feedback therapy for antipsychotic medication adherence. Int Clin Psychopharmacol. 2006;21(1):57-61.
273. Krier BP, Parker RD, Grayson D, Byrd G. Effect of diabetes education on glucose control. J La State Med Soc. 1999;151(2):86-92.
274. Kripalani S, Schmotzer B, Jacobson TA. Improving Medication Adherence through Graphically Enhanced Interventions in Coronary Heart Disease (IMAGE-CHD): a randomized controlled trial. J Gen Intern Med. 2012;27(12):1609-1617.
275. Kronish IM, Rieckmann N, Burg MM, Edmondson D, Schwartz JE, Davidson KW. The effect of enhanced depression care on adherence to risk-reducing behaviors after acute coronary syndromes: findings from the COPES trial. American heart journal. 2012;164(4):524-9.
276. Kronish IM, Goldfinger JZ, Negron R, et al. The Effect of Peer Education on Stroke Prevention: the Prevent Recurrence of All Inner-City Strokes through Education (PRAISE) Randomized Controlled Trial. Stroke; a journal of cerebral circulation. 2014;45(11):3330-3336.
277. Kruse W, Eggert-Kruse W, Rampmaier J, Runnebaum B, Weber E. Dosage frequency and drug-compliance behaviour--a comparative study on compliance with a medication to be taken twice or four times daily. Eur J Clin Pharmacol. 1991;41(6):589-592.
278. Kurth AE, Spielberg F, Cleland CM, et al. Computerized counseling reduces HIV-1 viral load and sexual transmission risk: findings from a randomized controlled trial. J Acquir Immune Defic Syndr. 2014;65(5):611-620.
279. Lam DH, Bright J, Jones S, et al. Cognitive Therapy for Bipolar Illness—A Pilot Study of Relapse Prevention. Cognitive Therapy and Research. 2000;24(5):503-520.
280. Lam DH, Watkins ER, Hayward P, et al. A randomized controlled study of cognitive therapy for relapse prevention for bipolar affective disorder: outcome of the first year. Arch Gen Psychiatry. 2003;60(2):145-152.
281. Laramee AS, Levinsky SK, Sargent J, Ross R, Callas P. Case management in a heterogeneous congestive heart failure population: a randomized controlled trial. Arch Intern Med. 2003;163(7):809-817.
282. Lavoie KL, Moullec G, Lemiere C, et al. Efficacy of brief motivational interviewing to improve adherence to inhaled corticosteroids among adult asthmatics: results from a randomized controlled pilot feasibility trial. Patient Prefer Adherence. 2014;8:1555-1569.
283. Lee JK, Grace KA, Taylor AJ. Effect of a pharmacy care program on medication adherence and persistence, blood pressure, and low-density lipoprotein cholesterol: a randomized controlled trial. JAMA. 2006;296(21):2563-2571.
284. Lee JY, Kusek JW, Greene PG, et al. Assessing medication adherence by pill count and electronic monitoring in the African American Study of Kidney Disease and Hypertension (AASK) Pilot Study. Am J Hypertens. 1996;9(8):719-725.
285. Lee M, Kemp JA, Canning A, Egan C, Tataronis G, Farraye FA. A randomized controlled trial of an enhanced patient compliance program for Helicobacter pylori therapy. Arch Intern Med. 1999;159(19):2312-2316.
286. Leenen FH, Wilson TW, Bolli P, et al. Patterns of compliance with once versus twice daily antihypertensive drug therapy in primary care: a randomized clinical trial using electronic monitoring. Can J Cardiol. 1997;13(10):914-920.
287. Lester RT, Ritvo P, Mills EJ, et al. Effects of a mobile phone short message service on antiretroviral treatment adherence in Kenya (WelTel Kenya1): a randomised trial. Lancet. 2010;376(9755):1838-1845.
288. Levin TP, Klibanov OM, Axelrod P, et al. A randomized trial of educational materials, pillboxes, and mailings to improve adherence with antiretroviral therapy in an inner city HIV clinic. JCOM. 2006;13(4).
289. Levine DM, Green LW, Deeds SG, Chwalow J, Russell RP, Finlay J. Health education for hypertensive patients. JAMA. 1979;241(16):1700-1703.
290. Lin EH, Von Korff M, Ciechanowski P, et al. Treatment adjustment and medication adherence for complex patients with diabetes, heart disease, and depression: a randomized controlled trial. Ann Fam Med. 2012;10(1):6-14.
291. Linkewich JA, Catalano RB, Flack HL. The Effect of Packaging and Instruction on Outpatient Compliance with Medication Regimens. Drug Intelligence & Clinical Pharmacy. 1974;8(1):10-15.
292. Lipton HL, Bird JA. The impact of clinical pharmacists' consultations on geriatric patients' compliance and medical care use: a randomized controlled trial. Gerontologist. 1994;34(3):307-315.
293. Logan AG, Milne BJ, Achber C, Campbell WA, Haynes RB. A comparison of community and occupationally provided antihypertensive care. J Occup Med. 1982;24(11):901-906.
294. Lopez Cabezas C, Falces Salvador C, Cubi Quadrada D, et al. Randomized clinical trial of a postdischarge pharmaceutical care program vs regular follow-up in patients with heart failure. Farm Hosp. 2006;30(6):328-342.
295. Lourenco LB, Rodrigues RC, Ciol MA, et al. A randomized controlled trial of the effectiveness of planning strategies in the adherence to medication for coronary artery disease. J Adv Nurs. 2014;70(7):1616-1628.
296. Lourens H, Woodward MC. Impact of a Medication Card on Compliance in Older People. Australian Journal on Ageing. 1994;13(2):72-76.
297. Lua PL, Neni WS. A randomised controlled trial of an SMS-based mobile epilepsy education system. J Telemed Telecare. 2013;19(1):23-28.
298. Lucas GM, Mullen BA, Galai N, et al. Directly administered antiretroviral therapy for HIV-infected individuals in opioid treatment programs: results from a randomized clinical trial. PLoS One. 2013;8(7):e68286.
299. Lv Y, Zhao H, Liang Z, et al. A mobile phone short message service improves perceived control of asthma: a randomized controlled trial. Telemed J E Health. 2012;18(6):420-426.
300. M A Jalal ZS, Smith F, Taylor D, Finlay K, Patel H, Antoniou S. Impact of pharmacy care upon adherence to cardiovascular medicines: a feasibility pilot controlled trial. European Journal of Hospital Pharmacy. 2016;23(5):250-6.
301. Ma C, Zhou Y, Zhou W, Huang C. Evaluation of the effect of motivational interviewing counselling on hypertension care. Patient Educ Couns. 2014;95(2):231-237.
302. Ma Y, Ockene IS, Rosal MC, Merriam PA, Ockene JK, Gandhi PJ. Randomized Trial of a Pharmacist-Delivered Intervention for Improving Lipid-Lowering Medication Adherence among Patients with Coronary Heart Disease. Cholesterol. 2010;2010:383281.
303. Macintosh PW, Pond GR, Pond BJ, Leung V, Siu LL. A comparison of patient adherence and preference of packaging method for oral anticancer agents using conventional pill bottles versus daily pill boxes. Eur J Cancer Care (Engl). 2007;16(4):380-386.
304. Mackenzie G, Ireland S, Moore S, et al. Tailored interventions to improve hypertension management after stroke or TIA--phase II (TIMS II). Can J Neurosci Nurs. 2013;35(1):27-34.
305. Maduka O, Tobin-West CI. Adherence counseling and reminder text messages improve uptake of antiretroviral therapy in a tertiary hospital in Nigeria. Niger J Clin Pract. 2013;16(3):302-308.
306. Magid DJ, Ho PM, Olson KL, et al. A multimodal blood pressure control intervention in 3 healthcare systems. Am J Manag Care. 2011;17(4):e96-103.
307. Malotte CK, Hollingshead JR, Larro M. Incentives vs outreach workers for latent tuberculosis treatment in drug users. Am J Prev Med. 2001;20(2):103-107.
308. Maneesriwongul W, Prajanket O-O, Saengcharnchai P. Effects of motivational interviewing or an educational video on knowledge about HIV/AIDS, health beliefs and antiretroviral medication adherence among adult Thais with HIV/AIDS. Pacific Rim International Journal of Nursing Research. 2012;16(2):124-137.
309. Margolin A, Avants SK, Warburton LA, Hawkins KA, Shi J. A randomized clinical trial of a manual-guided risk reduction intervention for HIV-positive injection drug users. Health Psychol. 2003;22(2):223-228.
310. Markopoulos C, Neven P, Tanner M, Marty M, Kreienberg R, Atkins L, et al. Does patient education work in breast cancer? Final results from the global CARIATIDE study. Future oncology (London, England). 2015;11(2):205-17.
311. Marquez Contreras E, Casado Martinez JJ, Corchado Albalat Y, et al. [Efficacy of an intervention to improve treatment compliance in hyperlipidemias]. Aten Primaria. 2004;33(8):443-450.
312. Marquez Contreras E, Casado Martinez JJ, Corchado Albalat Y, et al. [Efficacy of an intervention to improve treatment compliance in hyperlipidemias]. Aten Primaria. 2004;33(8):443-450.
313. Márquez Contreras E, Joaquín Casado Martínez J, Motero Carrasco J, et al. El cumplimiento terapéutico en las dislipemias medido mediante monitores electrónicos. ¿Es eficaz un calendario recordatorio para evitar los olvidos? Atención Primaria. 2007;39(12):661-668.
314. Marquez Contreras E, Vegazo Garcia O, Martel Claros N, et al. Efficacy of telephone and mail intervention in patient compliance with antihypertensive drugs in hypertension. ETECUM-HTA study. Blood Press. 2005;14(3):151-158.
315. Martinot JB, Carr WD, Cullen S, et al. A comparative study of clarithromycin modified release and amoxicillin/clavulanic acid in the treatment of acute exacerbation of chronic bronchitis. Adv Ther. 2001;18(1):1-11.
316. Matsumura K, Arima H, Tominaga M, et al. Does a combination pill of antihypertensive drugs improve medication adherence in Japanese? A randomized controlled trial. Circ J. 2012;76(6):1415-1422.
317. Mbuagbaw L, Thabane L, Ongolo-Zogo P, et al. The Cameroon Mobile Phone SMS (CAMPS) trial: a randomized trial of text messaging versus usual care for adherence to antiretroviral therapy. PLoS One. 2012;7(12):e46909.
318. McGillicuddy JW, Gregoski MJ, Weiland AK, et al. Mobile Health Medication Adherence and Blood Pressure Control in Renal Transplant Recipients: A Proof-of-Concept Randomized Controlled Trial. JMIR Res Protoc. 2013;2(2):e32.
319. McKenney JM, Brown DE, Necsary R, Reavis HL. Effect of pharmacist drug monitoring and patient education on hypertensive patients. Contemporary Pharmacy Practice. 1978;1(2):50-56.
320. McKenney JM, Munroe WP, Wright JT, Jr. Impact of an electronic medication compliance aid on long-term blood pressure control. J Clin Pharmacol. 1992;32(3):277-283.
321. McKenney JM, Slining JM, Henderson HR, Devins D, Barr M. The effect of clinical pharmacy services on patients with essential hypertension. Circulation. 1973;48(5):1104-1111.
322. McKinstry B, Hanley J, Wild S, et al. Telemonitoring based service redesign for the management of uncontrolled hypertension: multicentre randomised controlled trial. BMJ. 2013;346:f3030.
323. McPherson-Baker S, Malow RM, Penedo F, Jones DL, Schneiderman N, Klimas NG. Enhancing adherence to combination antiretroviral therapy in non-adherent HIV-positive men. AIDS Care. 2000;12(4):399-404.
324. Mehos BM, Saseen JJ, MacLaughlin EJ. Effect of pharmacist intervention and initiation of home blood pressure monitoring in patients with uncontrolled hypertension. Pharmacotherapy. 2000;20(11):1384-1389.
325. Mehuys E, Van Bortel L, De Bolle L, et al. Effectiveness of pharmacist intervention for asthma control improvement. Eur Respir J. 2008;31(4):790-799.
326. Migneault JP, Dedier JJ, Wright JA, et al. A culturally adapted telecommunication system to improve physical activity, diet quality, and medication adherence among hypertensive African-Americans: a randomized controlled trial. Ann Behav Med. 2012;43(1):62-73.
327. Miklowitz DJ, Simoneau TL, George EL, et al. Family-focused treatment of bipolar disorder: 1-year effects of a psychoeducational program in conjunction with pharmacotherapy. Biol Psychiatry. 2000;48(6):582-592.
328. Miller P, Wikoff R, Garrett MJ, McMahon M, Smith T. Regimen compliance two years after myocardial infarction. Nurs Res. 1990;39(6):333-336.
329. Molina JM, Podsadecki TJ, Johnson MA, et al. A lopinavir/ritonavir-based once-daily regimen results in better compliance and is non-inferior to a twice-daily regimen through 96 weeks. AIDS Res Hum Retroviruses. 2007;23(12):1505-1514.
330. Mols RE, Jensen JM, Sand NP, et al. Visualization of Coronary Artery Calcification: Influence on Risk Modification. Am J Med. 2015;128(9):1023 e1023-1031.
331. Montes JM, Medina E, Gomez-Beneyto M, Maurino J. A short message service (SMS)-based strategy for enhancing adherence to antipsychotic medication in schizophrenia. Psychiatry Res. 2012;200(2-3):89-95.
332. Montori VM, Shah ND, Pencille LJ, et al. Use of a decision aid to improve treatment decisions in osteoporosis: the osteoporosis choice randomized trial. Am J Med. 2011;124(6):549-556.
333. Mooney M, Babb D, Jensen J, Hatsukami D. Interventions to increase use of nicotine gum: a randomized, controlled, single-blind trial. Nicotine Tob Res. 2005;7(4):565-579.
334. Mooney ME, Sayre SL, Hokanson PS, Stotts AL, Schmitz JM. Adding MEMS feedback to behavioral smoking cessation therapy increases compliance with bupropion: a replication and extension study. Addict Behav. 2007;32(4):875-880.
335. Moore JM, Shartle D, Faudskar L, Matlin OS, Brennan TA. Impact of a patient-centered pharmacy program and intervention in a high-risk group. J Manag Care Pharm. 2013;19(3):228-236.
336. Morales Suarez-Varela MT. [Study on the use of a smart pillbox to improve treatment compliance]. Aten Primaria. 2009;41(4):185-191.
337. Morgado M, Rolo S, Castelo-Branco M. Pharmacist intervention program to enhance hypertension control: a randomised controlled trial. Int J Clin Pharm. 2011;33(1):132-140.
338. Morisky DE, DeMuth NM, Field-Fass M, Green LW, Levine DM. Evaluation of family health education to build social support for long-term control of high blood pressure. Health Educ Q. 1985;12(1):35-50.
339. Morisky DE, Malotte CK, Choi P, et al. A patient education program to improve adherence rates with antituberculosis drug regimens. Health Educ Q. 1990;17(3):253-267.
340. Moshkovska T, Stone MA, Smith RM, Bankart J, Baker R, Mayberry JF. Impact of a tailored patient preference intervention in adherence to 5-aminosalicylic acid medication in ulcerative colitis: results from an exploratory randomized controlled trial. Inflamm Bowel Dis. 2011;17(9):1874-1881.
341. Moss AC, Chaudhary N, Tukey M, et al. Impact of a patient-support program on mesalamine adherence in patients with ulcerative colitis--a prospective study. J Crohns Colitis. 2010;4(2):171-175.
342. Mugusi F, Mugusi S, Bakari M, et al. Enhancing adherence to antiretroviral therapy at the HIV clinic in resource constrained countries; the Tanzanian experience. Trop Med Int Health. 2009;14(10):1226-1232.
343. Muir KW, Ventura A, Stinnett SS, Enfiedjian A, Allingham RR, Lee PP. The influence of health literacy level on an educational intervention to improve glaucoma medication adherence. Patient Educ Couns. 2012;87(2):160-164.
344. Munoz M, Finnegan K, Zeladita J, et al. Community-based DOT-HAART accompaniment in an urban resource-poor setting. AIDS Behav. 2010;14(3):721-730.
345. Murphy DA, Lu MC, Martin D, Hoffman D, Marelich WD. Results of a pilot intervention trial to improve antiretroviral adherence among HIV-positive patients. J Assoc Nurses AIDS Care. 2002;13(6):57-69.
346. Murphy DA, Marelich WD, Rappaport NB, Hoffman D, Farthing C. Results of an Antiretroviral Adherence Intervention: STAR (Staying Healthy: Taking Antiretrovirals Regularly). J Int Assoc Physicians AIDS Care (Chic). 2007;6(2):113-124.
347. Murray MD, Birt JA, Manatunga AK, Darnell JC. Medication compliance in elderly outpatients using twice-daily dosing and unit-of-use packaging. Ann Pharmacother. 1993;27(5):616-621.
348. Murray MD, Young J, Hoke S, et al. Pharmacist intervention to improve medication adherence in heart failure: a randomized trial. Ann Intern Med. 2007;146(10):714-725.
349. Nachega JB, Chaisson RE, Goliath R, et al. Randomized controlled trial of trained patient-nominated treatment supporters providing partial directly observed antiretroviral therapy. AIDS. 2010;24(9):1273-1280.
350. Najafi SS, Shaabani M, Momennassab M, Aghasadeghi K. The Nurse-Led Telephone Follow-Up on Medication and Dietary Adherence among Patients after Myocardial Infarction: A Randomized Controlled Clinical Trial. International journal of community based nursing and midwifery. 2016;4(3):199-208.
351. Nance N, Pendo P, Masanja J, Ngilangwa DP, Webb K, Noronha R, et al. Short-term effectiveness of a community health worker intervention for HIV-infected pregnant women in Tanzania to improve treatment adherence and retention in care: A cluster-randomized trial. PloS one. 2017;12(8):e0181919.
352. Nazareth I, Burton A, Shulman S, Smith P, Haines A, Timberal H. A pharmacy discharge plan for hospitalized elderly patients--a randomized controlled trial. Age Ageing. 2001;30(1):33-40.
353. Nesari M, Zakerimoghadam M, Rajab A, Bassampour S, Faghihzadeh S. Effect of telephone follow-up on adherence to a diabetes therapeutic regimen. Jpn J Nurs Sci. 2010;7(2):121-128.
354. Nessman DG, Carnahan JE, Nugent CA. Increasing compliance. Patient-operated hypertension groups. Arch Intern Med. 1980;140(11):1427-1430.
355. Nielsen D, Ryg J, Nielsen W, Knold B, Nissen N, Brixen K. Patient education in groups increases knowledge of osteoporosis and adherence to treatment: a two-year randomized controlled trial. Patient Educ Couns. 2010;81(2):155-160.
356. Nieuwkerk PT, Nierman MC, Vissers MN, et al. Intervention to improve adherence to lipid-lowering medication and lipid-levels in patients with an increased cardiovascular risk. Am J Cardiol. 2012;110(5):666-672.
357. Nollen NL, Cox LS, Nazir N, et al. A pilot clinical trial of varenicline for smoking cessation in black smokers. Nicotine Tob Res. 2011;13(9):868-873.
358. Nunes EV, Rothenberg JL, Sullivan MA, Carpenter KM, Kleber HD. Behavioral therapy to augment oral naltrexone for opioid dependence: a ceiling on effectiveness? Am J Drug Alcohol Abuse. 2006;32(4):503-517.
359. O'Carroll RE, Chambers JA, Dennis M, Sudlow C, Johnston M. Improving adherence to medication in stroke survivors: a pilot randomised controlled trial. Ann Behav Med. 2013;46(3):358-368.
360. O'Connor PJ, Schmittdiel JA, Pathak RD, et al. Randomized trial of telephone outreach to improve medication adherence and metabolic control in adults with diabetes. Diabetes Care. 2014;37(12):3317-3324.
361. Odegard PS, Christensen DB. MAP study: RCT of a medication adherence program for patients with type 2 diabetes. J Am Pharm Assoc (2003). 2012;52(6):753-762.
362. Ogedegbe G, Chaplin W, Schoenthaler A, et al. A practice-based trial of motivational interviewing and adherence in hypertensive African Americans. Am J Hypertens. 2008;21(10):1137-1143.
363. Ogedegbe G, Tobin JN, Fernandez S, et al. Counseling African Americans to Control Hypertension (CAATCH): Cluster Randomized Clinical Trial Main Effects. Circulation. 2014;129(20):2044-2051.
364. Ogedegbe GO, Boutin-Foster C, Wells MT, et al. A randomized controlled trial of positive-affect intervention and medication adherence in hypertensive African Americans. Arch Intern Med. 2012;172(4):322-326.
365. Okeke CO, Quigley HA, Jampel HD, et al. Interventions improve poor adherence with once daily glaucoma medications in electronically monitored patients. Ophthalmology. 2009;116(12):2286-2293.
366. Oliver S, Jones J, Leonard D, Crabbe A, Delkhah Y, Nesbitt S. Improving adherence with amlodipine/atorvastatin therapy: IMPACT study. J Clin Hypertens (Greenwich). 2011;13(8):598-604.
367. Ollivier L, Romand O, Marimoutou C, et al. Use of short message service (SMS) to improve malaria chemoprophylaxis compliance after returning from a malaria endemic area. Malar J. 2009;8:236.
368. Onyirimba F, Apter A, Reisine S, et al. Direct clinician-to-patient feedback discussion of inhaled steroid use: its effect on adherence. Ann Allergy Asthma Immunol. 2003;90(4):411-415.
369. Östbring MJ, Eriksson T, Petersson G, Hellström L. Medication beliefs and self-reported adherence–results of a pharmacist's consultation: a pilot study. European Journal of Hospital Pharmacy: Science and Practice. 2014;21(2):102-107.
370. Pagoto SL, McDermott MM, Reed G, et al. Can attention control conditions have detrimental effects on behavioral medicine randomized trials? Psychosom Med. 2013;75(2):137-143.
371. Palacio AM, Uribe C, Hazel-Fernandez L, et al. Can phone-based motivational interviewing improve medication adherence to antiplatelet medications after a coronary stent among racial minorities? A randomized trial. J Gen Intern Med. 2015;30(4):469-475.
372. Park DC, Morrell RW, Frieske D, Kincaid D. Medication adherence behaviors in older adults: effects of external cognitive supports. Psychol Aging. 1992;7(2):252-256.
373. Park JJ, Kelly P, Carter BL, Burgess PP. Comprehensive pharmaceutical care in the chain setting. J Am Pharm Assoc (Wash). 1996;NS36(7):443-451.
374. Park LG, Howie-Esquivel J, Chung ML, Dracup K. A text messaging intervention to promote medication adherence for patients with coronary heart disease: a randomized controlled trial. Patient Educ Couns. 2014;94(2):261-268.
375. Park YH, Chang H, Kim J, Kwak JS. Patient-tailored self-management intervention for older adults with hypertension in a nursing home. J Clin Nurs. 2013;22(5-6):710-722.
376. Parsons JT, Golub SA, Rosof E, Holder C. Motivational interviewing and cognitive-behavioral intervention to improve HIV medication adherence among hazardous drinkers: a randomized controlled trial. J Acquir Immune Defic Syndr. 2007;46(4):443-450.
377. Pearson CR, Micek MA, Simoni JM, et al. Randomized control trial of peer-delivered, modified directly observed therapy for HAART in Mozambique. J Acquir Immune Defic Syndr. 2007;46(2):238-244.
378. Peltzer K, Ramlagan S, Jones D, Weiss SM, Fomundam H, Chanetsa L. Efficacy of a lay health worker led group antiretroviral medication adherence training among non-adherent HIV-positive patients in KwaZulu-Natal, South Africa: results from a randomized trial. SAHARA J. 2012;9(4):218-226.
379. Peterson GM, McLean S, Millingen KS. A randomised trial of strategies to improve patient compliance with anticonvulsant therapy. Epilepsia. 1984;25(4):412-417.
380. Petrie KJ, Perry K, Broadbent E, Weinman J. A text message programme designed to modify patients' illness and treatment beliefs improves self-reported adherence to asthma preventer medication. Br J Health Psychol. 2012;17(1):74-84.
381. Piette JD, Weinberger M, Kraemer FB, McPhee SJ. Impact of automated calls with nurse follow-up on diabetes treatment outcomes in a Department of Veterans Affairs Health Care System: a randomized controlled trial. Diabetes Care. 2001;24(2):202-208.
382. Piette JD, Weinberger M, McPhee SJ, Mah CA, Kraemer FB, Crapo LM. Do automated calls with nurse follow-up improve self-care and glycemic control among vulnerable patients with diabetes? Am J Med. 2000;108(1):20-27.
383. Pladevall M, Brotons C, Gabriel R, et al. Multicenter cluster-randomized trial of a multifactorial intervention to improve antihypertensive medication adherence and blood pressure control among patients at high cardiovascular risk (the COM99 study). Circulation. 2010;122(12):1183-1191.
384. Pladevall M, Divine G, Wells KE, Resnicow K, Williams LK. A randomized controlled trial to provide adherence information and motivational interviewing to improve diabetes and lipid control. Diabetes Educ. 2015;41(1):136-146.
385. Planas LG, Crosby KM, Mitchell KD, Farmer KC. Evaluation of a hypertension medication therapy management program in patients with diabetes. J Am Pharm Assoc (2003). 2009;49(2):164-170.
386. Polack J, Jorgenson D, Robertson P. Evaluation of Different Methods of Providing Medication-Related Education to Patients following Myocardial Infarction. Canadian Pharmacists Journal / Revue des Pharmaciens du Canada. 2008;141(4):241-247.
387. Polsook R, Aungsuroch Y, Thongvichean T. The effect of self-efficacy enhancement program on medication adherence among post-acute myocardial infarction. Applied nursing research : ANR. 2016;32:67-72.
388. Pop-Eleches C, Thirumurthy H, Habyarimana JP, et al. Mobile phone technologies improve adherence to antiretroviral treatment in a resource-limited setting: a randomized controlled trial of text message reminders. AIDS. 2011;25(6):825-834.
389. Portsmouth SD, Osorio J, McCormick K, Gazzard BG, Moyle GJ. Better maintained adherence on switching from twice-daily to once-daily therapy for HIV: a 24-week randomized trial of treatment simplification using stavudine prolonged-release capsules. HIV Med. 2005;6(3):185-190.
390. Powell KM, Edgren B. Failure of educational videotapes to improve medication compliance in a health maintenance organization. Am J Health Syst Pharm. 1995;52(20):2196-2199.
391. Pradier C, Bentz L, Spire B, et al. Efficacy of an educational and counseling intervention on adherence to highly active antiretroviral therapy: French prospective controlled study. HIV Clin Trials. 2003;4(2):121-131.
392. Pullar T, Birtwell AJ, Wiles PG, Hay A, Feely MP. Use of a pharmacologic indicator to compare compliance with tablets prescribed to be taken once, twice, or three times daily. Clin Pharmacol Ther. 1988;44(5):540-545.
393. Purcell DW, Latka MH, Metsch LR, et al. Results from a randomized controlled trial of a peer-mentoring intervention to reduce HIV transmission and increase access to care and adherence to HIV medications among HIV-seropositive injection drug users. J Acquir Immune Defic Syndr. 2007;46 Suppl 2:S35-47.
394. Put C, van den Bergh O, Lemaigre V, Demedts M, Verleden G. Evaluation of an individualised asthma programme directed at behavioural change. Eur Respir J. 2003;21(1):109-115.
395. Pyne JM, Fortney JC, Curran GM, et al. Effectiveness of collaborative care for depression in human immunodeficiency virus clinics. Arch Intern Med. 2011;171(1):23-31.
396. Qureshi NN, Hatcher J, Chaturvedi N, Jafar TH. Effect of general practitioner education on adherence to antihypertensive drugs: cluster randomised controlled trial. BMJ. 2007;335(7628):1030.
397. Rabenda V, Mertens R, Fabri V, et al. Adherence to bisphosphonates therapy and hip fracture risk in osteoporotic women. Osteoporos Int. 2008;19(6):811-818.
398. Rabenda V, Vanoverloop J, Fabri V, et al. Low incidence of anti-osteoporosis treatment after hip fracture. J Bone Joint Surg Am. 2008;90(10):2142-2148.
399. Ramirez-Garcia P, Cote J. An individualized intervention to foster optimal antiretroviral treatment-taking behavior among persons living with HIV: a pilot randomized controlled trial. J Assoc Nurses AIDS Care. 2012;23(3):220-232.
400. Rathbun RC, Farmer KC, Stephens JR, Lockhart SM. Impact of an adherence clinic on behavioral outcomes and virologic response in treatment of HIV infection: a prospective, randomized, controlled pilot study. Clin Ther. 2005;27(2):199-209.
401. Rawlings MK, Thompson MA, Farthing CF, et al. Impact of an educational program on efficacy and adherence with a twice-daily lamivudine/zidovudine/abacavir regimen in underrepresented HIV-infected patients. J Acquir Immune Defic Syndr. 2003;34(2):174-183.
402. Raynor DK, Booth TG, Blenkinsopp A. Effects of computer generated reminder charts on patients' compliance with drug regimens. BMJ. 1993;306(6886):1158-1161.
403. Rea MM, Tompson MC, Miklowitz DJ, Goldstein MJ, Hwang S, Mintz J. Family-focused treatment versus individual treatment for bipolar disorder: results of a randomized clinical trial. J Consult Clin Psychol. 2003;71(3):482-492.
404. Rehder TL, McCoy LK, Blackwell B, Whitehead W, Robinson A. Improving medication compliance by counseling and special prescription container. Am J Hosp Pharm. 1980;37(3):379-385.
405. Reinares M, Colom F, Sanchez-Moreno J, et al. Impact of caregiver group psychoeducation on the course and outcome of bipolar patients in remission: a randomized controlled trial. Bipolar Disord. 2008;10(4):511-519.
406. Remien RH, Stirratt MJ, Dolezal C, et al. Couple-focused support to improve HIV medication adherence: a randomized controlled trial. AIDS. 2005;19(8):807-814.
407. Reynolds NR, Testa MA, Su M, et al. Telephone support to improve antiretroviral medication adherence: a multisite, randomized controlled trial. J Acquir Immune Defic Syndr. 2008;47(1):62-68.
408. Rich MW, Beckham V, Wittenberg C, Leven CL, Freedland KE, Carney RM. A multidisciplinary intervention to prevent the readmission of elderly patients with congestive heart failure. N Engl J Med. 1995;333(18):1190-1195.
409. Rich MW, Gray DB, Beckham V, Wittenberg C, Luther P. Effect of a multidisciplinary intervention on medication compliance in elderly patients with congestive heart failure. Am J Med. 1996;101(3):270-276.
410. Rickles NM, Svarstad BL, Statz-Paynter JL, Taylor LV, Kobak KA. Pharmacist telemonitoring of antidepressant use: effects on pharmacist-patient collaboration. J Am Pharm Assoc (2003). 2005;45(3):344-353.
411. Rigsby MO, Rosen MI, Beauvais JE, et al. Cue-dose training with monetary reinforcement: pilot study of an antiretroviral adherence intervention. J Gen Intern Med. 2000;15(12):841-847.
412. Rinfret S, Lussier MT, Peirce A, et al. The impact of a multidisciplinary information technology-supported program on blood pressure control in primary care. Circ Cardiovasc Qual Outcomes. 2009;2(3):170-177.
413. Rinfret S, Rodes-Cabau J, Bagur R, et al. Telephone contact to improve adherence to dual antiplatelet therapy after drug-eluting stent implantation. Heart. 2013;99(8):562-569.
414. Ringer J. Patient Persistency Project. JOURNALOFMANAGEDCAREPHARMAC~. 2001;7(1):50.
415. Robbins GK, Testa MA, Su M, et al. Site nurse-initiated adherence and symptom support telephone calls for HIV-positive individuals starting antiretroviral therapy, ACTG 5031: substudy of ACTG 384. HIV Clin Trials. 2013;14(5):235-253.
416. Robinson JD, Segal R, Lopez LM, Doty RE. Impact of a pharmaceutical care intervention on blood pressure control in a chain pharmacy practice. Ann Pharmacother. 2010;44(1):88-96.
417. Roden SM, Harvey PG, Mayer PP, Spence LI. Evaluation of two techniques to improve drug compliance in the elderly. Journal of Clinical & Experimental Gerontology. 1985;7(1):71-82.
418. Rosen MI, Dieckhaus K, McMahon TJ, et al. Improved adherence with contingency management. AIDS Patient Care STDS. 2007;21(1):30-40.
419. Rotheram-Borus MJ, Swendeman D, Comulada WS, Weiss RE, Lee M, Lightfoot M. Prevention for substance-using HIV-positive young people: telephone and in-person delivery. J Acquir Immune Defic Syndr. 2004;37 Suppl 2:S68-77.
420. Rozenfeld V, Pflomm J-M, Singh KK, Bazil MK, Cheng JWM. Assessing the Impact of Medication Consultations with a Medication Event Monitoring System. Hospital Pharmacy. 1999;34(5):539-550.
421. Rubio-Valera M, March Pujol M, Fernandez A, et al. Evaluation of a pharmacist intervention on patients initiating pharmacological treatment for depression: a randomized controlled superiority trial. Eur Neuropsychopharmacol. 2013;23(9):1057-1066.
422. Rudd P, Miller NH, Kaufman J, et al. Nurse management for hypertension. A systems approach. Am J Hypertens. 2004;17(10):921-927.
423. Ruiz I, Olry A, Lopez MA, Prada JL, Causse M. Prospective, randomized, two-arm controlled study to evaluate two interventions to improve adherence to antiretroviral therapy in Spain. Enferm Infecc Microbiol Clin. 2010;28(7):409-415.
424. Ruppar TM. Randomized pilot study of a behavioral feedback intervention to improve medication adherence in older adults with hypertension. J Cardiovasc Nurs. 2010;25(6):470-479.
425. Russell C, Conn V, Ashbaugh C, et al. Taking immunosuppressive medications effectively (TIMELink): a pilot randomized controlled trial in adult kidney transplant recipients. Clin Transplant. 2011;25(6):864-870.
426. Sabin LL, DeSilva MB, Hamer DH, et al. Using electronic drug monitor feedback to improve adherence to antiretroviral therapy among HIV-positive patients in China. AIDS Behav. 2010;14(3):580-589.
427. Sackett DL, Haynes RB, Gibson ES, et al. Randomised clinical trial of strategies for improving medication compliance in primary hypertension. Lancet. 1975;1(7918):1205-1207.
428. Sadik A, Yousif M, McElnay JC. Pharmaceutical care of patients with heart failure. Br J Clin Pharmacol. 2005;60(2):183-193.
429. Saini B, Filipovska J, Bosnic-Anticevich S, Taylor S, Krass I, Armour C. An evaluation of a community pharmacy-based rural asthma management service. The Australian journal of rural health. 2008;16(2):100-8.
430. Safren SA, Hendriksen ES, Desousa N, Boswell SL, Mayer KH. Use of an on-line pager system to increase adherence to antiretroviral medications. AIDS Care. 2003;15(6):787-793.
431. Safren SA, O'Cleirigh C, Tan JY, et al. A randomized controlled trial of cognitive behavioral therapy for adherence and depression (CBT-AD) in HIV-infected individuals. Health Psychol. 2009;28(1):1-10.
432. Safren SA, O'Cleirigh CM, Bullis JR, Otto MW, Stein MD, Pollack MH. Cognitive behavioral therapy for adherence and depression (CBT-AD) in HIV-infected injection drug users: a randomized controlled trial. J Consult Clin Psychol. 2012;80(3):404-415.
433. Safren SA, Otto MW, Worth JL, et al. Two strategies to increase adherence to HIV antiretroviral medication: life-steps and medication monitoring. Behav Res Ther. 2001;39(10):1151-1162.
434. Sajatovic M, Davies MA, Ganocy SJ, et al. A comparison of the life goals program and treatment as usual for individuals with bipolar disorder. Psychiatr Serv. 2009;60(9):1182-1189.
435. Saleem F, Hassali MA, Shafie AA, et al. Pharmacist intervention in improving hypertension-related knowledge, treatment medication adherence and health-related quality of life: a non-clinical randomized controlled trial. Health Expect. 2015;18(5):1270-1281.
436. Samet JH, Horton NJ, Meli S, et al. A randomized controlled trial to enhance antiretroviral therapy adherence in patients with a history of alcohol problems. Antivir Ther. 2005;10(1):83-93.
437. Sampaio-Sa M, Page-Shafer K, Bangsberg DR, et al. 100% adherence study: educational workshops vs. video sessions to improve adherence among ART-naive patients in Salvador, Brazil. AIDS and Behavior. 2008;12(1):54-62.
438. Sarna A, Luchters S, Geibel S, et al. Short- and long-term efficacy of modified directly observed antiretroviral treatment in Mombasa, Kenya: a randomized trial. J Acquir Immune Defic Syndr. 2008;48(5):611-619.
439. Saunders LD, Irwig LM, Gear JS, Ramushu DL. A randomized controlled trial of compliance improving strategies in Soweto hypertensives. Med Care. 1991;29(7):669-678.
440. Schaffer SD, Tian L. Promoting adherence: effects of theory-based asthma education. Clin Nurs Res. 2004;13(1):69-89.
441. Schectman G, Hiatt J, Hartz A. Telephone contacts do not improve adherence to niacin or bile acid sequestrant therapy. Ann Pharmacother. 1994;28(1):29-35.
442. Schmitz JM, Sayre SL, Stotts AL, Rothfleisch J, Mooney ME. Medication compliance during a smoking cessation clinical trial: a brief intervention using MEMS feedback. J Behav Med. 2005;28(2):139-147.
443. Schneider PJ, Murphy JE, Pedersen CA. Impact of medication packaging on adherence and treatment outcomes in older ambulatory patients. J Am Pharm Assoc (2003). 2008;48(1):58-63.
444. Schroeder K, Fahey T, Hollinghurst S, Peters TJ. Nurse-led adherence support in hypertension: a randomized controlled trial. Fam Pract. 2005;22(2):144-151.
445. Schulz M, Gray R, Spiekermann A, Abderhalden C, Behrens J, Driessen M. Adherence therapy following an acute episode of schizophrenia: a multi-centre randomised controlled trial. Schizophr Res. 2013;146(1-3):59-63.
446. Sclar DA, Chin A, Skaer TL, Okamoto MP, Nakahiro RK, Gill MA. Effect of health education in promoting prescription refill compliance among patients with hypertension. Clin Ther. 1991;13(4):489-495.
447. Selke HM, Kimaiyo S, Sidle JE, et al. Task-shifting of antiretroviral delivery from health care workers to persons living with HIV/AIDS: clinical outcomes of a community-based program in Kenya. J Acquir Immune Defic Syndr. 2010;55(4):483-490.
448. Sewerynek E, Horst-Sikorska H, Stepien-Klos W, et al. The role of counselling and other factors in compliance of postmenopausal osteoporotic patients to alendronate 70 therapy. Arch Med Sci. 2013;9(2):288-296.
449. Sherrard H, Struthers C, Kearns SA, Wells G, Chen L, Mesana T. Using technology to create a medication safety net for cardiac surgery patients: a nurse-led randomized control trial. Can J Cardiovasc Nurs. 2009;19(3):9-15.
450. Shet A, De Costa A, Kumarasamy N, et al. Effect of mobile telephone reminders on treatment outcome in HIV: evidence from a randomised controlled trial in India. BMJ. 2014;349:g5978.
451. Shu AD, Stedman MR, Polinski JM, et al. Adherence to osteoporosis medications after patient and physician brief education: post hoc analysis of a randomized controlled trial. Am J Manag Care. 2009;15(7):417-424.
452. Silva RCe, Pellanda L, Portal V, Maciel P, Furquim A, Schaan B. Transdiciplinary Approach to the Follow-Up of Patients After Myocardial Infarction. Clinics (Sao Paulo, Brazil). 2008;63(4):489-496.
453. Silveira MP, Guttier MC, Page K, Moreira LB. Randomized controlled trial to evaluate the impact of pharmaceutical care on therapeutic success in HIV-infected patients in Southern Brazil. AIDS Behav. 2014;18 Suppl 1:S75-84.
454. Simkins CV, Wenzloff NJ. Evaluation of a computerized reminder system in the enhancement of patient medication refill compliance. Drug Intell Clin Pharm. 1986;20(10):799-802.
455. Simon GE, Ralston JD, Savarino J, Pabiniak C, Wentzel C, Operskalski BH. Randomized trial of depression follow-up care by online messaging. J Gen Intern Med. 2011;26(7):698-704.
456. Simoni JM, Huh D, Frick PA, et al. Peer support and pager messaging to promote antiretroviral modifying therapy in Seattle: a randomized controlled trial. J Acquir Immune Defic Syndr. 2009;52(4):465-473.
457. Simoni JM, Pantalone DW, Plummer MD, Huang B. A randomized controlled trial of a peer support intervention targeting antiretroviral medication adherence and depressive symptomatology in HIV-positive men and women. Health Psychol. 2007;26(4):488-495.
458. Simoni JM, Wiebe JS, Sauceda JA, Huh D, Sanchez G, Longoria V, et al. A preliminary RCT of CBT-AD for adherence and depression among HIV-positive Latinos on the U.S.-Mexico border: the Nuevo Dia study. AIDS and behavior. 2013;17(8):2816-29.
459. Sit JW, Yip VY, Ko SK, Gun AP, Lee JS. A quasi-experimental study on a community-based stroke prevention programme for clients with minor stroke. Journal of clinical nursing. 2007;16(2):272-81.
460. Skaer TL, Sclar DA, Markowski DJ, Won JK. Effect of value-added utilities on prescription refill compliance and health care expenditures for hypertension. J Hum Hypertens. 1993;7(5):515-518.
461. Skaer TL, Sclar DA, Markowski DJ, Won JK. Effect of value-added utilities on prescription refill compliance and Medicaid health care expenditures--a study of patients with non-insulin-dependent diabetes mellitus. J Clin Pharm Ther. 1993;18(4):295-299.
462. Smith L, Bosnic-Anticevich SZ, Mitchell B, Saini B, Krass I, Armour C. Treating asthma with a self-management model of illness behaviour in an Australian community pharmacy setting. Social science & medicine (1982). 2007;64(7):1501-11.
463. Smith DH, Kramer JM, Perrin N, et al. A randomized trial of direct-to-patient communication to enhance adherence to beta-blocker therapy following myocardial infarction. Arch Intern Med. 2008;168(5):477-483; discussion 483; quiz 447.
464. Smith SR, Rublein JC, Marcus C, Brock TP, Chesney MA. A medication self-management program to improve adherence to HIV therapy regimens. Patient Educ Couns. 2003;50(2):187-199.
465. Solomon DH, Iversen MD, Avorn J, et al. Osteoporosis Telephonic Intervention to Improve Medication Adherence (OPTIMA): A Large Pragmatic Randomized Controlled Trial. Archives of internal medicine. 2012;172(6):477-483.
466. Solomon DH, Iversen MD, Avorn J, et al. Osteoporosis Telephonic Intervention to Improve Medication Adherence (OPTIMA): A Large Pragmatic Randomized Controlled Trial. Archives of internal medicine. 2012;172(6):477-483.
467. Solomon DK, Portner TS, Bass GE, et al. Clinical and economic outcomes in the hypertension and COPD arms of a multicenter outcomes study. J Am Pharm Assoc (Wash). 1998;38(5):574-585.
468. Sookaneknun P, Richards RM, Sanguansermsri J, Teerasut C. Pharmacist involvement in primary care improves hypertensive patient clinical outcomes. Ann Pharmacother. 2004;38(12):2023-2028.
469. Sorensen JL, Haug NA, Delucchi KL, et al. Voucher reinforcement improves medication adherence in HIV-positive methadone patients: a randomized trial. Drug Alcohol Depend. 2007;88(1):54-63.
470. Sosa N, Hill-Zabala C, Dejesus E, et al. Abacavir and lamivudine fixed-dose combination tablet once daily compared with abacavir and lamivudine twice daily in HIV-infected patients over 48 weeks (ESS30008, SEAL). J Acquir Immune Defic Syndr. 2005;40(4):422-427.
471. Stacy JN, Schwartz SM, Ershoff D, Shreve MS. Incorporating tailored interactive patient solutions using interactive voice response technology to improve statin adherence: results of a randomized clinical trial in a managed care setting. Popul Health Manag. 2009;12(5):241-254.
472. Staring AB, Van der Gaag M, Koopmans GT, et al. Treatment adherence therapy in people with psychotic disorders: randomised controlled trial. Br J Psychiatry. 2010;197(6):448-455.
473. Stewart K, George J, Mc Namara KP, et al. A multifaceted pharmacist intervention to improve antihypertensive adherence: a cluster-randomized, controlled trial (HAPPy trial). J Clin Pharm Ther. 2014;39(5):527-534.
474. Stewart K, Mc Namara KP, George J. Challenges in measuring medication adherence: experiences from a controlled trial. Int J Clin Pharm. 2014;36(1):15-19.
475. Strandbygaard U, Thomsen SF, Backer V. A daily SMS reminder increases adherence to asthma treatment: a three-month follow-up study. Respir Med. 2010;104(2):166-171.
476. Su WJ, Perng RP. Fixed-dose combination chemotherapy (Rifater/Rifinah) for active pulmonary tuberculosis in Taiwan: a two-year follow-up. Int J Tuberc Lung Dis. 2002;6(11):1029-1032.
477. Svarstad BL, Kotchen JM, Shireman TI, et al. Improving refill adherence and hypertension control in black patients: Wisconsin TEAM trial. J Am Pharm Assoc (2003). 2013;53(5):520-529.
478. Sweeney S, Dixon J, Sutcliffe I. The impact of the clinical pharmacist on compliance in a geriatric population. Pharm J. 1989;242:R4-6.
479. Taggart AJ, Johnston GD, McDevitt DG. Does the frequency of daily dosage influence compliance with digoxin therapy? Br J Clin Pharmacol. 1981;11(1):31-34.
480. Taiwo BO, Idoko JA, Welty LJ, et al. Assessing the viorologic and adherence benefits of patient-selected HIV treatment partners in a resource-limited setting. J Acquir Immune Defic Syndr. 2010;54(1):85-92.
481. Tan H, Yu J, Tabby D, Devries A, Singer J. Clinical and economic impact of a specialty care management program among patients with multiple sclerosis: a cohort study. Mult Scler. 2010;16(8):956-963.
482. Taylor CT, Byrd DC, Krueger K. Improving primary care in rural Alabama with a pharmacy initiative. Am J Health Syst Pharm. 2003;60(11):1123-1129.
483. Team CPMMPE. The MEDMAN study: a randomized controlled trial of community pharmacy-led medicines management for patients with coronary heart disease. Fam Pract. 2007;24(2):189-200.
484. Thom S, Poulter N, Field J, et al. Effects of a fixed-dose combination strategy on adherence and risk factors in patients with or at high risk of CVD: the UMPIRE randomized clinical trial. JAMA. 2013;310(9):918-929.
485. Tinsel I, Buchholz A, Vach W, et al. Shared decision-making in antihypertensive therapy: a cluster randomised controlled trial. BMC Fam Pract. 2013;14:135.
486. Tsuyuki RT, Fradette M, Johnson JA, et al. A multicenter disease management program for hospitalized patients with heart failure. J Card Fail. 2004;10(6):473-480.
487. Tuldra A, Fumaz CR, Ferrer MJ, et al. Prospective randomized two-Arm controlled study to determine the efficacy of a specific intervention to improve long-term adherence to highly active antiretroviral therapy. J Acquir Immune Defic Syndr. 2000;25(3):221-228.
488. Udelson JE, Pressler SJ, Sackner-Bernstein J, et al. Adherence with once daily versus twice daily carvedilol in patients with heart failure: the Compliance And Quality of Life Study Comparing Once-Daily Controlled-Release Carvedilol CR and Twice-Daily Immediate-Release Carvedilol IR in Patients with Heart Failure (CASPER) Trial. J Card Fail. 2009;15(5):385-393.
489. Uysal H, Ozcan S. The effect of individual education on patients' physical activity capacity after myocardial infarction. International journal of nursing practice. 2015;21(1):18-28.
490. Valencia M, Rascon ML, Juarez F, Murow E. A psychosocial skills training approach in Mexican out-patients with schizophrenia. Psychol Med. 2007;37(10):1393-1402.
491. van Gent EM, Zwart FM. Psychoeducation of partners of bipolar-manic patients. J Affect Disord. 1991;21(1):15-18.
492. van Onzenoort HA, Verberk WJ, Kroon AA, et al. Effect of self-measurement of blood pressure on adherence to treatment in patients with mild-to-moderate hypertension. J Hypertens. 2010;28(3):622-627.
493. van Servellen G, Carpio F, Lopez M, et al. Program to enhance health literacy and treatment adherence in low-income HIV-infected Latino men and women. AIDS Patient Care STDS. 2003;17(11):581-594.
494. van Servellen G, Nyamathi A, Carpio F, et al. Effects of a treatment adherence enhancement program on health literacy, patient-provider relationships, and adherence to HAART among low-income HIV-positive Spanish-speaking Latinos. AIDS Patient Care STDS. 2005;19(11):745-759.
495. Varma S, McElnay JC, Hughes CM, Passmore AP, Varma M. Pharmaceutical care of patients with congestive heart failure: interventions and outcomes. Pharmacotherapy. 1999;19(7):860-869.
496. Velligan DI, Diamond PM, Mintz J, et al. The use of individually tailored environmental supports to improve medication adherence and outcomes in schizophrenia. Schizophr Bull. 2008;34(3):483-493.
497. Vervloet M, van Dijk L, Santen-Reestman J, et al. SMS reminders improve adherence to oral medication in type 2 diabetes patients who are real time electronically monitored. Int J Med Inform. 2012;81(9):594-604.
498. Villeneuve J, Genest J, Blais L, et al. A cluster randomized controlled Trial to Evaluate an Ambulatory primary care Management program for patients with dyslipidemia: the TEAM study. CMAJ. 2010;182(5):447-455.
499. Vollmer WM, Feldstein A, Smith DH, Dubanoski JP, Waterbury A, Schneider JL, et al. Use of health information technology to improve medication adherence. The American journal of managed care. 2011;17(12 Spec No.):Sp79-87.
500. Vollmer WM, Owen-Smith AA, Tom JO, et al. Improving adherence to cardiovascular disease medications with information technology. Am J Manag Care. 2014;20(11 Spec No. 17):SP502-510.
501. Volpp KG, Loewenstein G, Troxel AB, et al. A test of financial incentives to improve warfarin adherence. BMC Health Serv Res. 2008;8:272.
502. Vrijens B, Belmans A, Matthys K, de Klerk E, Lesaffre E. Effect of intervention through a pharmaceutical care program on patient adherence with prescribed once-daily atorvastatin. Pharmacoepidemiol Drug Saf. 2006;15(2):115-121.
503. Wagner GJ, Kanouse DE, Golinelli D, et al. Cognitive-behavioral intervention to enhance adherence to antiretroviral therapy: a randomized controlled trial (CCTG 578). AIDS. 2006;20(9):1295-1302.
504. Wagner GJ, Lovely P, Schneider S. Pilot controlled trial of the adherence readiness program: an intervention to assess and sustain HIV antiretroviral adherence readiness. AIDS Behav. 2013;17(9):3059-3065.
505. Wakefield BJ, Holman JE, Ray A, et al. Outcomes of a home telehealth intervention for patients with diabetes and hypertension. Telemed J E Health. 2012;18(8):575-579.
506. Wakefield BJ, Holman JE, Ray A, et al. Outcomes of a home telehealth intervention for patients with heart failure. J Telemed Telecare. 2009;15(1):46-50.
507. Wald DS, Bestwick JP, Raiman L, Brendell R, Wald NJ. Randomised trial of text messaging on adherence to cardiovascular preventive treatment (INTERACT trial). PLoS One. 2014;9(12):e114268.
508. Walker CC. An educational intervention for hypertension management in older African Americans. Ethn Dis. 2000;10(2):165-174.
509. Wall TL, Sorensen JL, Batki SL, Delucchi KL, London JA, Chesney MA. Adherence to zidovudine (AZT) among HIV-infected methadone patients: a pilot study of supervised therapy and dispensing compared to usual care. Drug Alcohol Depend. 1995;37(3):261-269.
510. Wan LH, Zhang XP, Mo MM, Xiong XN, Ou CL, You LM, et al. Effectiveness of Goal-Setting Telephone Follow-Up on Health Behaviors of Patients with Ischemic Stroke: A Randomized Controlled Trial. Journal of stroke and cerebrovascular diseases : the official journal of National Stroke Association. 2016;25(9):2259-70.
511. Wang KY, Chian CF, Lai HR, Tarn YH, Wu CP. Clinical pharmacist counseling improves outcomes for Taiwanese asthma patients. Pharmacy world & science : PWS. 2010;32(6):721-9.
512. Wang H, Zhou J, Huang L, Li X, Fennie KP, Williams AB. Effects of nurse-delivered home visits combined with telephone calls on medication adherence and quality of life in HIV-infected heroin users in Hunan of China. J Clin Nurs. 2010;19(3-4):380-388.
513. Wang J, Wu J, Yang J, et al. Effects of pharmaceutical care interventions on blood pressure and medication adherence of patients with primary hypertension in China. Clinical Research and Regulatory Affairs. 2011;28(1):1-6.
514. Wang K, Wang C, Xi L, et al. A randomized controlled trial to assess adherence to allergic rhinitis treatment following a daily short message service (SMS) via the mobile phone. Int Arch Allergy Immunol. 2014;163(1):51-58.
515. Webb PA. Effectiveness of patient education and psychosocial counseling in promoting compliance and control among hypertensive patients. J Fam Pract. 1980;10(6):1047-1055.
516. Weber R, Christen L, Christen S, et al. Effect of individual cognitive behaviour intervention on adherence to antiretroviral therapy: prospective randomized trial. Antivir Ther. 2004;9(1):85-95.
517. Weinberger M, Tierney WM, Booher P, Katz BP. The impact of increased contact on psychosocial outcomes in patients with osteoarthritis: a randomized, controlled trial. J Rheumatol. 1991;18(6):849-854.
518. Williams A, Manias E, Walker R, Gorelik A. A multifactorial intervention to improve blood pressure control in co-existing diabetes and kidney disease: a feasibility randomized controlled trial. J Adv Nurs. 2012;68(11):2515-2525.
519. Williams AB, Fennie KP, Bova CA, Burgess JD, Danvers KA, Dieckhaus KD. Home visits to improve adherence to highly active antiretroviral therapy: a randomized controlled trial. J Acquir Immune Defic Syndr. 2006;42(3):314-321.
520. Williams AB, Wang H, Li X, Chen J, Li L, Fennie K. Efficacy of an evidence-based ARV adherence intervention in China. AIDS Patient Care STDS. 2014;28(8):411-417.
521. Windsor RA, Bailey WC, Richards JM, Jr., Manzella B, Soong SJ, Brooks M. Evaluation of the efficacy and cost effectiveness of health education methods to increase medication adherence among adults with asthma. Am J Public Health. 1990;80(12):1519-1521.
522. Winland-Brown JE, Valiante J. Effectiveness of different medication management approaches on elders' medication adherence. Outcomes Manag Nurs Pract. 2000;4(4):172-176.
523. Wohl AR, Garland WH, Valencia R, et al. A randomized trial of directly administered antiretroviral therapy and adherence case management intervention. Clin Infect Dis. 2006;42(11):1619-1627.
524. Wong LY, Chua SS, Husin AR, Arshad H. A pharmacy management service for adults with asthma: a cluster randomised controlled trial. Family practice. 2017;34(5):564-73.
525. Wong MC, Liu KQ, Wang HH, et al. Effectiveness of a pharmacist-led drug counseling on enhancing antihypertensive adherence and blood pressure control: a randomized controlled trial. J Clin Pharmacol. 2013;53(7):753-761.
526. Wright JM, Htun Y, Leong MG, Forman P, Ballard RC. Evaluation of the use of calendar blister packaging on patient compliance with STD syndromic treatment regimens. Sex Transm Dis. 1999;26(10):556-563.
527. Wu JR, Corley DJ, Lennie TA, Moser DK. Effect of a medication-taking behavior feedback theory-based intervention on outcomes in patients with heart failure. J Card Fail. 2012;18(1):1-9.
528. Wyatt GE, Longshore D, Chin D, et al. The efficacy of an integrated risk reduction intervention for HIV-positive women with child sexual abuse histories. AIDS Behav. 2004;8(4):453-462.
529. Xavier D, Gupta R, Kamath D, Sigamani A, Devereaux PJ, George N, et al. Community health worker-based intervention for adherence to drugs and lifestyle change after acute coronary syndrome: a multicentre, open, randomised controlled trial. The lancet Diabetes & endocrinology. 2016;4(3):244-53.
530. Young H, Nadra Havican S, A Chewning B, A Sorkness C, Ruppel X, Griesbach S. Patient And phaRmacit Telephonic Encounters (PARTE) in an Underserved Rural Population with Asthma: Methods and Rationale2011. 49 p.
531. Zang XY, Liu JF, Chai YF, Wong FK, Zhao Y. Effect on blood pressure of a continued nursing intervention using chronotherapeutics for adult Chinese hypertensive patients. J Clin Nurs. 2010;19(7-8):1149-1156.
532. Zaretsky A, Lancee W, Miller C, Harris A, Parikh SV. Is cognitive-behavioural therapy more effective than psychoeducation in bipolar disorder? Can J Psychiatry. 2008;53(7):441-448.
533. Zarnke KB, Feagan BG, Mahon JL, Feldman RD. A randomized study comparing a patient-directed hypertension management strategy with usual office-based care. Am J Hypertens. 1997;10(1):58-67.
534. Ziller V, Kyvernitakis I, Knoll D, Storch A, Hars O, Hadji P. Influence of a patient information program on adherence and persistence with an aromatase inhibitor in breast cancer treatment--the COMPAS study. BMC cancer. 2013;13:407.
535. Zillich AJ, Jaynes HA, Snyder ME, et al. Evaluation of specialized medication packaging combined with medication therapy management: adherence, outcomes, and costs among Medicaid patients. Med Care. 2012;50(6):485-493.
536. Zillich AJ, Sutherland JM, Kumbera PA, Carter BL. Hypertension outcomes through blood pressure monitoring and evaluation by pharmacists (HOME study). J Gen Intern Med. 2005;20(12):1091-1096.
537. Zwikker HE, van den Ende CH, van Lankveld WG, et al. Effectiveness of a group-based intervention to change medication beliefs and improve medication adherence in patients with rheumatoid arthritis: a randomized controlled trial. Patient Educ Couns. 2014;94(3):356-361.
     1. **Excluded primary studies**

| **Original studies** | **Excluded** |
| --- | --- |
| Quilici, 2013 | Non-experimental study |
| Gould, 2011 | Non-experimental study |
| Simoni, 2011 | Undefined/excluded outcomes |
| Berger, 2003 | Undefined/excluded outcomes |
| Awofeso, 1995 | Non-experimental study |
| Becker, 1986 | Undefined/excluded outcomes |
| Kennedy, 1990 | Non-experimental study |
| Levensky, 2006 | Non-experimental study |
| MacDonald, 1977 | Undefined/excluded outcomes |
| Qingjun, 1998a | Included pediatrics |
| Revankar, 1993 | Included pediatrics |
| Spriet, 1980 | Non-experimental study |
| Traiger, 1997 | Undefined/excluded outcomes |
| Hosie, 1995 | Undefined/excluded outcomes |
| Lennon, 2008 | Included pediatrics |
| Adam, 2001 | Included pediatrics |
| Garcia Callejo, 1998 | Included pediatrics |
| Clegg, 2006 | Included pediatrics |
| Ballantyne, 1985 | Out of scope |
| Linder, 1993 | Included pediatrics |
| Gooch, 1997 | Included pediatrics |
| Edelstein, 1993 | Included pediatrics |
| Owen, 1993 | Included pediatrics |
| Venuta, 1998 | Included pediatrics |
| Disney, 1990 | Included pediatrics |
| Mita, 2003 | Included pediatrics |
| Cohen, 1996 | Included pediatrics |
| Behre, 1997 | Included pediatrics |
| Pichichero, 1999 | Included pediatrics |
| Gooch, 1993 | Included pediatrics |
| Richard, 1981 | Included pediatrics |
| Damrikarnlert, 2000 | Included pediatrics |
| Gerber, 1985 | Included pediatrics |
| Gehanno, 1994 | Included pediatrics |
| Cook, 1996 | Included pediatrics |
| Dawson, 1998 | Non-experimental study |
| Nimpitakpong, 2002 | Non-experimental study |
| Nucifora, 2006 | Undefined/excluded outcomes |
| Powell, 2010 | Undefined/excluded outcomes |
| Tierney, 2003 | Non patient-targeted interventions |
| Clarkin, 1998 | Out of scope |
| Colom, 2003 | Undefined/excluded outcomes |
| Lenz, 2010 | Non-experimental study |
| Maneesakorn, 2007 | Undefined/excluded outcomes |
| von Bormann, 2015 | Undefined/excluded outcomes |
| Austin, 1986 | Non-experimental study |
| Cook, 2010 | Undefined/excluded outcomes |
| De Geest, 2006 | Undefined/excluded outcomes |
| Freedman, 2007 | Non-experimental study |
| Harper, 1984 | Undefined/excluded outcomes |
| Matterson, 2011 | Non-experimental study |
| Mitchell, 1992 | Non-experimental study |
| Moitra, 2011 | Undefined/excluded outcomes |
| Nietert, 2009 | Non patient-targeted interventions |
| Oser, 2008 | Non-experimental study |
| Stewart, 2008 | Non-experimental study |
| Watakakasol, 2010 | Non-experimental study |
| Wu, 2006 | Undefined/excluded outcomes |
| Zuckerman, 2004 | Non patient-targeted interventions |
| Christensen, 2010 | Undefined/excluded outcomes |
| Chung, 2011 | Undefined/excluded outcomes |
| Franklin, 2006 | Included pediatrics |
| Rothschild, 2014 | Undefined/excluded outcomes |
| Zolfaghari, 2012 | Non-experimental study |
| Walker, 2011 | Undefined/excluded outcomes |
| Benson, 2004 | Out of scope |
| Parienti, 2007 | Undefined/excluded outcomes |
| Rode, 2008 | Non-experimental study |
| Ruane, 2006 | Undefined/excluded outcomes |
| Tulsky, 2000 | Undefined/excluded outcomes |
| Bock, 2001 | Undefined/excluded outcomes |
| Morisky, 2001 | Undefined/excluded outcomes |
| Jakubowiak, 2007 | Undefined/excluded outcomes |
| Martins, 2009 | Undefined/excluded outcomes |
| Grabowski, 1979 | Undefined/excluded outcomes |
| Preston, 1999 | Undefined/excluded outcomes |
| Carroll, 2001 | Out of scope |
| Carroll, 2002 | Out of scope |
| Seal, 2003 | Excluded medication |
| Stitzer, 2010 | Excluded medication |
| Claassen, 2007 | Undefined/excluded outcomes |
| Berger, 2005 | Undefined/excluded outcomes |
| George, 2010 | Non-experimental study |
| Hovell, 2003 | Included pediatrics |
| Sheeran, 1999 | Included pediatrics |
| Van Es, 2001 | Included pediatrics |
| Engelbrecht, 2010 | Non-experimental study |
| Sabin, 2014 | Non-experimental study |
| Davies, 2010 | Non-experimental study |
| Wilson, 2010 | Non patient-targeted interventions |
| Adler, 2004 | Undefined/excluded outcomes |
| Lai, 2010 | Non-experimental study |
| Heilmann, 2012 | Out of scope |
| Ting, 2012 | Included pediatrics |
| Cook, 2010 | Non-experimental study |
| Stockl, 2010 | Out of scope |
| Stockl, 2010b | Out of scope |
| Tamone, 2012 | Non-experimental study |
| Downey, 2006 | Non-experimental study |
| Marteau, 2012 | Out of scope |
| Smith, 2013 | Excluded medication |
| Musser, 2001 | Non-experimental study |
| RCTAI, 1989 | Non-experimental study |
| Hulsbosch, 2008 | Non-experimental study |
| Jones, 2007 | Undefined/excluded outcomes |
| Tuldra, 2002 | Undefined/excluded outcomes |
| Maasland, 2007 | Undefined/excluded outcomes |
| Armstrong, 2009 | Excluded medication |
| Cococila, 2009 | Undefined/excluded outcomes |
| Yentzer, 2011 | Non-experimental study |
| Binstock, 1988 | Undefined/excluded outcomes |
| Huang, 2000 | Excluded medication |
| Jansen, 2009 | Undefined/excluded outcomes |
| Kripalani, 2007 | Undefined/excluded outcomes |
| Simmons, 2000 | Undefined/excluded outcomes |
| Suppapitiporn, 2005 | Undefined/excluded outcomes |
| Dezii, 2000 | Non-experimental study |
| NDC data set | Non-experimental study |
| Melikian, 2002 | Non-experimental study |
| Knobel, 1999 | Undefined/excluded outcomes |
| Kelly, 1988 | Undefined/excluded outcomes |
| Nicoleau, 1985 | Non-experimental study |
| Zhao, 2004 | Non-experimental study |
| Lourenco, 2011 | Non-experimental study |
| Campbell, 1998 | Undefined/excluded outcomes |
| Gould, 2011 | Non-experimental study |
| Lehr, 1986 | Non-experimental study |
| Muniz, 2010 | Undefined/excluded outcomes |
| Shemesh, 2006 | Undefined/excluded outcomes |
| Yilmaz, 2005 | Undefined/excluded outcomes |
| Anderson, 2004 | Out of scope |
| Austin, 1986 | Non-experimental study |
| Bogart, 2012 | Undefined/excluded outcomes |
| Freedman, 2007 | Non-experimental study |
| Harper, 1984 | Undefined/excluded outcomes |
| Laine, 1996 | Undefined/excluded outcomes |
| Mann, 2001 | Out of scope |
| Martin, 2011 | Undefined/excluded outcomes |
| Moitra, 2011 | Undefined/excluded outcomes |
| Mundy, 2009 | Non-experimental study |
| Newsome, 1995 | Non-experimental study |
| Powell, 2002 | Non-experimental study |
| Tagliacozzo, 1974 | Undefined/excluded outcomes |
| Vivian, 2002 | Undefined/excluded outcomes |
| Webel, 2010 | Undefined/excluded outcomes |
| Werner, 1979 | Non-experimental study |
| Fujii, 1985 | Non-experimental study |
| Eisen, 1990 | Non-experimental study |
| Halpern, 1993 | Excluded medication |
| Brun, 1994 | Non-experimental study |
| Halfmann, 2000 | Non-experimental study |
| Jennings, 1992 | Non-experimental study |
| Rimer, 1987 | Undefined/excluded outcomes |
| Wood, 1989 | Undefined/excluded outcomes |
| Eisen, 1990 | Non-experimental study |
| Girvin, 1999 | Non-experimental study |
| Berkovitch, 1998 | Included pediatrics |
| Rapoff, 2002 | Included pediatrics |
| Grosset, 2007 | Non-experimental study |
| Ducharme, 2011 | Included pediatrics |
| Bonner, 2002 | Included pediatrics |
| Canino, 2008 | Included pediatrics |
| Morisky, 2001 | Undefined/excluded outcomes |
| Schwartz-Lookinland, 1989 | Included pediatrics |
| Yin, 2008 | Included pediatrics |
| Armour, 2004 | Undefined/excluded outcomes |
| Berrien, 2004 | Included pediatrics |
| Bertakis, 1986 | Included pediatrics |
| Colcher, 1972 | Included pediatrics |
| Ellison, 1982 | Included pediatrics |
| Eriksson, 1998 | Out of scope |
| Finney, 1985 | Included pediatrics |
| Gibbs, 1989 | Undefined/excluded outcomes |
| Jameson, 1995 | Undefined/excluded outcomes |
| Levesque, 1983 | Out of scope |
| Linszen, 1996 | Included pediatrics |
| McCrindle, 1997 | Included pediatrics |
| Mengze, 1994 | Undefined/excluded outcomes |
| Sanmarti, 1993 | Included pediatrics |
| Sellors, 1997 | Undefined/excluded outcomes |
| Sharpe, 1974 | Undefined/excluded outcomes |
| Smith, 1986 | Included pediatrics |
| Spriet, 1980 | Non-experimental study |
| Tinkelman, 1980 | Included pediatrics |
| Williams, 1986 | Included pediatrics |
| Al Owaish, 1985 | Non-experimental study |
| Avanzini, 2002 | Non patient-targeted interventions |
| Beeson, 1977 | Non-experimental study |
| Cooper, 2011 | Non patient-targeted interventions |
| Evans, 1986 | Non patient-targeted interventions |
| Fletcher, 1975 | Undefined/excluded outcomes |
| Gomez-Marcos, 2006 | Non patient-targeted interventions |
| Harowski, 1983 | Non-experimental study |
| Harper, 1984 | Undefined/excluded outcomes |
| Hess, 2007 | Undefined/excluded outcomes |
| Inui, 1976 | Non patient-targeted interventions |
| Jafar, 2009 | Undefined/excluded outcomes |
| Kobalava, 2010 | Non-experimental study |
| Leung, 2012 | Non-experimental study |
| Magadza, 2009 | Non-experimental study |
| Martin, 2011 | Undefined/excluded outcomes |
| Muhlhauser, 1988 | Undefined/excluded outcomes |
| Murray, 2004 | Undefined/excluded outcomes |
| Oparah, 2006 | Non-experimental study |
| Patel, 2013 | Non-experimental study |
| Pertusa Martinez, 1998 | Non-experimental study |
| Pierce, 1984 | Undefined/excluded outcomes |
| Pippalla, 1994 | Non-experimental study |
| Resnick, 2009 | Non-experimental study |
| Roca-Cusachs, 1991 | Undefined/excluded outcomes |
| Rodriguez, 2011 | Non-experimental study |
| Roumie, 2006 | Non patient-targeted interventions |
| Santschi, 2008 | Non-experimental study |
| Sicras-Mainar, 2013 | Non-experimental study |
| Stewart, 2005 | Undefined/excluded outcomes |
| Stewart, 2008 | Undefined/excluded outcomes |
| van de Steeg-van Gompel, 2010 | Non patient-targeted interventions |
| Wentzlaff, 2011 | Undefined/excluded outcomes |
| Patel, 2015 | Undefined/excluded outcomes |
| van Bruggen, 2009 | Non-experimental study |
| Aslani, 2011 | Undefined/excluded outcomes |
| Flemming, 2013 | Undefined/excluded outcomes |
| Bangsberg, 2009 | Non-experimental study |
| Hornung, 1996 | Undefined/excluded outcomes |
| Pitschel-Walz, 2006 | Excluded medication |
| Williams-a, 2012 | Undefined/excluded outcomes |
| Kender, 2010 | Non-experimental study |
| Martino, 2006 | Undefined/excluded outcomes |
| Macalino, 2007 | Undefined/excluded outcomes |
| Crowley, 2013 | Undefined/excluded outcomes |
| Greer, 2011 | Non-experimental study |
| Margolius, 2012 | Undefined/excluded outcomes |
| Duncan, 2013 | Included pediatrics |
| Otsuki, 2009 | Included pediatrics |
| Hederos, 2005 | Included pediatrics |
| Bender, 2014 | Included pediatrics |
| Liu, 2007 | Undefined/excluded outcomes |
| Ostojic, 2005 | Undefined/excluded outcomes |
| Ryan, 2012 | Undefined/excluded outcomes |
| Chisholm-Burns, 2013 | Undefined/excluded outcomes |
| Fennell, 1994 | Included pediatrics |
| Babigumira, 2011 | Non-experimental study |
| Jaffar, 2009 | Undefined/excluded outcomes |
| Sherrard, 2015 | Undefined/excluded outcomes |
| Belzer, 2014 | Included pediatrics |
| Duncan, 2012 | Out of scope |
| Garcia, 2005 | Undefined/excluded outcomes |
| Naar-King, 2013 | Included pediatrics |
| Orrell, 2015 | Included pediatrics |
| Wamalwa, 2009 | Included pediatrics |
| Yu, 2012 | Non-experimental study |
| Heisig, 2015 | Non-experimental study |
| Graetz, 2017 | Non-experimental study |
| Ell, 2009 | Undefined/excluded outcomes |
| Giallauria, 2009 | Undefined/excluded outcomes |
| Giannuzzi, 2008 | Undefined/excluded outcomes |
| Jorstad, 2013 | Undefined/excluded outcomes |
| Lapointe, 2006 | Undefined/excluded outcomes |
| Miller, 1988 | Undefined/excluded outcomes |
| Redfern, 2008 | Undefined/excluded outcomes |
| Yorio, 2008 | Undefined/excluded outcomes |
| Charrois, 2006 | Undefined/excluded outcomes |
| Cordina, 2001 | Included pediatrics |
| Xaubet Olivera, 2016 | Undefined/excluded outcomes |
| Aluisio, 2011 | Out of scope |
| Kalembo, 2013 | Undefined/excluded outcomes |
| Turan, 2015 | Undefined/excluded outcomes |
| Stinson, 2013 | Undefined/excluded outcomes |
| Kim, 2015 | Undefined/excluded outcomes |
| Dillabaugh, 2012 | Undefined/excluded outcomes |
| Kirsten, 2011 | Undefined/excluded outcomes |
| Mepham, 2011 | Non-experimental study |
| ENHAT-CS, 2014 | Non-experimental study |
| Weiss, 2013 | Undefined/excluded outcomes |
| Okonji, 2012 | Non-experimental study |
| Herlihy, 2015 | Undefined/excluded outcomes |
| Richter, 2014 | Undefined/excluded outcomes |
| Le Roux, 2013 | Undefined/excluded outcomes |
| Kieffer, 2011 | Undefined/excluded outcomes |
| Matthews, 2016 | Out of scope |
| Yotebieng, 2016 | Out of scope |
| Finocchario-Kessler, 2014 | Undefined/excluded outcomes |
| Nyamathi, 2012 | Undefined/excluded outcomes |

- 1. **Studies included in the network meta-analysis**

**0-3 months**

| **Study ID** | **Title** | **Study size** | **Interventions** |
| --- | --- | --- | --- |
| Berg, 2011 | Use of peers to improve adherence to antiretroviral therapy: a global network meta-analysis | 77 | Technical 1st, Standard care 1st |
| Bessa, 2016 | Efficacy of interventions for adherence to the immunosuppressive therapy in kidney transplant recipients: a meta-analysis and systematic review | 124 | Educational 1st, Standard care 1st |
| Bogner, 2008 | Integration of Depression and Hypertension Treatment: A Pilot, Randomized Controlled Trial | 64 | Educational + Attitudinal 1st, Standard care 1st |
| Boissel, 1996 | Comparison between a bid and a tid regimen: improved compliance with no improved antihypertensive effect | 7274 | Technical 1st, Standard care 1st |
| Capoccia, 2004 | Randomized trial of pharmacist interventions to improve depression care and outcomes in primary care | 74 | Educational + Technical 1st, Standard care 1st |
| Chatkin, 2006 | Impact of a Low-Cost and Simple Intervention in Enhancing Treatment Adherence in a Brazilian Asthma Sample | 271 | Educational 1st, Standard care 1st |
| Cheung, 1988 | The paradox of using a 7 day antibacterial course to treat urinary tract infections in the community | 77 | Technical 1st, Standard care 1st |
| Claborn, 2014 | Pilot study examining the efficacy of an electronic intervention to promote HIV medication adherence | 97 | Educational + Attitudinal + Technical 1st, Standard care 1st |
| Cochran, 1984 | Preventing Medical Noncompliance in the Outpatient Treatment of Bipolar Affective Disorders | 28 | Attitudinal 1st , Standard care 1st |
| Cole, 1971 | Drug consultation: its significance to the discharged hospital patient and its relevance as a role for the pharmacist | 50 | Educational 1st, Standard care 1st |
| Collier, 2005 | A Randomized Study of Serial Telephone Call Support to Increase Adherence and Thereby Improve Virologic Outcome in Persons Initiating Antiretroviral Therapy | 171 | Educational + Attitudinal 1st, Standard care 1st |
| Cossette, 2012 | Randomized Controlled Trial of Tailored Nursing Interventions to Improve Cardiac Rehabilitation Enrollment | 243 | Educational + Attitudinal 1st, Standard care 1st |
| Crockett, 2006 | Patient outcomes following an intervention involving community pharmacists in the management of depression | 106 | Educational 1st, Standard care 1st |
| Crome, 1982 | Assessment of a new calendar pack-the C-PAK | 78 | Technical 1st, Standard care 1st |
| Da Costa, 2012 | Results of a randomized controlled trial to assess the effects of a mobile SMS-based intervention on treatment adherence in HIV/AIDS-infected Brazilian women and impressions and satisfaction with respect to incoming messages | 21 | Technical 1st, Standard care 1st |
| Detry, 1995 | Patient compliance and therapeutic coverage: comparison of amlodipine and slow release nifedipine in the treatment of hypertension | 640 | Technical 1st, Standard care 1st |
| Dilorio, 2009 | A telephone-based self-management program for people with epilepsy | 22 | Educational + Attitudinal 1st, Educational 1st |
| Eker, 2012 | Effectiveness of six-week psychoeducation program on adherence of patients with bipolar affective disorder | 71 | Educational + Attitudinal 1st, Standard care 1st |
| Elixhauser, 1990 | The Effects of 'Monitoring and Feedback on Compliance | 90 | Technical 1st, Standard care 1st |
| Elkjaer, 2010 | E-health empowers patients with ulcerative colitis: a randomised controlled trial of the web-guided ‘Constant-care’ approach | 333 | Educational 1st, Standard care 1st |
| Eron, 2000 | Efficacy, safety, and adherence with a twice-daily combination lamivudine/zidovudine tablet formulation, plus a protease inhibitor, in HIV infection | 223 | Technical 1st, Standard care 1st |
| Eshelman, 1976 | Effect of packging on patient compliance with na antihypertensive medication | 100 | Technical 1st, Standard care 1st |
| Faulkner, 2000 | Impact of Pharmacy Counseling on Compliance and Effectiveness of Combination Lipid-Lowering Therapy in Patients Undergoing Coronary Artery Revascularization: A Randomized, Controlled Trial | 30 | Educational 1st, Standard care 1st |
| Fisher, 2011 | Computer-Based Intervention in HIV Clinical Care Setting Improves Antiretroviral Adherence: The LifeWindows Project | 328 | Attitudinal 1st , Standard care 1st |
| Fulmer, 1999 | An Intervention Study to Enhance Medication Compliance in Community-Dwelling Elderly Individuals | 42 | Technical 1st, Standard care 1st |
| Fyllingen, 1991 | Phenoxymethylpenicillin Two or Three Times Daily in Bacterial Upper Respiratory Tract Infections: A Blinded, Randomized and Controlled Clinical Study | 131 | Technical 1st, Standard care 1st |
| Gabriel, 1977 | Improved Patient Compliance through Use of a Daily Drug Reminder Chart | 79 | Technical 1st, Standard care 1st |
| Garcia, 2015 | Behavioral measures to reduce non‑adherence in renal transplant recipients: a prospective randomized controlled trial | 111 | Educational 1st, Standard care 1st |
| García-Cardenas, 2013 | Effect of a pharmacist intervention on asthma control. A cluster randomised trial | 336 | Educational + Attitudinal 1st, Standard care 1st |
| Girvin, 1999 | A comparison of enalapril 20 mg once daily versus 10 mg twice daily in terms of blood pressure lowering and patient compliance Briegeen Girvin, | 27 | Technical 1st, Standard care 1st |
| Golin, 2006 | A 2-Arm, Randomized, Controlled Trial of a Motivational Interviewing–Based Intervention to Improve Adherence to Antiretroviral Therapy (ART) Among Patients Failing or Initiating | 117 | Educational + Attitudinal 1st, Educational 1st |
| Gonzalez-Fernandez, 1990 | Usefulness of a Systemic Hypertension In-Hospital Educational Program | 47 | Educational 1st, Standard care 1st |
| Goswami, 2013 | Impact of an integrated intervention program on atorvastatin adherence: a randomized controlled trial | 208 | Educational 1st, Standard care 1st |
| Henry, 1999 | Enhancing Compliance Not a Prerequisite for Effective Eradication of Helicobacter pylori: The HelP Study | 117 | Educational + Technical 1st, Standard care 1st |
| Hilleman, 1993 | Conversion from Sustained-Release to Immediate-Release Calcium Entry Blockers: Outcome in Patients with Mild-to-Moderate Hypertension | 82 | Technical 1st, Standard care 1st |
| Ho, 2008 | Effect of audible and visual reminders on adherence in glaucoma patients using a commercially available dosing aid | 42 | Technical 1st, Standard care 1st |
| Holzemer, 2006 | Testing a Nurse-Tailored HIV Medication Adherence Intervention | 180 | Educational + Attitudinal 1st, Standard care 1st |
| Horvath, 2013 | Feasibility, Acceptability and Preliminary Efficacy of an Online Peer-to-Peer Social Support ART Adherence Intervention | 123 | Educational + Attitudinal + Technical 1st, Standard care 1st |
| Ingersoll, 2011 | A Pilot Randomized Clinical Trial of Two Medication Adherence and Drug Use Interventions For HIV+ Crack Cocaine Users* | 42 | Attitudinal 1st , Educational 1st |
| Johnson, 2011 | Improving Coping Skills for Self-management of Treatment Side Effects Can Reduce Antiretroviral Medication Nonadherence among People Living with HIV | 249 | Educational + Attitudinal 1st, Standard care 1st |
| Khonsari, 2015 | Effect of a reminder system using an automated short message service on medication adherence following acute coronary syndrome | 62 | Technical 1st, Standard care 1st |
| Kim, 2013 | Effects of a web-based stroke education program on recurrence prevention behaviors among stroke patients: a pilot study | 36 | Educational 1st, Standard care 1st |
| Klang, 2015 | Community pharmacists' support improves antidepressant adherence in the community | 12919 | Technical 1st, Standard care 1st |
| Koenig, 2008 | Randomized Controlled Trial of an Intervention to Prevent Adherence Failure Among HIV-Infected Patients Initiating Antiretroviral Therapy | 226 | Educational + Attitudinal + Technical 1st, Standard care 1st |
| Kotowycz, 2010 | Safety and feasibility of early hospital discharge in ST-segment elevation myocardial infarction | 54 | Educational 1st, Standard care 1st |
| Kronish, 2012 | The Effect of Enhanced Depression Care on Adherence to Risk Reducing Behaviors after Acute Coronary Syndromes: Findings from the COPES Trial | 157 | Attitudinal 1st , Standard care 1st |
| Kubota, 2006 | Short-term Safety and Tolerability of a Once-Daily Fixed-Dose Abacavir-Lamivudine Combination versus Twice-Daily Dosing of Abacavir and Lamivudine as Separate Components: Findings from the ALOHA Study | 680 | Technical 1st, Standard care 1st |
| Lee, 1999 | A Randomized Controlled Trial of an Enhanced Patient Compliance Program for Helicobacter pylori Therapy | 125 | Educational + Technical 1st, Standard care 1st |
| Lopez Cabezas, 2006 | Randomized clinical trial of a postdischarge pharmaceutical care program vs. regular follow-up in patients with heart failure | 94 | Educational 1st, Standard care 1st |
| Lv, 2012 | A mobile phone short message service improves perceived control of asthma: a randomized controlled trial | 71 | Educational 1st, Educational + Technical 1st, Standard care 1st |
| MacIntosh, 2007 | A comparison of patient adherence and preference of packaging method for oral anticancer agents using conventional pill bottles versus daily pill boxes | 42 | Technical 1st, Standard care 1st |
| Maduka, 2013 | Adherence counseling and reminder text messages improve uptake of antiretroviral therapy in a tertiary hospital in Nigeria | 104 | Educational + Attitudinal + Technical 1st, Standard care 1st |
| Marquez Contreas, 2004 | Effectiveness of an intervention to provide information to patients with hypertension as short text messages and reminders sent to their mobile phone (HTA-Alert) | 67 | Educational + Technical 1st, Standard care 1st |
| Marquez-Contreras, 2004 | Effectiveness of an Intervention to Provide Information to Patients With Hypertension as Short Text Messages of Reminders Sent to Their Mobile Phone (HTA-Alert) | 67 | Educational 1st, Standard care 1st |
| Martinot, 2001 | A Comparative Study of Clarithromycin Modified Release and Amoxicillin/ Clavulanic Acid in the Treatment of Acute Exacerbation of Chronic Bronchitis | 250 | Technical 1st, Standard care 1st |
| Matsumura, 2012 | Does a Combination Pill of Antihypertensive Drugs Improve Medication Adherence in Japanese? | 207 | Technical 1st, Standard care 1st |
| Mbuagbaw, 2012 | Mobile phone text messages for improving adherence to antiretroviral therapy (ART): an individual patient data meta-analysis of randomised trials | 200 | Technical 1st, Standard care 1st |
| Mooney, 2005 | Interventions to increase use of nicotine gum: A randomized, controlled, single-blind trial | 97 | Technical 1st, Rewards + Technical 1st, Standard care 1st |
| Mooney, 2007 | Adding MEMS feedback to behavioral smoking cessation therapy increases compliance with bupropion: A replication and extension study | 55 | Attitudinal + Technical 1st, Attitudinal 1st |
| Moss, 2010 | Impact of a patient-support program on mesalamine adherence in patients with ulcerative colitis — A prospective study | 62 | Educational + Attitudinal 1st, Standard care 1st |
| Najafi, 2016 | The Nurse-Led Telephone Follow-Up on Medication and Dietary Adherence among Patients after Myocardial Infarction: A Randomized Controlled Clinical Trial | 100 | Educational 1st, Standard care 1st |
| Nance, 2017 | Short-term effectiveness of a community health worker intervention for HIV-infected pregnant women in Tanzania to improve treatment adherence and retention in care: A cluster-randomized trial | 1830 | Educational 1st, Standard care 1st |
| Nazareth, 2001 | A pharmacy discharge plan for hospitalized elderly patients – a randomized controlled trial | 362 | Educational + Attitudinal 1st, Standard care 1st |
| Nielson, 2010 | Patient education in groups increases knowledge of osteoporosis and adherence to treatment: A two-year randomized controlled trial | 300 | Educational 1st, Standard care 1st |
| Ollivier, 2009 | Use of short message service (SMS) to improve malaria chemoprophylaxis compliance after returning from a malaria endemic area | 335 | Technical 1st, Standard care 1st |
| Park, 1992 | Medication Adherence Behaviors in Older Adults: Effects of External Cognitive Supports | 61 | Technical 1st, Standard care 1st |
| Parsons, 2007 | Motivational Interviewing and Cognitive-Behavioral Intervention to Improve HIV Medication Adherence Among Hazardous Drinkers: A Randomized Controlled Trial | 115 | Attitudinal 1st , Educational 1st |
| Pearson, 2007 | Randomized Control Trial of Peer-Delivered, Modified Directly Observed Therapy for HAART in Mozambique | 350 | Educational + Technical 1st, Standard care 1st |
| Polack, 2008 | Evaluation of different methods of providing medication-related education to patients following | 14 | Educational 1st, Standard care 1st |
| Pullar, 1988 | Use of a pharmacologic indicator to compare compliance with tablets prescribed to be taken once, twice, or three times daily | 179 | Technical 1st, Standard care 1st |
| Purcell, 2007 | Results From a Randomized Controlled Trial of a Peer-Mentoring Intervention to Reduce HIV Transmission and Increase Access to Care and Adherence | 430 | Attitudinal 1st , Educational 1st |
| Ramirez-Garcia & Cote, 2012 | An Individualized Intervention to Foster Optimal Antiretroviral Treatment-Taking Behavior Among Persons Living With HIV: A Pilot Randomized Controlled Trial | 44 | Educational + Attitudinal 1st, Standard care 1st |
| Raynor, 1993 | Effects of computer generated reminder charts on patients' compliance with drug regimens | 191 | Educational 1st, Educational + Technical 1st |
| Rich, 1995 | A multidisciplinary intervention to prevent the readmission of elderly Patients with congestive heart failure | 282 | Educational 1st, Standard care 1st |
| Rich, 1996 | Effect of a Multidisciplinary Intervention on Medication Compliance in Elderly Patients with Congestive Heart Failure | 156 | Educational 1st, Standard care 1st |
| Rigsby, 2000 | Cue-dose Training with Monetary Reinforcement Pilot Study of an Antiretroviral Adherence Intervention | 55 | Technical 1st, Attitudinal + Technical + Rewards 1st, Standard care 1st |
| Rinfret, 2013 | Telephone contact to improve adherence to dual antiplatelet therapy after drug-eluting stent implantation | 300 | Educational 1st, Standard care 1st |
| Roden, 1985 | Evaluation of two techniques to improve drug Compliance in the elderly | 84 | Technical 1st, Educational 1st, Standard care 1st |
| Rozenfeld, 1999 | Assessing the Impact of Medication Consultations with a Medication Event Monitoring System | 33 | Educational 1st, Standard care 1st |
| Rubio-Valera, 2013 | Evaluation of a pharmacist intervention on patients initiating pharmacological treatment for depression: A randomized controlled superiority trial | 179 | Educational 1st, Standard care 1st |
| Schaffer and Tian, 2004 | Promoting Adherence Effects of Theory-Based Asthma Education | 70 | Educational 1st, Standard care 1st |
| Schmitz, 2005 | Medication Compliance During a Smoking Cessation Clinical Trial: A Brief Intervention Using MEMS Feedback | 97 | Technical 1st, Standard care 1st |
| Silveira, 2014 | Randomized Controlled Trial to Evaluate the Impact of Pharmaceutical Care on Therapeutic Success in HIV-Infected Patients in Southern Brazil | 332 | Educational 1st, Standard care 1st |
| Smith, 2003 | A medication self-management program to improve adherence to HIV therapy regimens | 43 | Educational + Attitudinal 1st, Standard care 1st |
| Strandbygaard, 2010 | A daily SMS reminder increases adherence to asthma treatment: A three-month follow-up study | 22 | Technical 1st, Standard care 1st |
| Sweeney, 1989 | The impact of the clinical pharmacist on compliance in a geriatric population | 103 | Educational 1st, Standard care 1st |
| Tuldra, 2000 | Prospective Randomized Two-Arm Controlled Study To Determine the Efficacy of a Specific Intervention To Improve Long-Term Adherence to Highly Active Antiretroviral Therapy | 116 | Attitudinal 1st , Standard care 1st |
| Volpp, 2008 | A test of financial incentives to improve warfarin adherence | 45 | Rewards 1st, Standard care 1st |
| Wagner, 2006 | Cognitive-behavioral intervention to enhance adherence to antiretroviral therapy: a randomized controlled trial (CCTG 578) | 199 | Attitudinal 1st , Standard care 1st |
| Wagner, 2013 | Pilot Controlled Trial of the Adherence Readiness Program: An Intervention to Assess and Sustain HIV Antiretroviral Adherence Readiness | 60 | Educational + Attitudinal 1st, Standard care 1st |
| Wakefield, 2009 | Outcomes of a home telehealth intervention for patients with heart failure | 89 | Educational 1st, Standard care 1st |
| Wang, 2014 | A Randomized Controlled Trial to Assess Adherence to Allergic Rhinitis Treatment following a Daily Short Message Service (SMS) | 50 | Technical 1st, Standard care 1st |
| Williams, 2006 | Home Visits to Improve Adherence to Highly Active Antiretroviral Therapy: A Randomized Controlled Trial | 171 | Educational + Attitudinal 1st, Standard care 1st |
| Williams, 2012 | A multifactorial intervention to improve blood pressure control in co-existing diabetes and kidney disease: a feasibility randomized controlled trial | 75 | Educational + Attitudinal + Technical 1st, Standard care 1st |
| Wong, 2013 | Effectiveness of a Pharmacist‐Led Drug Counseling on Enhancing Antihypertensive Adherence and Blood Pressure Control: A Randomized Controlled Trial | 231 | Educational + Technical 1st, Standard care 1st |
| Wong, 2017 | A pharmacy management service for adults with asthma: a cluster randomised controlled trial | 157 | Educational 1st, Standard care 1st |
| Wu, 2012 | Effect of a Medication-Taking Behavior Feedback, Theory-Based Intervention on Outcomes in Patients with Heart Failure | 82 | Attitudinal 1st , Educational + Attitudinal + Technical 1st, Standard care 1st |
| Wyatt, 2004 | The Efficacy of an Integrated Risk Reduction Intervention for HIV-PositiveWomen With Child Sexual Abuse Histories | 147 | Attitudinal 1st , Standard care 1st |
| Zillich, 2005 | Hypertension Outcomes Through Blood Pressure Monitoring and Evaluation by Pharmacists (HOME Study) | 125 | Educational 1st, Standard care 1st |

**4-6 months**

| **Study ID** | **Title** | **Study size** | **Interventions** |
| --- | --- | --- | --- |
| Altice, 2007 | Superiority of Directly Administered Antiretroviral Therapy over Self-Administered Therapy among HIV-Infected Drug Users: A Prospective, Randomized, Controlled Trial | 141 | Technical 2nd, Standard care 2nd |
| Ball, 2006 | A Randomized Controlled Trial of Cognitive Therapy for Bipolar Disorder: Focus on Long-Term Change | 52 | Standard care 2nd, Attitudinal 2nd |
| Basso, 2013 | Exploring ART Intake Scenes in a Human Rights-Based Intervention to Improve Adherence: A Randomized Controlled Trial | 108 | Standard care 2nd, Educational + Attitudinal 2nd |
| Berg, 2011 | Directly observed antiretroviral therapy improves adherence and viral load in drug users attending methadone maintenance clinics: a randomized controlled trial* | 77 | Technical 2nd, Standard care 2nd |
| Bessa, 2016 | Prospective Randomized Trial Investigating the Influence of Pharmaceutical Care on the Intra-Individual Variability of Tacrolimus Concentrations Early After Kidney Transplant | 124 | Educational 2nd, Standard care 2nd |
| Beune, 2014 | Culturally Adapted Hypertension Education (CAHE) to Improve Blood Pressure Control and Treatment Adherence in Patients of African Origin with Uncontrolled Hypertension: Cluster-Randomized Trial | 139 | Educational 2nd, Standard care 2nd |
| Blenkinsopp, 2000 | Extended adherence 0 support Ir by 0 community pharmac with hypertension: a controlled trial | 232 | Educational 2nd, Standard care 2nd |
| Bosworth, 2008 | Take Control of Your Blood pressure (TCYB) study: A multifactorial tailored behavioral and educational intervention for achieving blood pressure control | 636 | Standard care 2nd, Educational + Attitudinal + Technical 2nd |
| Bouvy, 2003 | Effect of a Pharmacist-Led Intervention on Diuretic Compliance in Heart Failure Patients: A Randomized Controlled Study | 152 | Educational 2nd, Standard care 2nd |
| Brook, 2005 | A Pharmacy-Based Coaching Program to Improve Adherence to Antidepressant Treatment Among Primary Care Patients | 135 | Educational 2nd, Standard care 2nd |
| Brown, 1997 | Moderate Dose, Three-Drug Therapy With Niacin, Lovastatin, and Colestipol | 29 | Technical 2nd, Standard care 2nd |
| Burrelle, 1987 | Evaluation of an Interdisciplinary Compliance Service for Elderly Hypertensives | 16 | Standard care 2nd, Educational + Technical 2nd |
| Calvert, 2012 | Patient-focused intervention to improve long-term adherence to evidence-based medications: A randomized trial | 143 | Educational 2nd, Educational + Technical 2nd |
| Capoccia, 2004 | Randomized trial of pharmacist interventions to improve depression care and outcomes in primary care | 74 | Standard care 2nd, Educational + Technical 2nd |
| Chaisson, 2001 | A Randomized, Controlled Trial of Interventions to Improve Adherence to Isoniazid Therapy to Prevent Tuberculosis in Injection Drug Users | 300 | Technical 2nd, Educational 2nd, Standard care 2nd |
| Charles, 2007 | An audiovisual reminder function improves adherence with inhaled corticosteroid therapy in asthma | 90 | Technical 2nd, Standard care 2nd |
| Cochran, 1984 | Preventing Medical Noncompliance in the Outpatient Treatment of Bipolar Affective Disorders | 28 | Standard care 2nd, Attitudinal 2nd |
| Costa, 2008 | Transdiciplinary approach to the follow-up of patients after myocardial infarction | 142 | Educational 2nd, Standard care 2nd |
| Da Costa, 2012 | Results of a randomized controlled trial to assess the effects of a mobile SMS-based intervention on treatment adherence in HIV/AIDS-infected Brazilian women and impressions and satisfaction with respect to incoming messages | 21 | Technical 2nd, Standard care 2nd |
| Eron, 2000 | Efficacy, safety, and adherence with a twice-daily combination lamivudine/zidovudine tablet formulation, plus a protease inhibitor, in HIV infection | 223 | Technical 2nd, Standard care 2nd |
| Evans, 2010 | The Collaborative Cardiovascular Risk Reduction in Primary Care (CCARP) Study | 176 | Standard care 2nd, Educational + Technical 2nd |
| Falces, 2008 | [An educative intervention to improve treatment compliance and to prevent readmissions of elderly patients with heart failure] | 103 | Educational 2nd, Standard care 2nd |
| Finley, 2003 | Impact of a Collaborative Care Model on Depression in a Primary Care Setting: A Randomized Controlled Trial | 125 | Standard care 2nd, Educational + Technical 2nd |
| Foster, 2014 | Inhaler reminders improve adherence with controller treatment in primary care patients with asthma | 129 | Technical 2nd, Educational 2nd, Standard care 2nd, Educational + Technical 2nd |
| Gamble, 2011 | A study of a multi-level intervention to improve non-adherence in difficult to control asthma | 18 | Standard care 2nd, Educational + Attitudinal 2nd |
| García-Cardenas, 2013 | "Effect of a pharmacist intervention on asthma control. A cluster randomised trial" | 336 | Standard care 2nd, Educational + Attitudinal 2nd |
| Geiter, 1987 | United States public health service tuberculosis therapy Trial 21: preliminary results of an evaluation of a Combination tablet of isoniazid, rifampin and pyrazinamide | 460 | Technical 2nd, Standard care 2nd |
| Gross, 2009 | Modified Directly Observed Antiretroviral Therapy Compared with Self-Administered Therapy in Treatment-Naïve HIV-1 Infected Patients: A Randomized Trial | 243 | Technical 2nd, Standard care 2nd |
| Gross, 2015 | Partner-Focused Adherence Intervention for Second-line Antiretroviral Therapy: A Multinational Randomized Trial (ACTG A5234) | 257 | Standard care 2nd, Educational + Attitudinal + Technical 2nd |
| Holstad, 2012 | Motivational Groups Support Adherence to Antiretroviral Therapy and use of Risk Reduction Behaviors in HIV Positive Nigerian Women: A Pilot Study | 48 | Educational 2nd, Attitudinal 2nd |
| Holzemer, 2006 | Testing a Nurse-Tailored HIV Medication Adherence Intervention | 180 | Standard care 2nd, Educational + Attitudinal 2nd |
| Ingersoll, 2011 | A Pilot Randomized Clinical Trial of Two Medication Adherence and Drug Use Interventions For HIV+ Crack Cocaine Users* | 42 | Educational 2nd, Attitudinal 2nd |
| Jarab, 2012 | Randomized Controlled Trial of Clinical Pharmacy Management of Patients with Type 2 Diabetes in an Outpatient Diabetes Clinic in Jordan | 171 | Educational 2nd, Standard care 2nd |
| Johnson, 2011 | Improving Coping Skills for Self-management of Treatment Side Effects Can Reduce Antiretroviral Medication Nonadherence among People Living with HIV | 249 | Standard care 2nd, Educational + Attitudinal 2nd |
| Joost, 2014 | Intensified pharmaceutical care is improving immunosuppressive medication adherence in kidney transplant recipients during the first post-transplant year: a quasi-experimental study | 74 | Standard care 2nd, Educational + Technical 2nd |
| Katon, 1996 | A Multifaceted Intervention to Improve Treatment of Depression in Primary Care | 153 | Standard care 2nd, Educational + Attitudinal 2nd |
| Kelly, 1990 | Medication Compliance and Health Education Among Outpatients With Chronic Mental Disorders | 418 | Educational 2nd, Standard care 2nd |
| Klang, 2015 | Community pharmacists' support improves antidepressant adherence in the community | 12919 | Technical 2nd, Standard care 2nd |
| Koenig, 2008 | Randomized Controlled Trial of an Intervention to Prevent Adherence Failure Among HIV-Infected Patients Initiating Antiretroviral Therapy | 226 | Standard care 2nd, Educational + Attitudinal + Technical 2nd |
| Konkle-Parker, 2012 | Pilot testing of an HIV medication adherence intervention in a public clinic in the Deep South | 36 | Standard care 2nd, Attitudinal 2nd |
| Konkle-Parker, 2014 | "Effects of an Intervention Addressing Information, Motivation, and Behavioral Skills on HIV Care Adherence in a Southern Clinic Cohort" | 100 | Standard care 2nd, Educational + Attitudinal + Technical 2nd |
| Kopelowicz, 2003 | Disease Management in Latinos With Schizophrenia: A Family-Assisted, Skills Training Approach | 92 | Standard care 2nd, Educational + Attitudinal 2nd |
| Kronish, 2012 | The Effect of Enhanced Depression Care on Adherence to Risk Reducing Behaviors after Acute Coronary Syndromes: Findings from the COPES Trial | 157 | Standard care 2nd, Attitudinal 2nd |
| Lee, 1996 | Assessing Medication Adherence by Pill Count and Electronic Monitoring in the African American Shdy of Kidney Disease and Hypertension (AASK) Pilot Study | 313 | Technical 2nd, Standard care 2nd |
| Levin, 2006 | A Randomized Trial of Educational Materials, Pillboxes, and Mailings to Improve Adherence with Antiretroviral Therapy in an Inner City HIV Clinic | 49 | Standard care 2nd, Educational + Technical 2nd |
| Levine, 1979 | Health Education for Hypertensive Patients | 325 | Standard care 2nd, Educational + Attitudinal 2nd |
| Lopez Cabezas, 2006 | Randomized clinical trial of a postdischarge pharmaceutical care program vs. regular follow-up in patients with heart failure | 74 | Educational 2nd, Standard care 2nd |
| Magid, 2011 | A Multimodal Blood Pressure Control Intervention in 3 Healthcare Systems | 283 | Educational 2nd, Educational + Technical 2nd |
| Marquez Contreas, 2004 | Effectiveness of an intervention to provide information to patients with hypertension as short text messages and reminders sent to their mobile phone (HTA-Alert) | 67 | Standard care 2nd, Educational + Technical 2nd |
| Marquez-Contreras, 2004 | Effectiveness of an Intervention to Provide Information to Patients With Hypertension as Short Text Messages of Reminders Sent to Their Mobile Phone (HTA-Alert) | 67 | Educational 2nd, Standard care 2nd |
| Marquez-Contreras, 2005 | Efficacy of telephone and mail intervention in patient compliance with antihypertensive drugs in hypertension. ETECUM-HTA study | 538 | Technical 2nd, Educational 2nd, Standard care 2nd |
| Marquez-Contreras, 2007 | Therapy compliance in cases of hyperlipaemia, as measured through electronic monitors. Is a reminder calendar to avoid forgetfulness effective? | 220 | Technical 2nd, Standard care 2nd |
| Matsumura, 2012 | Does a Combination Pill of Antihypertensive Drugs Improve Medication Adherence in Japanese? | 207 | Technical 2nd, Standard care 2nd |
| Mbuagbaw, 2012 | Mobile phone text messages for improving adherence to antiretroviral therapy (ART): an individual patient data meta-analysis of randomised trials | 200 | Technical 2nd, Standard care 2nd |
| McKenney, 1973 | The Effect of Clinical Pharmacy Services on Patients with Essential Hypertension | 49 | Educational 2nd, Standard care 2nd |
| McKinstry, 2013 | Telemonitoring based service redesign for the management of uncontrolled hypertension: multicentre randomised controlled trial | 401 | Technical 2nd, Standard care 2nd |
| Mckenney, 1978 | Effect of Pharmacist Drug Monitoring and Patient Education on Hypertensive Patients | 135 | Educational 2nd, Standard care 2nd |
| Mehos, 2000 | Effect of Pharmacist Intervention and Initiation of Home Blood Pressure Monitoring in Patients with Uncontrolled Hypertension | 36 | Educational 2nd, Educational + Technical 2nd |
| Mehuys, 2008 | Effectiveness of pharmacist intervention for asthma control improvement | 150 | Educational 2nd, Standard care 2nd |
| Mols, 2015 | Visualization of Coronary Artery Calcification: Influence on Risk Modification | 189 | Educational 2nd, Standard care 2nd |
| Montori, 2011 | Use of a Decision Aid to Improve Treatment Decisions in Osteoporosis: The Osteoporosis Choice Randomized Trial | 100 | Educational 2nd, Standard care 2nd |
| Moss, 2010 | Impact of a patient-support program on mesalamine adherence in patients with ulcerative colitis — A prospective study | 62 | Standard care 2nd, Educational + Attitudinal 2nd |
| Nachega, 2010 | Randomized Controlled Trial of Trained Patient-Nominated Treatment Supporters Providing Partial Directly Observed Antiretroviral Therapy | 274 | Standard care 2nd, Educational + Technical 2nd |
| Nazareth, 2001 | A pharmacy discharge plan for hospitalized elderly patients – a randomized controlled trial | 362 | Standard care 2nd, Educational + Attitudinal 2nd |
| Nunes, 2006 | Behavioral Therapy to Augment Oral Naltrexone for Opioid Dependence: A Ceiling on Effectiveness? | 69 | Educational 2nd, Attitudinal + Rewards 2nd |
| Ostbring, 2014 | Medication beliefs and self-reported adherence- results of a pharmacist's consultation: a pilot study | 21 | Standard care 2nd, Attitudinal 2nd |
| Parsons, 2007 | Motivational Interviewing and Cognitive-Behavioral Intervention to Improve HIV Medication Adherence Among Hazardous Drinkers: A Randomized Controlled Trial | 115 | Educational 2nd, Attitudinal 2nd |
| Pearson, 2007 | Randomized Control Trial of Peer-Delivered, Modified Directly Observed Therapy for HAART in Mozambique | 350 | Standard care 2nd, Educational + Technical 2nd |
| Petrie, 2012 | A text message programme designed to modify patients’ illness and treatment beliefs improves self-reported adherence to asthma preventer medication | 103 | Educational 2nd, Standard care 2nd |
| Pladevall, 2010 | A multi-center cluster-randomized trial of a multifactorial Intervention to improve antihypertensive Medication adherence and blood pressure control Among patients at high cardiovascular risk (the Com99 study)* | 875 | Standard care 2nd, Educational + Attitudinal 2nd |
| Pradier, 2003 | Efficacy of an Educational and Counseling Intervention on Adherence to Highly Active Antiretroviral Therapy: French Prospective | 202 | Standard care 2nd, Educational + Attitudinal 2nd |
| Purcell, 2007 | Results From a Randomized Controlled Trial of a Peer-Mentoring Intervention to Reduce HIV Transmission and Increase Access to Care and Adherence | 405 | Educational 2nd, Attitudinal 2nd |
| Pyne, 2011 | Effectiveness of Collaborative Care for Depression in Human Immunodeficiency Virus Clinics | 194 | Standard care 2nd, Educational + Technical 2nd |
| Ramirez-Garcia & Cote, 2012 | An Individualized Intervention to Foster Optimal Antiretroviral Treatment-Taking Behavior Among Persons Living With HIV: A Pilot Randomized Controlled Trial | 44 | Standard care 2nd, Educational + Attitudinal 2nd |
| Rawlings, 2003 | Impact of an Educational Program on Efficacy and Adherence With a Twice-Daily Lamivudine/Zidovudine/Abacavir Regimen in Underrepresented HIV-Infected Patients | 195 | Educational 2nd, Standard care 2nd |
| Rehder, 1980 | Improving medication compliance by counseling and special prescription container | 100 | Technical 2nd, Educational 2nd, Standard care 2nd, Educational + Technical 2nd |
| Rinfret, 2013 | Telephone contact to improve adherence to dual antiplatelet therapy after drug-eluting stent implantation | 300 | Educational 2nd, Standard care 2nd |
| Rubio-Valera, 2013 | Evaluation of a pharmacist intervention on patients initiating pharmacological treatment for depression: A randomized controlled superiority trial | 179 | Educational 2nd, Standard care 2nd |
| Sackett, 1975 | Randomised clinical trial of strategies for improving medication compliance in primary hypertension | 230 | Educational 2nd, Standard care 2nd |
| Safren, 2003 | Use of an on-line pager system to increase adherence to antiretroviral medications | 60 | Technical 2nd, Standard care 2nd |
| Samet, 2005 | A randomized controlled trial to enhance antiretroviral therapy adherence in patients with a history of alcohol problems | 138 | Standard care 2nd, Educational + Attitudinal + Technical 2nd |
| Saunders, 1991 | A Randomized Controlled Trial of Compliance Improving Strategies in Soweto Hypertensives | 115 | Educational 2nd, Educational + Technical 2nd |
| Schaffer and Tian, 2004 | Promoting Adherence Effects of Theory-Based Asthma Education | 70 | Educational 2nd, Standard care 2nd |
| Selke, 2010 | Task-Shifting of Antiretroviral Delivery From Health Care Workers to Persons Living With HIV/AIDS: Clinical Outcomes of a Community-Based Program in Kenya | 208 | Educational 2nd, Standard care 2nd |
| Sherrard, 2009 | Using Technology to Create a Medication Safety Net for Cardiac Surgery Patients: A Nurse-Led Randomized Control Trial | 331 | Technical 2nd, Standard care 2nd |
| Simon, 2011 | Randomized Trial of Depression Follow-Up Care by Online Messaging | 208 | Technical 2nd, Standard care 2nd |
| Simoni, 2013 | A Preliminary RCT of CBT-AD for Adherence and Depression among HIV-Positive Latinos on the U.S. –Mexico Border: The Nuevo Día Study | 40 | Standard care 2nd, Educational + Attitudinal + Technical 2nd |
| Sookaneknun, 2004 | Pharmacist Involvement in Primary Care Improves Hypertensive Patient Clinical Outcomes | 235 | Standard care 2nd, Educational + Technical 2nd |
| Stacy, 2009 | Incorporating Tailored Interactive Patient Solutions Using Interactive Voice Response Technology to Improve Statin Adherence: Results of a Randomized Clinical Trial in a Managed Care Setting | 497 | Standard care 2nd, Educational + Technical 2nd |
| Stewart, 2014 | A multifaceted pharmacist intervention to improve antihypertensive adherence: a cluster-randomized, controlled trial (HAPPy trial) | 354 | Standard care 2nd, Educational + Attitudinal + Technical 2nd |
| Taiwo, 2010 | Assessing the Viorologic and Adherence Benefits of Patient-Selected HIV Treatment Partners in a Resource-limited Setting | 499 | Technical 2nd, Standard care 2nd |
| Tsuyuki, 2004 | A Multicenter Disease Management Program for Hospitalized Patients With Heart Failure | 276 | Standard care 2nd, Educational + Technical 2nd |
| Tuldra, 2000 | Prospective Randomized Two-Arm Controlled Study To Determine the Efficacy of a Specific Intervention To Improve Long-Term Adherence to Highly Active Antiretroviral Therapy | 116 | Standard care 2nd, Attitudinal 2nd |
| Wagner, 2006 | Cognitive-behavioral intervention to enhance adherence to antiretroviral therapy: a randomized controlled trial (CCTG 578) | 199 | Standard care 2nd, Attitudinal 2nd |
| Wagner, 2013 | Pilot Controlled Trial of the Adherence Readiness Program: An Intervention to Assess and Sustain HIV Antiretroviral Adherence Readiness | 60 | Standard care 2nd, Educational + Attitudinal 2nd |
| Wakefield, 2009 | Outcomes of a home telehealth intervention for patients with heart failure | 89 | Educational 2nd, Standard care 2nd |
| Wald, 2014 | Randomised Trial of Text Messaging on Adherence to Cardiovascular Preventive treatment (INTERACT Trial) | 301 | Technical 2nd, Standard care 2nd |
| Williams, 2006 | Home Visits to Improve Adherence to Highly Active Antiretroviral Therapy: A Randomized Controlled Trial | 171 | Standard care 2nd, Educational + Attitudinal 2nd |
| Williams, 2014 | Efficacy of an Evidence-Based ARV Adherence Intervention in China | 110 | Standard care 2nd, Educational + Attitudinal 2nd |
| Wohl, 2006 | A Randomized Trial of Directly Administered Antiretroviral Therapy and Adherence Case Management Intervention | 96 | Technical 2nd, Educational 2nd, Standard care 2nd |
| Wong, 2013 | Effectiveness of a Pharmacist‐Led Drug Counseling on Enhancing Antihypertensive Adherence and Blood Pressure Control: A Randomized Controlled Trial | 231 | Standard care 2nd, Educational + Technical 2nd |
| Wong, 2017 | A pharmacy management service for adults with asthma: a cluster randomised controlled trial | 157 | Educational 2nd, Standard care 2nd |
| Zaretsky, 2008 | Is Cognitive-Behavioural Therapy More Effective Than Psychoeducation in Bipolar Disorder? | 79 | Educational 2nd, Educational + Attitudinal 2nd |
| van Gent, 1991 | Psychoeducation of partners of bipolar-manic patients | 39 | Educational 2nd, Standard care 2nd |

**7-9 months**

| **Study ID** | **Title** | **Study size** | **Interventions** |
| --- | --- | --- | --- |
| Asplund, 1984 | Patients compliance in hypertension-the importance of number of tablets | 160 | Technical 3rd, Standard care 3rd |
| Brown, 1997 | Moderate Dose, Three-Drug Therapy With Niacin, Lovastatin, and Colestipol to Reduce Low-Density Lipoprotein Cholesterol ò100 mg/dl in Patients With Hyperlipidemia and Coronary Artery Disease | 29 | Technical 3rd, Standard care 3rd |
| Capoccia, 2004 | Randomized trial of pharmacist interventions to improve depression care and outcomes in primary care | 74 | Educational + Technical 3rd, Standard care 3rd |
| Castellano, 2014 | A Polypill Strategy to Improve Adherence Results From the FOCUS Project | 695 | Technical 3rd, Standard care 3rd |
| Dusing, 2009 | Impact of supportive measures on drug adherence in patients with essential hypertension treated with valsartan: the randomized, open-label, parallel group study VALIDATE | 202 | Educational + Technical 3rd, Standard care 3rd |
| Johnson, 2011 | Improving Coping Skills for Self-management of Treatment Side Effects Can Reduce Antiretroviral Medication Nonadherence among People Living with HIV | 249 | Educational + Attitudinal 3rd, Standard care 3rd |
| Katon, 1996 | A Multifaceted Intervention to Improve Treatment of Depression in Primary Care | 153 | Educational + Attitudinal 3rd, Standard care 3rd |
| Kronish, 2012 | The Effect of Enhanced Depression Care on Adherence to Risk Reducing Behaviors after Acute Coronary Syndromes: Findings from the COPES Trial | 157 | Attitudinal 3rd, Standard care 3rd |
| Margolin, 2003 | A Randomized Clinical Trial of a Manual-Guided Risk Reduction Intervention for HIV-Positive Injection Drug Users | 69 | Educational + Attitudinal 3rd, Educational 3rd |
| Peterson, 1984 | A Randomised Trial of Strategies to Improve Patient Compliance with Anticonvulsant Therapy | 53 | Educational + Technical 3rd, Standard care 3rd |
| Powell, 1995 | Failure of educational videotapes to improve medication compliance in a health maintenance organization | 4246 | Educational 3rd, Standard care 3rd |
| Rathburn, 2005 | Impact of an Adherence Clinic on Behavioral Outcomes and Virologic Response in the Treatment of HIV Infection: A Prospective, Randomized, Controlled Pilot Study | 33 | Educational + Technical 3rd, Standard care 3rd |
| Simoni, 2009 | An RCT of Peer Support and Pager Messaging to Promote Antiretroviral Therapy Adherence and Clinical Outcomes among Adults Initiating or Modifying Therapy in Seattle, WA, USA | 224 | Educational + Attitudinal + Technical 3rd, Educational + Attitudinal 3rd, Educational + Technical 3rd, Standard care 3rd |
| Simoni, 2013 | "A Preliminary RCT of CBT-AD for Adherence and Depression among HIV-Positive Latinos on the U.S. –Mexico Border: The Nuevo Día Study" | 40 | Educational + Attitudinal + Technical 3rd, Standard care 3rd |
| Smith, 2008 | A Randomized Trial of Direct-to-Patient Communication to Enhance Adherence to B-Blocker Therapy Following Myocardial Infarction | 888 | Educational 3rd, Standard care 3rd |
| Velligan, 2008 | The Use of Individually Tailored Environmental Supports to Improve Medication Adherence and Outcomes in Schizophrenia | 61 | Attitudinal 3rd, Standard care 3rd |
| Williams, 2006 | Home Visits to Improve Adherence to Highly Active Antiretroviral Therapy: A Randomized Controlled Trial | 171 | Educational + Attitudinal 3rd, Standard care 3rd |
| Wu, 2012 | Effect of a Medication-Taking Behavior Feedback, Theory-Based Intervention on Outcomes in Patients with Heart Failure | 82 | Educational + Attitudinal + Technical 3rd, Attitudinal 3rd, Standard care 3rd |

**≥10 months**

| **Study ID** | **Title** | **Study size** | **Interventions** |
| --- | --- | --- | --- |
| Antonicelli, 2010 | Impact of Home Patient Telemonitoring on Use of ß-Blockers in Congestive Heart Failure | 57 | Educational + Technical 4th, Standard care 4th |
| Ball, 2006 | A Randomized Controlled Trial of Cognitive Therapy for Bipolar Disorder: Focus on Long-Term Change | 52 | Attitudinal 4th, Standard care 4th |
| Boyle, 2008 | Randomization to Once-Daily Stavudine Extended Release/Lamivudine/Efavirenz Versus a More Frequent Regimen Improves Adherence While Maintaining Viral Suppression | 300 | Technical 4th, Standard care 4th |
| Brankin, 2006 | The impact of dosing frequency on compliance and persistence with bisphosphonates among postmenopausal women in the UK: evidence from three databases | 15330 | Technical 4th, Standard care 4th |
| Broekhuizen, 2012 | Can Multiple Lifestyle Behaviours Be Improved in People with Familial Hypercholesterolemia? Results of a Parallel Randomised Controlled Trial | 224 | Educational + Attitudinal 4th, Standard care 4th |
| Capoccia, 2004 | Randomized trial of pharmacist interventions to improve depression care and outcomes in primary care | 74 | Educational + Technical 4th, Standard care 4th |
| Chang, 2010 | Effect of Peer Health Workers on AIDS Care in Rakai, Uganda: A Cluster-Randomized Trial | 1203 | Educational + Technical 4th, Standard care 4th |
| Choudhry, 2011 | Full Coverage for Preventive Medications after Myocardial Infarction | 5855 | Rewards 4th, Standard care 4th |
| Clowes, 2004 | The Impact of Monitoring on Adherence and Persistence with Antiresorptive Treatment for Postmenopausal Osteoporosis: A Randomized Controlled Trial | 48 | Technical 4th, Standard care 4th |
| Collier, 2005 | A Randomized Study of Serial Telephone Call Support to Increase Adherence and Thereby Improve Virologic Outcome in Persons Initiating Antiretroviral Therapy | 101 | Educational + Attitudinal 4th, Standard care 4th |
| Cramer, 2005 | Compliance and persistence with bisphosphonate dosing regimens among women with postmenopausal osteoporosis | 2741 | Technical 4th, Standard care 4th |
| Cramer, 2006 | The Effect of Dosing Frequency on Compliance and Persistence with Bisphosphonate Therapy in Postmenopausal Women: A Comparison of Studies in the United States, the United Kingdom, and France | 15640 | Technical 4th, Standard care 4th |
| Delmas, 2007 | Effect of Monitoring Bone Turnover Markers on Persistence with Risedronate Treatment of Postmenopausal Osteoporosis | 2302 | Technical 4th, Standard care 4th |
| Derose, 2013 | Automated Outreach to Increase Primary Adherence to Cholesterol-Lowering Medications | 5216 | Educational + Technical 4th, Standard care 4th |
| Edworthy, 2007 | Effects of an enhanced secondary prevention program for patients with heart disease: A prospective randomized trial | 2643 | Educational 4th, Standard care 4th |
| Eron, 2004 | Once-Daily versus Twice-Daily Lopinavir/Ritonavir in Antiretroviral-Naive HIV-Positive Patients: A 48-Week Randomized Clinical Trial | 38 | Technical 4th, Standard care 4th |
| Eussen, 2010 | A Pharmaceutical Care Program to Improve Adherence to Statin Therapy: A Randomized Controlled Trial | 899 | Educational 4th, Standard care 4th |
| Falces, 2008 | [An educative intervention to improve treatment compliance and to prevent readmissions of elderly patients with heart failure] | 103 | Educational 4th, Standard care 4th |
| Gallant, 2006 | Tenofovir DF, Emtricitabine, and Efavirenz vs. Zidovudine, Lamivudine, and Efavirenz for HIV | 509 | Technical 4th, Standard care 4th |
| Gamble, 2011 | A study of a multi-level intervention to improve non-adherence in difficult to control asthma | 18 | Educational + Attitudinal 4th, Standard care 4th |
| Glanz, 2012 | Impact of a Health Communication Intervention to Improve Glaucoma Treatment Adherence: Results of the I-SIGHT Trial | 302 | Educational + Technical 4th, Standard care 4th |
| Goswami, 2013 | Impact of an integrated intervention program on atorvastatin adherence: a randomized controlled trial | 208 | Educational 4th, Standard care 4th |
| Gross, 2013 | Managed Problem Solving for Antiretroviral Therapy Adherence: A Randomized Trial | 180 | Educational + Attitudinal + Technical 4th, Standard care 4th |
| Gurjal, 2014 | Impact of community pharmacist intervention discussing patients’ beliefs to improve medication adherence | 200 | Attitudinal 4th, Standard care 4th |
| Hadji, 2013 | The Patient’s Anastrozole Compliance to Therapy (PACT) Program: a randomized, in-practice study on the impact of a standardized information program on persistence and compliance to adjuvant endocrine therapy in postmenopausal women with early breast cancer† | 2800 | Educational + Technical 4th, Standard care 4th |
| Hawkins, 1979 | Evaluation of a clinical pharmacist in caring for hypertensive and diabetic patients | 137 | Educational 4th, Standard care 4th |
| Hirsch, 2009 | Evaluation of the First Year of a Pilot Program in Community Pharmacy: HIV/AIDS Medication Therapy Management for Medi-Cal Beneficiaries | 7018 | Educational 4th, Standard care 4th |
| Hirsch, 2011 | Antiretroviral Therapy Adherence, Medication Use, and Health Care Costs During 3 Years of a Community Pharmacy Medication Therapy Management Program for Medi-Cal Beneficiaries with HIV/AIDS | 2234 | Educational + Technical 4th, Standard care 4th |
| Ho, 2014 | Multifaceted Intervention to Improve Medication Adherence and Secondary Prevention Measures After Acute Coronary Syndrome Hospital Discharge A Randomized Clinical Trial | 241 | Educational + Technical 4th, Standard care 4th |
| Homer, 2009 | Providing patients with information about disease-modifying antirheumatic drugs: Individually or in groups? A pilot randomized controlled trial comparing adherence and satisfaction | 62 | Educational 4th, Educational + Technical 4th |
| Hornnes, 2011 | Blood Pressure 1 Year after Stroke: The Need to Optimize Secondary Prevention | 293 | Educational + Technical 4th, Standard care 4th |
| Hunt, 2008 | A Randomized Controlled Trial of Team-Based Care: Impact of Physician-Pharmacist Collaboration on Uncontrolled Hypertension | 272 | Educational + Technical 4th, Standard care 4th |
| Johnson, 2011 | Improving Coping Skills for Self-management of Treatment Side Effects Can Reduce Antiretroviral Medication Nonadherence among People Living with HIV | 249 | Educational + Attitudinal 4th, Standard care 4th |
| Joost, 2014 | Intensified pharmaceutical care is improving immunosuppressive medication adherence in kidney transplant recipients during the first post-transplant year: a quasi-experimental study | 74 | Educational + Technical 4th, Standard care 4th |
| Kellaway, 1979 | The effect of counselling on compliance-failure in patient drug therapy | 757 | Educational + Technical 4th, Standard care 4th |
| Kiweewa, 2013 | Noninferiority of a Task-Shifting HIV Care and Treatment Model Using Peer Counselors and Nurses Among Ugandan Women Initiated on ART: Evidence From a Randomized Trial | 85 | Educational 4th, Standard care 4th |
| Konkle-Parker, 2014 | "Effects of an Intervention Addressing Information, Motivation, and Behavioral Skills on HIV Care Adherence in a Southern Clinic Cohort" | 100 | Educational + Attitudinal + Technical 4th, Standard care 4th |
| Kooy, 2013 | Does the use of an electronic reminder device with or without counseling improve adherence to lipid-lowering treatment? The results of a randomized controlled trial | 381 | Technical 4th, Attitudinal + Technical 4th, Standard care 4th |
| Lam, 2003 | A Randomized Controlled Study of Cognitive Therapy for Relapse Prevention for Bipolar Affective Disorder | 103 | Attitudinal 4th, Standard care 4th |
| Lester, 2010 | Effects of a mobile phone short message service on antiretroviral treatment adherence in Kenya (WelTel Kenya1): a randomised trial | 538 | Technical 4th, Standard care 4th |
| Lopez Cabezas, 2006 | Randomized clinical trial of a postdischarge pharmaceutical care program vs. regular follow-up in patients with heart failure | 63 | Educational 4th, Standard care 4th |
| Lucas, 2013 | Directly Administered Antiretroviral Therapy for HIVInfected Individuals in Opioid Treatment Programs: Results from a Randomized Clinical Trial | 107 | Technical 4th, Standard care 4th |
| Malotte, 2001 | Incentives vs Outreach Workers for Latent Tuberculosis Treatment in Drug Users | 163 | Rewards + Technical 4th, Technical 4th |
| Markopoulous, 2015 | Does patient education work in breast cancer? Final results from the global CARIATIDE study | 2757 | Educational 4th, Standard care 4th |
| Molina, 2007 | A Lopinavir/Ritonavir-Based Once-Daily Regimen Results in Better Compliance and Is Non-inferior to a Twice-Daily Regimen Through 96 Weeks | 190 | Technical 4th, Standard care 4th |
| Morisky, 1985 | Evaluation of family health education to build social support for long-term control of high blood pressure. | 290 | Educational 4th, Standard care 4th |
| Morisky, 1990 | A Patient Education Program to Improve Adherence Rates with Antituberculosis Drug Regimens | 88 | Educational + Attitudinal + Rewards 4th, Standard care 4th |
| Moshkovska, 2011 | Impact of a Tailored Patient Preference Intervention in Adherence to 5-Aminosalicylic Acid Medication in Ulcerative Colitis: Results from an Exploratory Randomized Controlled Trial | 71 | Educational + Attitudinal + Technical 4th, Standard care 4th |
| Mugusi, 2009 | Enhancing adherence to antiretroviral therapy at the HIV clinic in resource constrained countries; the Tanzanian experience | 621 | Technical 4th, Educational + Technical 4th, Standard care 4th |
| Munoz, 2009 | Community-based DOT-HAART Accompaniment in an Urban Resource-Poor Setting | 120 | Educational + Technical 4th, Standard care 4th |
| Nielson, 2010 | Patient education in groups increases knowledge of osteoporosis and adherence to treatment: A two-year randomized controlled trial | 300 | Educational 4th, Standard care 4th |
| Ogedegbe, 2008 | A Practice-based Trial of Motivational Interviewing and Adherence in Hypertensive 065African Americans | 160 | Attitudinal 4th, Standard care 4th |
| Ogedegbe, 2012 | A Randomized Controlled Trial of Positive-Affect Intervention and Medication Adherence in Hypertensive African Americans | 256 | Educational 4th, Educational + Attitudinal 4th |
| Pagoto, 2013 | Can attention control conditions have detrimental effects in behavioral medicine randomized trials? | 235 | Educational 4th, Standard care 4th |
| Palacio, 2015 | Can Phone-Based Motivational Interviewing Improve Medication Adherence to Antiplatelet Medications After a Coronary Stent Among Racial Minorities? A Randomized Trial | 339 | Attitudinal 4th, Educational 4th |
| Pearson, 2007 | Randomized Control Trial of Peer-Delivered, Modified Directly Observed Therapy for HAART in Mozambique | 350 | Educational + Technical 4th, Standard care 4th |
| Piette, 2000 | Do Automated Calls with Nurse Follow-up Improve Self-Care and Glycemic Control among Vulnerable Patients with Diabetes? | 248 | Educational + Technical 4th, Standard care 4th |
| Piette, 2001 | Impact of Automated Calls With Nurse Follow-Up on Diabetes Treatment Outcomes in a Department of Veterans Affairs Health Care System A randomized controlled trial | 272 | Educational + Technical 4th, Standard care 4th |
| Pop-Eleches, 2011 | Mobile phone technologies improve adherence to antiretroviral treatment in a resource-limited setting: a randomized controlled trial of text message reminders | 428 | Technical 4th, Standard care 4th |
| Purcell, 2007 | Results From a Randomized Controlled Trial of a Peer-Mentoring Intervention to Reduce HIV Transmission and Increase Access to Care and Adherence | 408 | Attitudinal 4th, Educational 4th |
| Pyne, 2011 | Effectiveness of Collaborative Care for Depression in Human Immunodeficiency Virus Clinics | 178 | Educational + Technical 4th, Standard care 4th |
| Rabenda, 2008a | Adherence to bisphosphonates therapy and hip fracture risk in osteoporotic women | 29157 | Technical 4th, Standard care 4th |
| Rabenda, 2008b | Low Incidence of Anti-Osteoporosis Treatment After Hip Fracture | 306 | Technical 4th, Standard care 4th |
| Reinares, 2008 | Impact of caregiver group psychoeducation on the course and outcome of bipolar patients in remission: a randomized controlled trial | 113 | Educational + Attitudinal 4th, Standard care 4th |
| Reynolds, 2008 | Telephone Support to Improve Antiretroviral Medication Adherence | 109 | Educational 4th, Educational + Attitudinal 4th |
| Rinfret, 2013 | Telephone contact to improve adherence to dual antiplatelet therapy after drug-eluting stent implantation | 300 | Educational 4th, Standard care 4th |
| Sabin, 2010 | Using Electronic Drug Monitor Feedback to Improve Adherence to Antiretroviral Therapy Among HIV-Positive Patients in China | 64 | Educational + Technical 4th, Standard care 4th |
| Sadik, 2005 | Pharmaceutical care of patients with heart failure | 208 | Educational + Technical 4th, Standard care 4th |
| Samet, 2005 | A randomized controlled trial to enhance antiretroviral therapy adherence in patients with a history of alcohol problems | 94 | Educational + Attitudinal + Technical 4th, Standard care 4th |
| Selke, 2010 | Task-Shifting of Antiretroviral Delivery From Health Care Workers to Persons Living With HIV/AIDS: Clinical Outcomes of a Community-Based Program in Kenya | 208 | Educational 4th, Standard care 4th |
| Shet, 2014 | Effect of mobile telephone reminders on treatment outcome in HIV: evidence from a randomised controlled trial in India | 631 | Technical 4th, Standard care 4th |
| Silveira, 2014 | Randomized Controlled Trial to Evaluate the Impact of Pharmaceutical Care on Therapeutic Success in HIV-Infected Patients in Southern Brazil | 332 | Educational 4th, Standard care 4th |
| Soloman, 2012 | Osteoporosis Telephonic Intervention to Improve Medication Adherence (OPTIMA): A Large Pragmatic Randomized Controlled Trial | 2087 | Attitudinal 4th, Educational 4th |
| Sosa, 2005 | Abacavir and Lamivudine Fixed-Dose Combination Tablet | 236 | Technical 4th, Standard care 4th |
| Su and Pergn, 2002 | Fixed-dose combination chemotherapy (Rifater®/Rifinah®) for active pulmonary tuberculosis in Taiwan: a two-year follow-up | 52 | Technical 4th, Standard care 4th |
| Taiwo, 2010 | Assessing the Viorologic and Adherence Benefits of Patient-Selected HIV Treatment Partners in a Resource-limited Setting | 499 | Technical 4th, Standard care 4th |
| Taylor, 2003 | Improving primary care in rural Alabama with a pharmacy initiative | 69 | Educational + Technical 4th, Standard care 4th |
| Thom, 2013 | Effects of a Fixed-Dose Combination Strategy on Adherence and Risk Factors in Patients With or at High Risk of CVD The UMPIRE Randomized Clinical Trial | 1860 | Technical 4th, Standard care 4th |
| Tuldra, 2000 | Prospective Randomized Two-Arm Controlled Study To Determine the Efficacy of a Specific Intervention To Improve Long-Term Adherence to Highly Active Antiretroviral Therapy | 116 | Attitudinal 4th, Standard care 4th |
| Valencia, 2008 | A psychosocial skills training approach in Mexican out-patients with schizophrenia | 82 | Educational 4th, Standard care 4th |
| Varma, 1999 | Pharmaceutical Care of Patients with Congestive Heart Failure: Interventions and Outcomes | 49 | Educational + Technical 4th, Standard care 4th |
| Velligan, 2008 | The Use of Individually Tailored Environmental Supports to Improve Medication Adherence and Outcomes in Schizophrenia | 61 | Attitudinal 4th, Standard care 4th |
| Villeneuve, 2010 | A cluster randomized controlled Trial to Evaluate an Ambulatory primary care Management program for patients with dyslipidemia: the TEAM study | 225 | Educational 4th, Standard care 4th |
| Vollmer, 2014 | Improving Adherence to Cardiovascular Disease Medications With Information Technology | 21752 | Technical 4th, Educational + Technical 4th, Standard care 4th |
| Wagner, 2006 | Cognitive-behavioral intervention to enhance adherence to antiretroviral therapy: a randomized controlled trial (CCTG 578) | 199 | Attitudinal 4th, Standard care 4th |
| Wang, 2011 | Effects of pharmaceutical care interventions on blood pressure and medication adherence of patients with primary hypertension in China | 59 | Educational + Technical 4th, Standard care 4th |
| Weber, 2004 | Effect of individual cognitive behaviour intervention on adherence to antiretroviral therapy: prospective randomized trial | 60 | Attitudinal 4th, Standard care 4th |
| Williams, 2006 | Home Visits to Improve Adherence to Highly Active Antiretroviral Therapy: A Randomized Controlled Trial | 171 | Educational + Attitudinal 4th, Standard care 4th |
| Williams, 2014 | Efficacy of an Evidence-Based ARV Adherence Intervention in China | 110 | Educational + Attitudinal 4th, Standard care 4th |
| Windsor, 1990 | Evaluation of the Efficacy and Cost Effectiveness of Health Education Methods to Increase Medication Adherence among Adults with Asthma | 267 | Educational 4th, Standard care 4th |
| Xavier, 2016 | "Community health worker-based intervention for adherence to drugs and lifestyle change after acute coronary syndrome: a multicentre, open, randomised controlled trial" | 750 | Educational + Technical 4th, Standard care 4th |
| Ziller, 2013 | Influence of a patient information program on adherence and persistence with an aromatase inhibitor in breast cancer treatment - the COMPAS study | 171 | Educational 4th, Educational + Technical 4th, Standard care 4th |
| Zillich, 2012 | Evaluation of Specialized Medication Packaging Combined With Medication Therapy Management: Adherence, Outcomes, and Costs Among Medicaid Patients | 14621 | Educational 4th, Standard care 4th |
| Zwikker, 2014 | Effectiveness of a group-based intervention to change medication beliefs and improve medication adherence in patients with rheumatoid arthritis: A randomized controlled trial | 123 | Attitudinal 4th, Educational 4th |
