# Supplementary figures and images for: Temporal effectiveness of interventions to improve medication adherence: A network meta-analysis
Source: PLoS One. 2019 Mar 12;14(3):e0213432. doi: 10.1371/journal.pone.0213432 (PMC6413898; doi:10.1371/journal.pone.0213432)

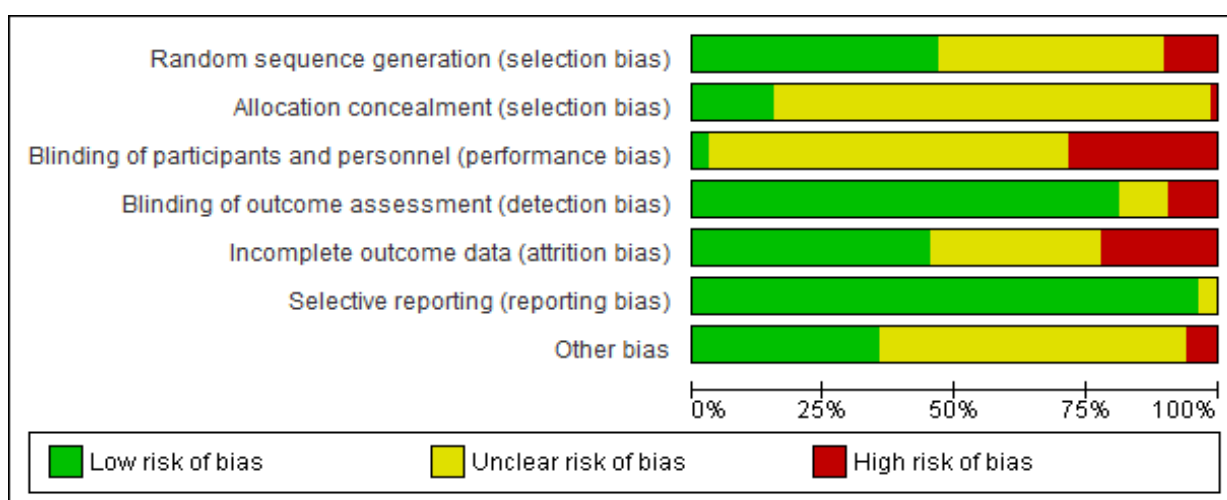

Supplement: S1 Fig — (PDF) [file pone.0213432.s008.pdf]
