# Supplementary material for: Temporal effectiveness of interventions to improve medication adherence: A network meta-analysis
Source: PLoS One. 2019 Mar 12;14(3):e0213432. doi: 10.1371/journal.pone.0213432 (PMC6413898; doi:10.1371/journal.pone.0213432)
Supplement: S2 Fig — (DOCX) [file pone.0213432.s009.docx]

**S2 Figure. SUCRA analyses**

**0-3 months**

**4-6 months**

**7-9 months**

**≥10 months**
