# Supplementary material for: Temporal effectiveness of interventions to improve medication adherence: A network meta-analysis
Source: PLoS One. 2019 Mar 12;14(3):e0213432. doi: 10.1371/journal.pone.0213432 (PMC6413898; doi:10.1371/journal.pone.0213432)
Supplement: S1 File — (DOCX) [file pone.0213432.s010.docx]

**S1 File. Sensitivity and sub-group analyses**

**Figure A and Tables A-D. Sample size >30**

**Fig A. Networks of the comparisons between interventions with sample size >30 for each time period (0-3 months, 4-6 months, 7-9 months, ≥10 months) (top left to bottom right) considering the overall composite measure of adherence.** Each node represents an intervention. Directly comparable interventions are linked with a line, the number of trials for each comparison are shown in each line.


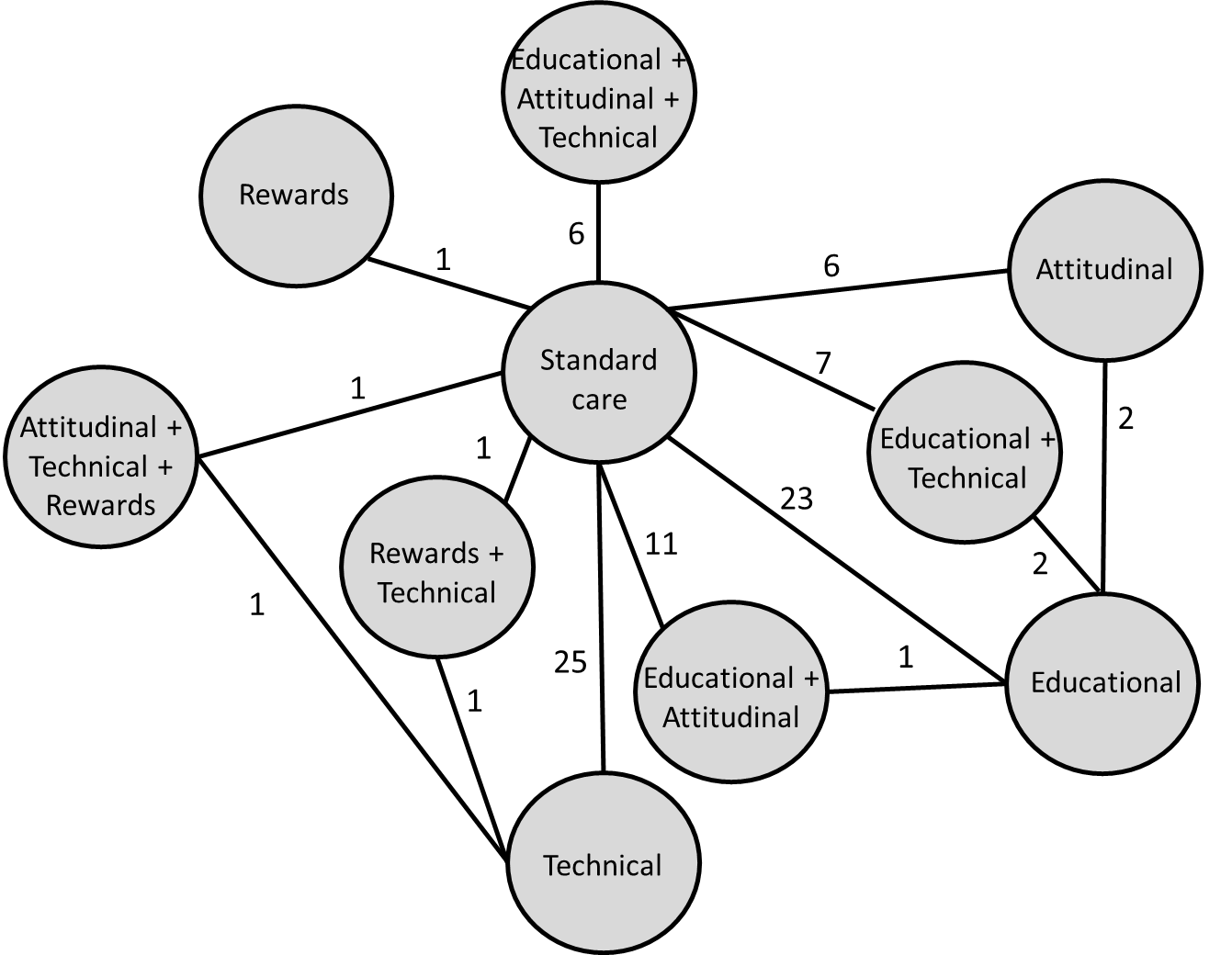

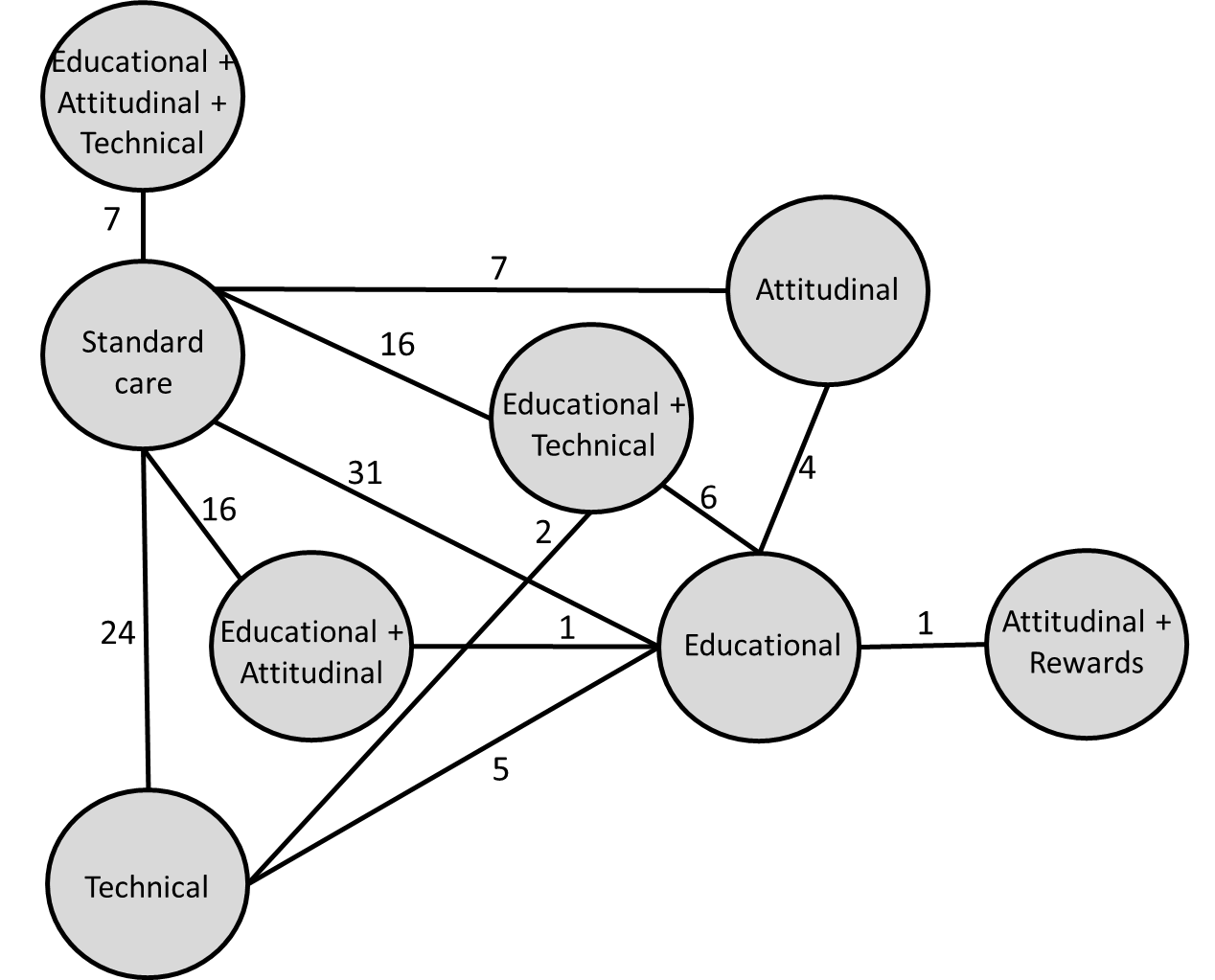


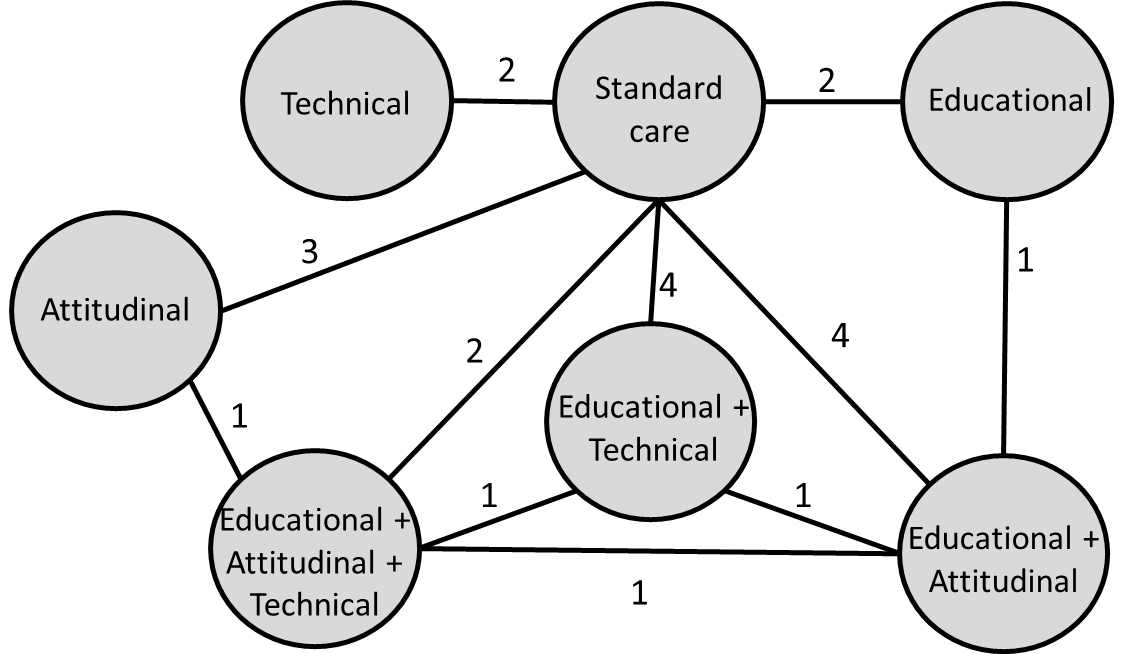

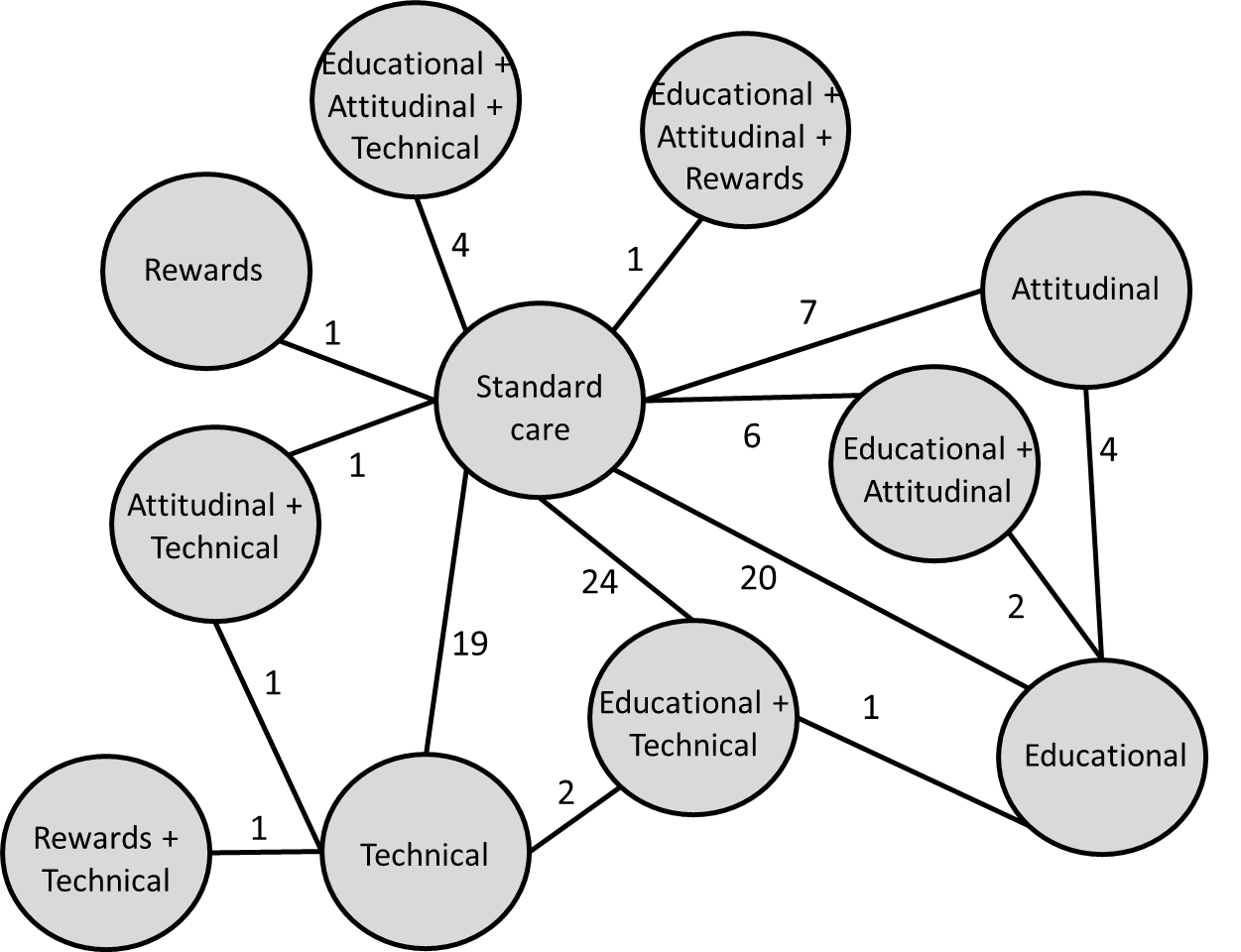


**Tables A-D. Consistency analyses of multiple comparison analyses for the overall composite measure in studies with sample size >30 in 0-3 months, 4-6 months, 7-9 months, and ≥10 months.**

Effect sizes are reported as OR (with 95% CrI). Comparisons are read from left to right (row to column above, column to row below). An OR <1 indicates a more effective intervention. Bold data comparisons are statistically significant. Edu: educational, Att: attitudinal, Tec: technical, Rew: rewards, SOC: standard of care.

**Table A. 0-3 months**

**Table B. 4-6 months**

**Table C. 7-9 months**

**Table D. ≥10 months**

**Figure B and Tables E-H. After 2007**

**Fig B. Networks of the comparisons between interventions in studies published after 2007 for each time period (0-3 months, 4-6 months, 7-9 months, ≥10 months) (top left to bottom right) considering the overall composite measure of adherence.** Each node represents an intervention. Directly comparable interventions are linked with a line, the number of trials for each comparison are shown in each line.


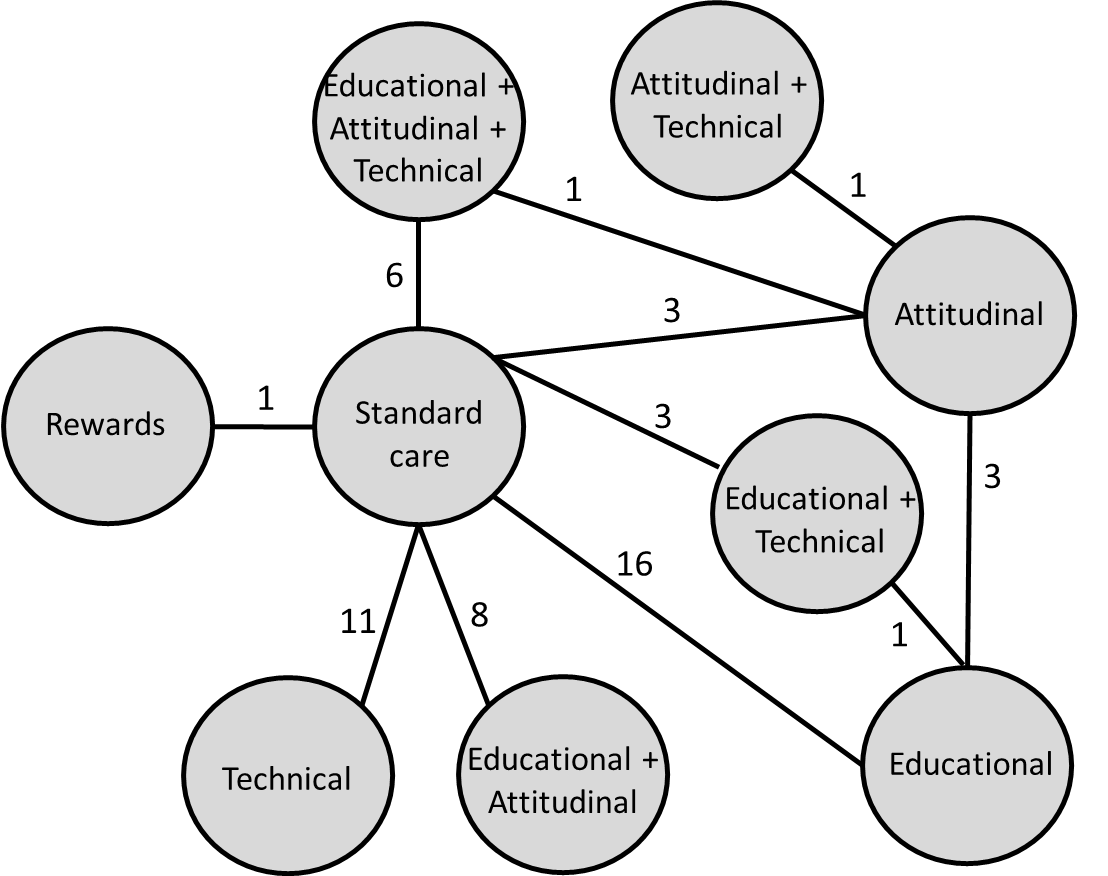

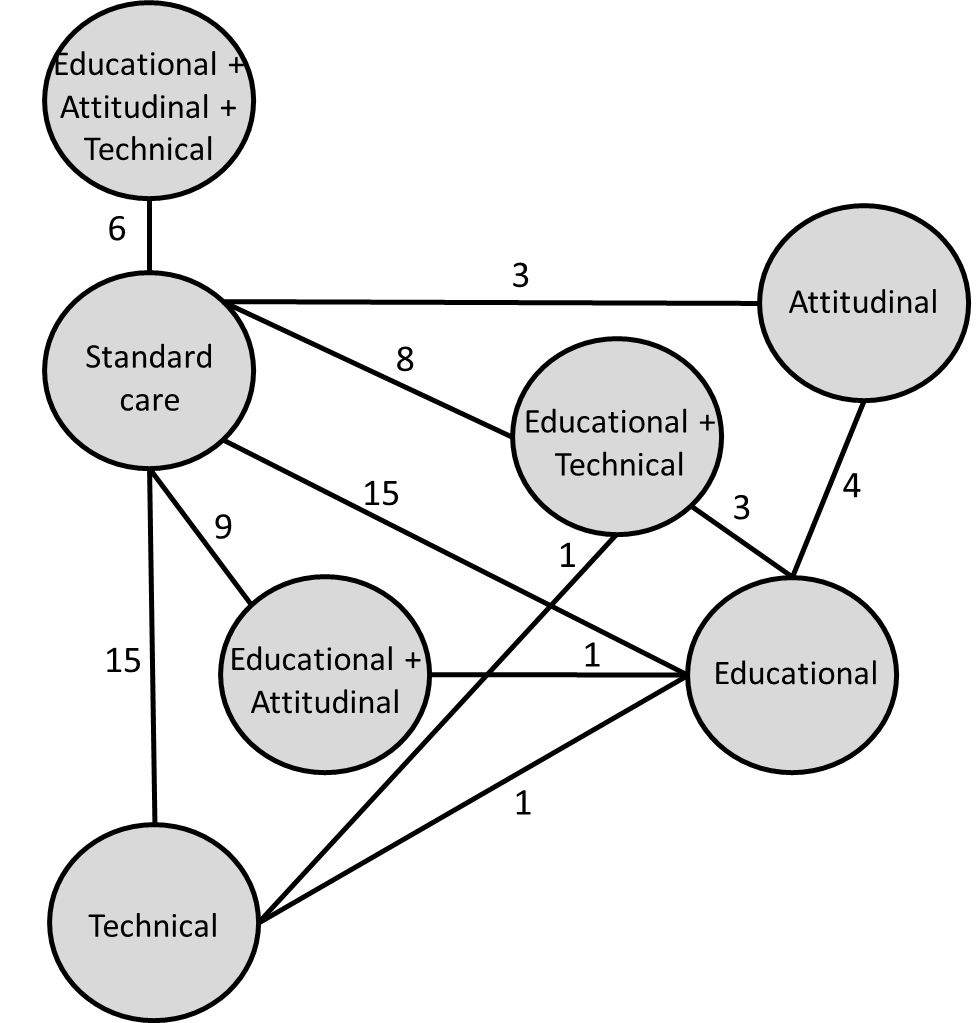


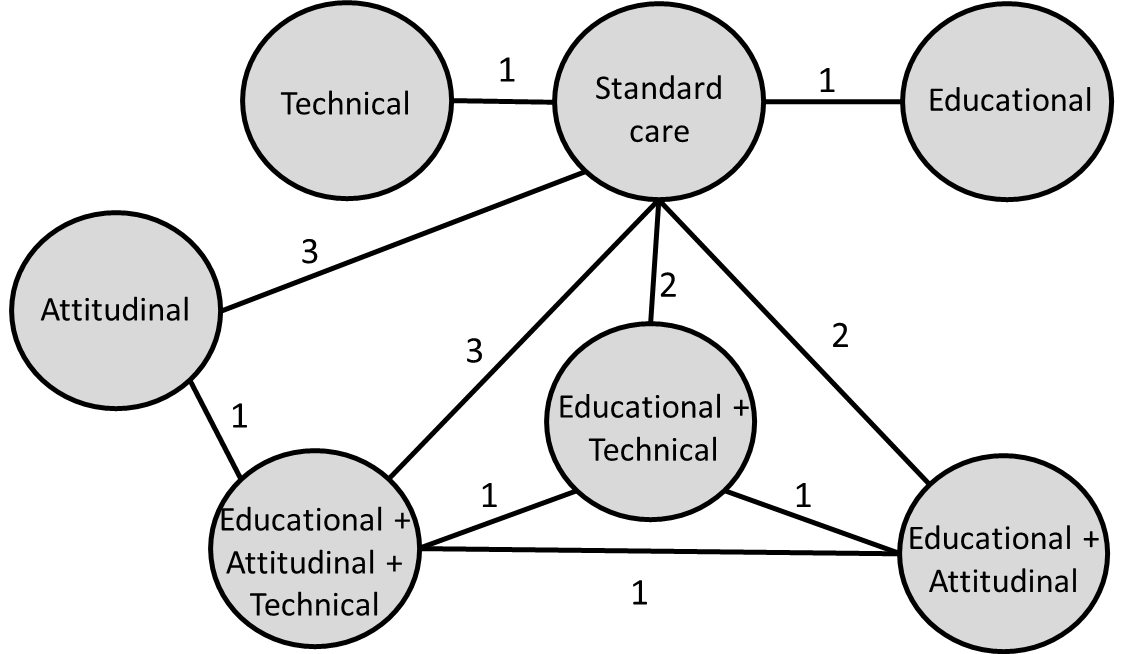

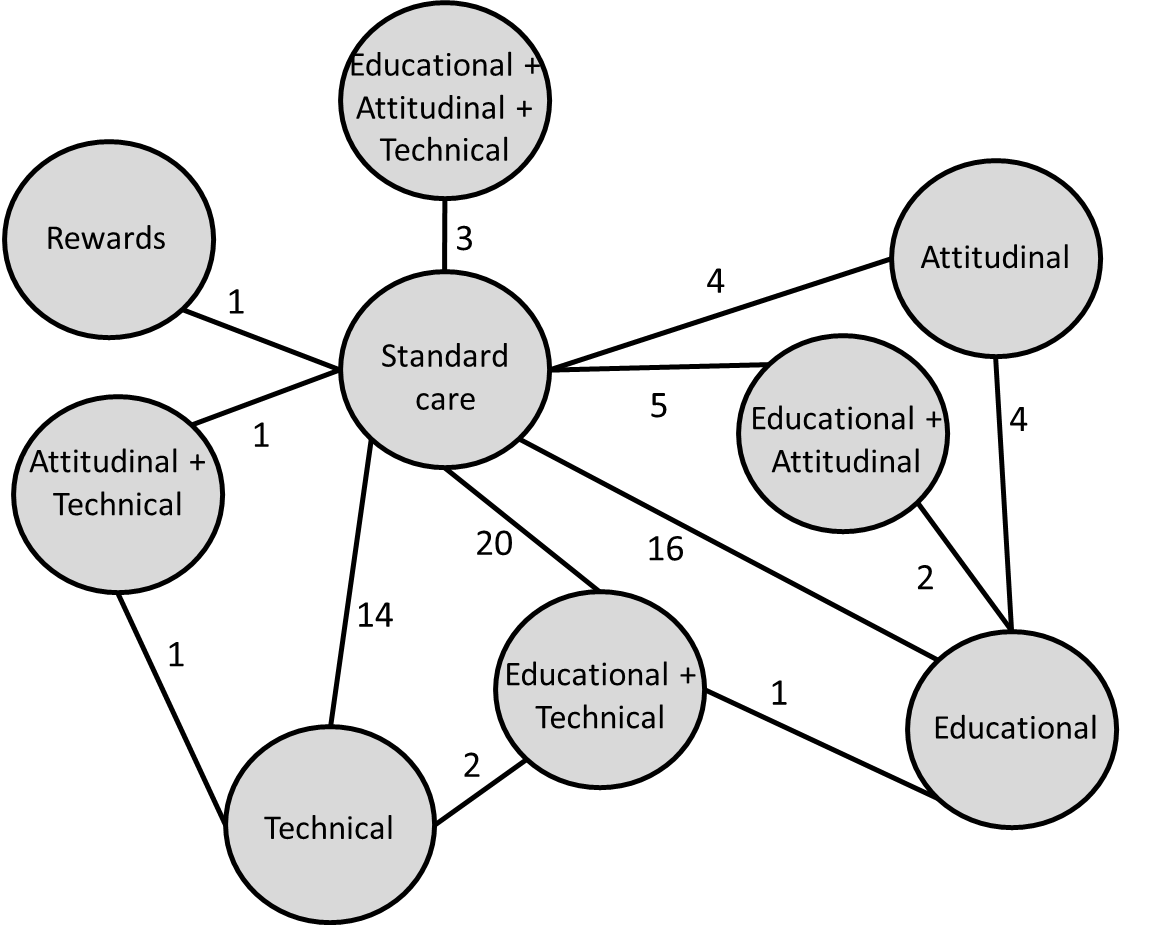


**Tables E-H. Consistency analyses of multiple comparison analyses for the overall composite measure in studies published after 2007 in 0-3 months, 4-6 months, 7-9 months, and ≥10 months.**

Effect sizes are reported as OR (with 95% CrI). Comparisons are read from left to right (row to column above, column to row below). An OR <1 indicates a more effective intervention. Bold data comparisons are statistically significant. Edu: educational, Att: attitudinal, Tec: technical, Rew: rewards, SOC: standard of care.

**Table E. 0-3 months**

**Table F. 4-6 months**

**Table G. 7-9 months**

**Table H. ≥10 months**

**Figure C and Tables I-L. Before 2007**

**Fig C. Networks of the comparisons between interventions in studies published before 2007 for each time period (0-3 months, 4-6 months, 7-9 months, ≥10 months) (top left to bottom right) considering the overall composite measure of adherence.** Each node represents an intervention. Directly comparable interventions are linked with a line, the number of trials for each comparison are shown in each line.


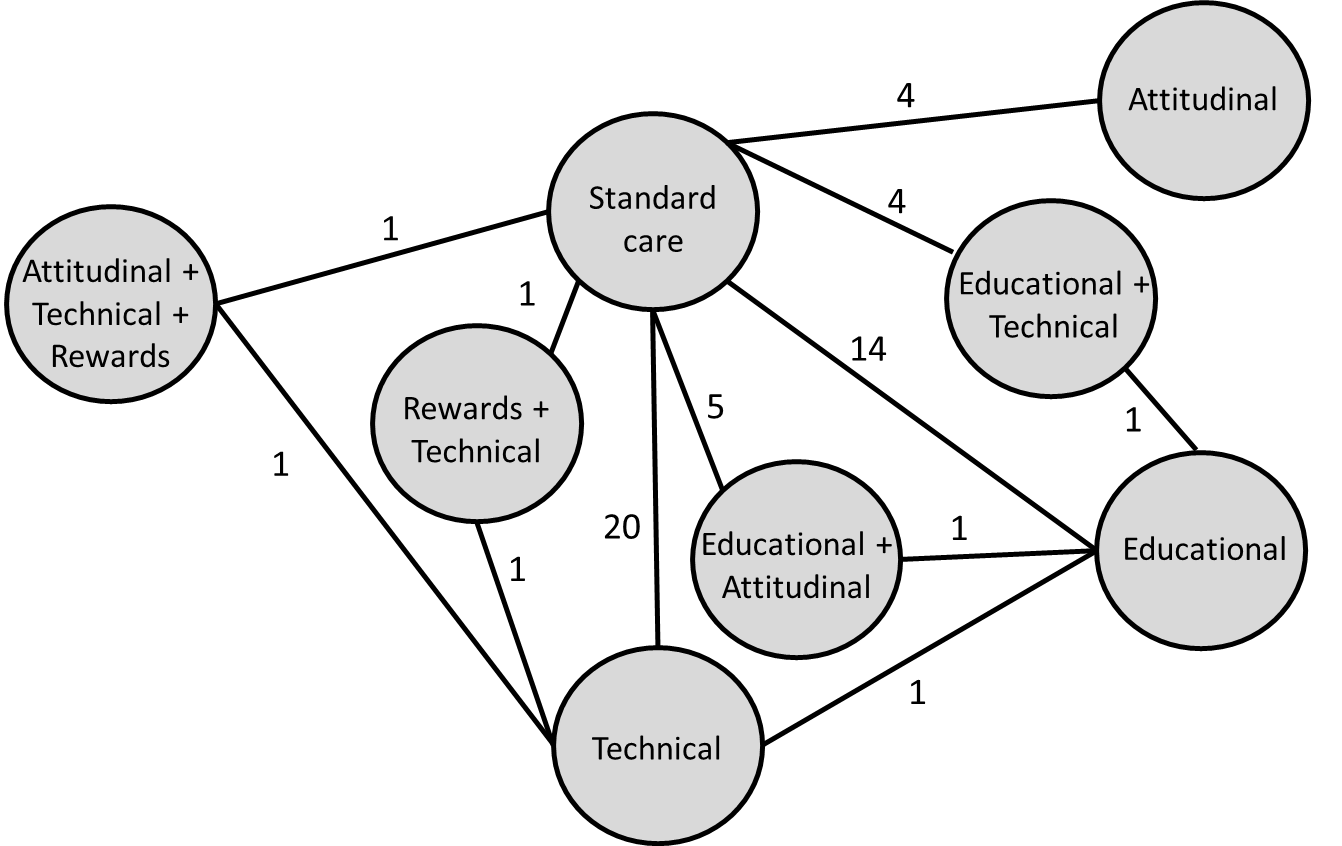

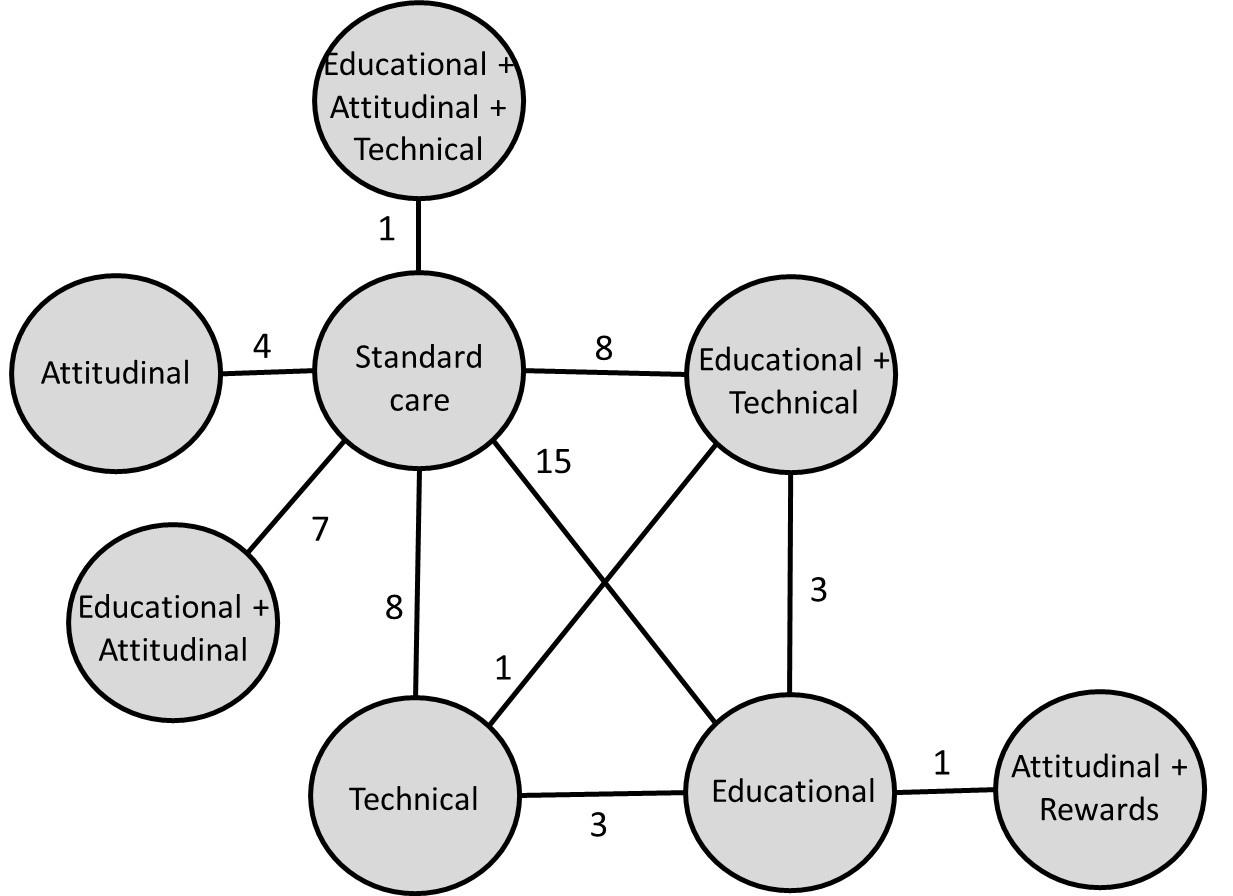


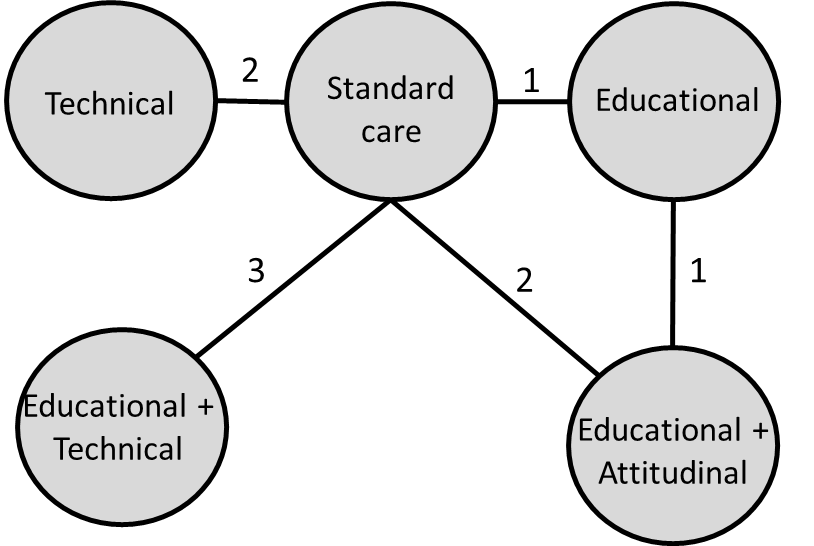

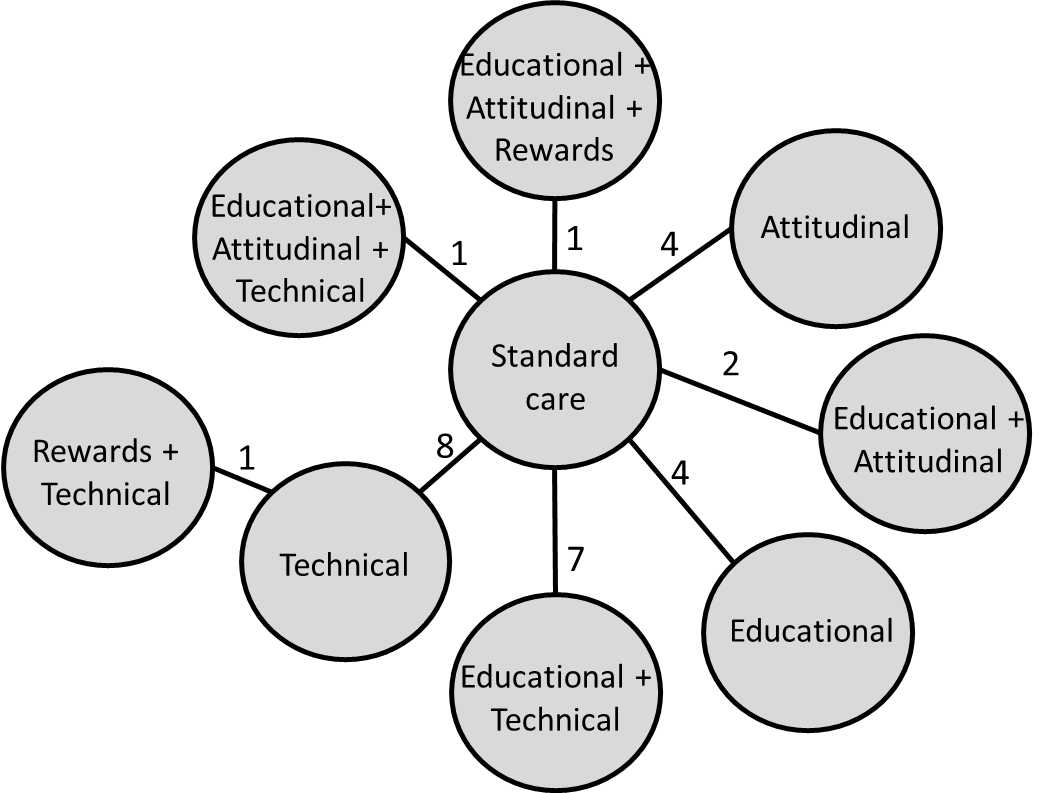


**Tables I-L. Consistency analyses of multiple comparison analyses for the overall composite measure in studies published before 2007 in 0-3 months, 4-6 months, 7-9 months, and ≥10 months.**

Effect sizes are reported as OR (with 95% CrI). Comparisons are read from left to right (row to column above, column to row below). An OR <1 indicates a more effective intervention. Bold data comparisons are statistically significant. Edu: educational, Att: attitudinal, Tec: technical, Rew: rewards, SOC: standard of care.

**Table I. 0-3 months**

**Table J. 4-6 months**

**Table K. 7-9 months**

**Table L. ≥10 months**
